# Novel [1,2,4]triazolo[3,4-*b*][1,3,4]thiadiazine and [1,2,4]triazolo[3,4-*b*][1,3,4]thiadiazepine Derivatives: Synthesis, Anti-Viral In Vitro Study and Target Validation Activity

**DOI:** 10.3390/molecules27227940

**Published:** 2022-11-16

**Authors:** Andrey V. Khramchikhin, Mariya A. Skryl’nikova, Iana L. Esaulkova, Ekaterina O. Sinegubova, Vladimir V. Zarubaev, Maxim A. Gureev, Aleksandra M. Puzyk, Vladimir A. Ostrovskii

**Affiliations:** 1 Department of Organic Chemistry, Saint Petersburg State Institute of Technology, Technical University, 26 Moskovsky Avenue, 190013 St. Petersburg, Russia; 2 Department of Chemistry and Technology of Organic Nitrogen Compounds, Saint Petersburg State Institute of Technology, Technical University, 26 Moskovsky Avenue, 190013 St. Petersburg, Russia; 3 Saint Petersburg Pasteur Research Institute of Epidemiology and Microbiology, 14 Mira Street, 197101 St. Petersburg, Russia; 4 Center of Bio and Cheminformatics, I. M. Sechenov First Moscow State Medical University, Trubetskaya 8/2, 119991 Moscow, Russia; 5 Research Park, Saint Petersburg State University, 26, Universitetskii Prospect, Petergof, 198504 St. Petersburg, Russia; 6 Saint Petersburg Federal Research Center of the Russian Academy of Sciences (SPC RAS), 39, 14th Line, 199178 St. Petersburg, Russia

**Keywords:** drug design, annelated heterocycles, influenza virus, anti-viral activity, [1,2,4]triazolo[3,4-*b*][1,3,4]thiadiazines, [1,2,4]triazolo[3,4-*b*][1,3,4]thiadiazepines

## Abstract

This study of the interaction system of binucleophilic 3-substituted 4-amino-4*H*-1,2,4-triazole-5-thiols and 3-phenyl-2-propynal made it possible to develop a new approach to synthesis of such isomeric classes as 7-benzylidene-[1,2,4]triazolo[3,4-*b*][1,3,4]thiadiazine and 8-phenyl-[1,2,4]triazolo[3,4-*b*][1,3,4]thiadiazepine. Among the 20 compounds studied in vitro against influenza A/Puerto Rico/8/34 (H1N1) virus, half of them demonstrated selectivity index (SI) of 10 or higher and one of them (4-((3-phenylprop-2-yn-1-yl)amino)-4*H*-1,2,4-triazole-3-thiol) possessed the highest (SI > 300). Docking results and values showed that the preferred interactant for our ligands was M2 proton channel of the influenza A virus. Protein-ligand interactions modeling showed that the aliphatic moiety of ligands could negatively regulate target activity level.

## 1. Introduction

The importance of the development of new anti-viral drugs, as a rule, is determined by the ability of viruses to elude the immune response of the body and develop drug-resistant strains [1]. In the past 15 years, the world has been shaken by such pandemic dangerous viruses as the Ebola virus [2], Zika virus [3], influenza A virus [4,5] and coronavirus (SARS-CoV-2) [6,7]. Our attention was drawn to the influenza A (H1N1) virus, which is one of the most dangerous due to its rapid mutation [8].

**Figure 1:**
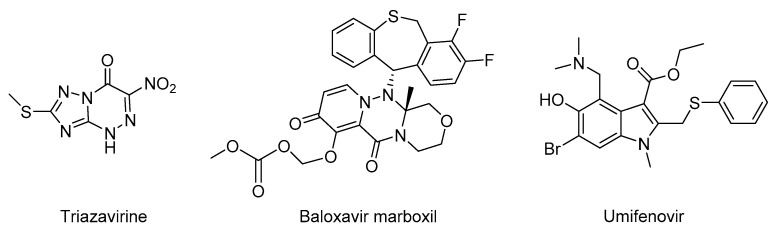
Active pharmaceutical ingredients of drugs used in influenza chemotherapy.

The problem of drugs’ availability against various influenza viruses can be solved by the efficiency of the search for new drugs, among which are known heterocyclic compounds of a series of azoles and azines (Figure 1) containing exo- and endocyclic sulfur atoms [9,10,11,12].

**Figure 2:**
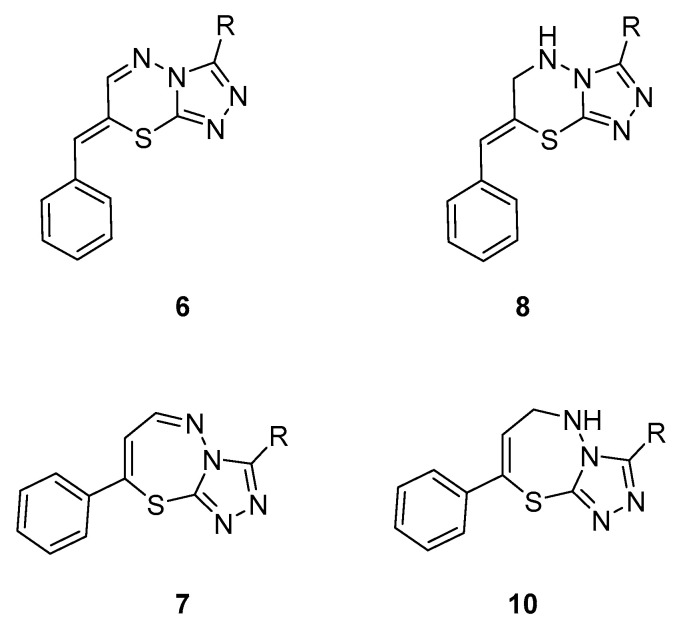
Target compounds: 3-substituted (*Z*)-7-benzylidene-*7H*-[1,2,4]triazolo[3,4-*b*][1,3,4]thiadiazines **6**, 3-substituted (*Z*)-7-benzylidene-6,7-dihydro-5*H*-[1,2,4]triazolo[3,4-*b*][1,3,4]thiadiazines **8**, 3-substituted 8-phenyl-[1,2,4]triazolo[3,4-*b*][1,3,4]thiadiazepines **7** and 3-substituted 8-phenyl-5,6-dihydro-[1,2,4]triazolo[3,4-*b*][1,3,4]thiadiazepines **10**.

In this regard, it was of great interest to synthesize and study the anti-viral activity of annelated heterocyclic systems containing 1,2,4-triazolyl and thiadiazine or thiadiazepine moieties (Figure 2). Such compounds have attracted the attention of researchers with their reactivity and biological activity [13,14,15,16,17,18,19,20,21,22,23,24,25]. At the same time, data on their anti-viral activity are limited [26,27,28].

A major part of our work has been the development of new routes for the synthesis of target compounds (Figure 2).

**Figure 3:**
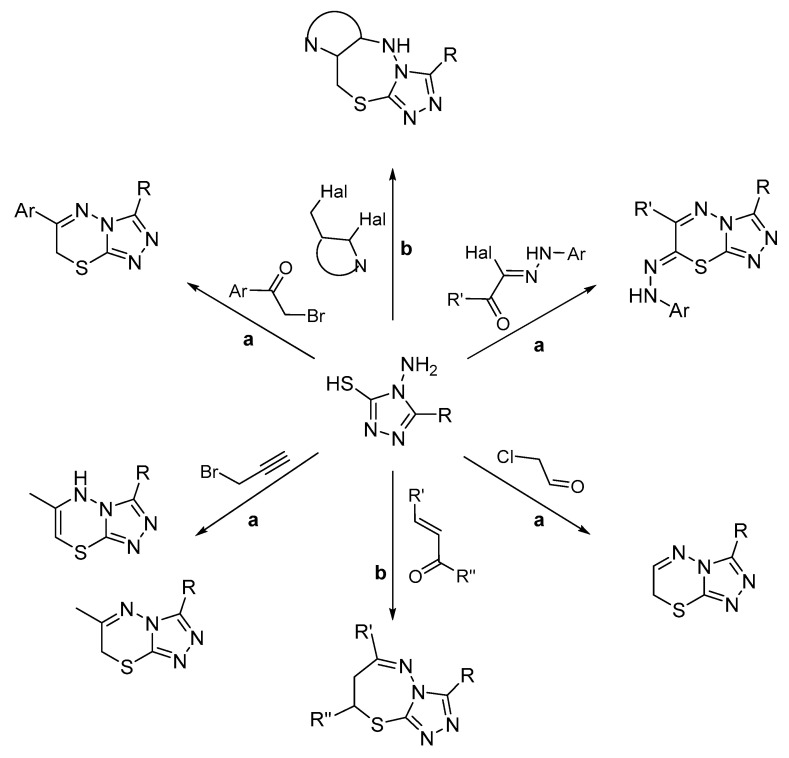
The most common synthetic routes for target compounds: (**a**) [1,2,4]triazolo[3,4-*b*][1,3,4]thiadiazines and (**b**) [1,2,4]triazolo[3,4-*b*][1,3,4]thiadiazepines.

The most common synthesis methods include the interaction of 4-amino-4*H*-1,2,4-triazole-3-thiol with bielectrophiles such as phenacyl bromides [29,30], hydrazonoyl halides [31], chloroacetaldehyde [32], propargyl bromide [33] to produce [1,2,4]triazolo[3,4-*b*][1,3,4]thiadiazines [34] and α,β-ethylene ketones [25], α,γ-dihalogen derivatives [23] to produce [1,2,4]triazolo[3,4-*b*][1,3,4]thiadiazepines (Figure 3). However, the use of a triple bond for cyclization at the mercapto group of 4-amino-4*H*-1,2,4-triazole-3-thiol to produce [1,2,4]triazolo[3,4-*b*][1,3,4]thiadiazines has not been previously studied.

**Figure 4:**
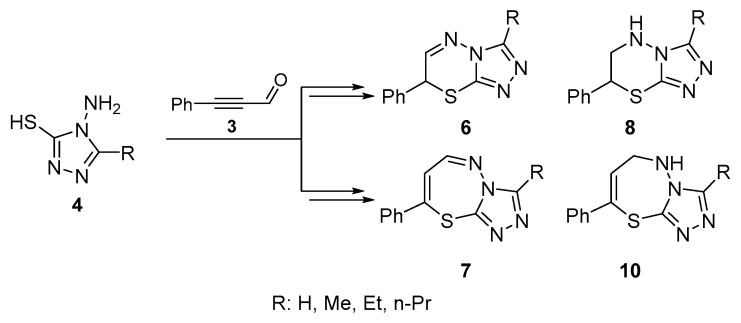
The proposed synthetic routes for target compounds: [1,2,4]triazolo[3,4-*b*][1,3,4]thiadiazines **6**, **8** and [1,2,4]triazolo[3,4-*b*][1,3,4]thiadiazepines **7**, **10**.

We proposed a new synthesis route based on the interaction of 3-phenyl-2-propynal **3** and 3-substituted 4-amino-4*H*-1,2,4-triazole-5-thiols **4** to obtain both triazolothiadiazines **6**, **8** and triazolothiadiazepines **7**, **10** (Figure 4). A more detailed analysis of the synthesis pathways is presented in the discussion of the results.

In the present work, the synthesized compounds were studied in vitro against the influenza A/Puerto Rico/8/34 (H1N1) virus to determine their ability to effectively block the active site of the M2 protein and inhibit the neuraminidase enzyme. To establish a preliminary structure–activity relationship, the in vitro activity obtained was validated using the Schrodinger Suite 2022-2 program.

## 2. Results and Discussion

### 2.1. Chemistry

#### 2.1.1. Synthesis of Target Compounds

**Figure 5:**
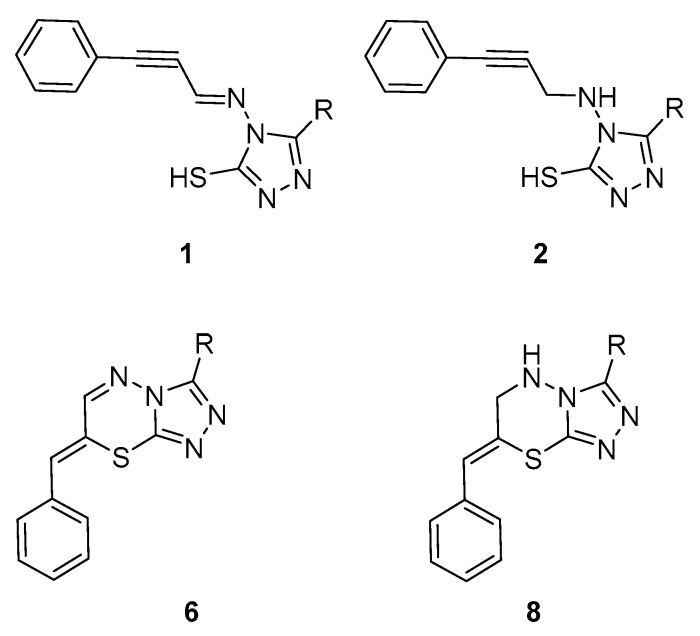
Model (**1**, **2**) and target (**6**, **8**) compounds: 5-substituted 4-((3-phenylprop-2-yn-1-ylidene)amino)-4*H*-1,2,4-triazol-3-thiols **1** and 5-substituted 4-((3-phenylprop-2-yn-1-yl)amino)-4*H*-1,2,4-triazole-3-thiols **2**, 3-substituted (*Z*)-7-benzylidene-7*H*-[1,2,4]triazolo[3,4-*b*][1,3,4]thiadiazines **6**, 3-substituted (*Z*)-7-benzylidene-6,7-dihydro-5*H*-[1,2,4]triazolo[3,4-*b*][1,3,4]thiadiazines **8**.

**Scheme 1:**
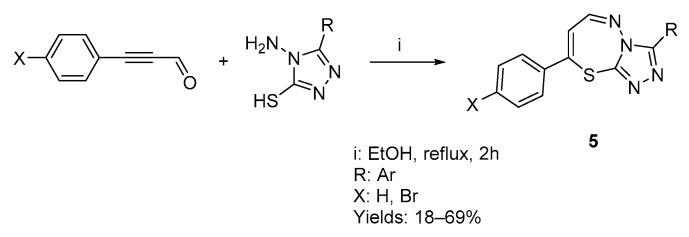
Methodical synthesis of triazolothiadiazepines **5**.

5-substituted 4-((3-phenylprop-2-yn-1-ylidene)amino)-4*H*-1,2,4-triazol-3-thiols **1** and 5-substituted 4-((3-phenylprop-2-yn-1-yl)amino)-4*H*-1,2,4-triazole-3-thiols **2** were chosen as models (Figure 5), since it is well known that thiols undergo nucleophilic addition to triple bond:

Azomethines **1** are formally the condensation products of 3-phenyl-2-propynal **3** and 4-amino-4*H*-1,2,4-triazole-3-thiols **4**. However, prior to our studies, only one article on the interaction of these reagents was published [35]. As a result of the reactions, triazolothiadiazepines **5** in refluxing ethanol were obtained (Scheme 1).

**Scheme 2:**
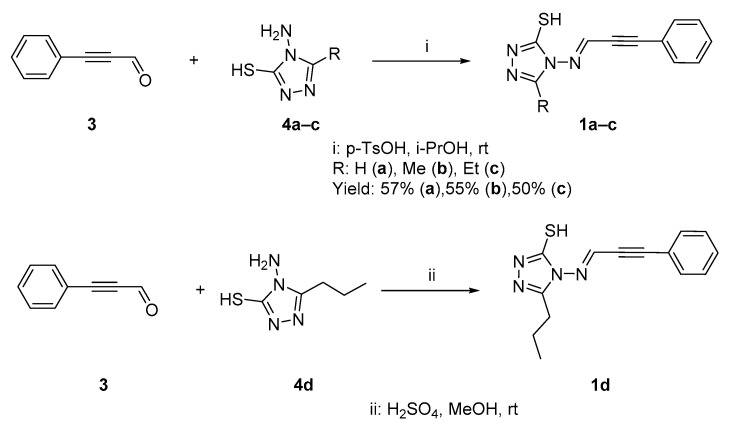
Synthesis of aldimines **1a–d**.

We succeeded in preparing model compounds **1** under milder conditions (room temperature) using TsOH as a catalyst (we reported earlier about compound **1b** synthesis [36]) (Scheme 2). Compound **1d** was used in situ without further isolation.

**Scheme 3:**
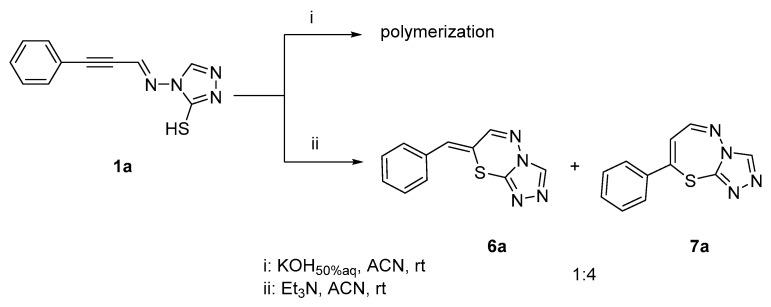
Cyclization attempt of compound **1a**.

**Figure 6:**
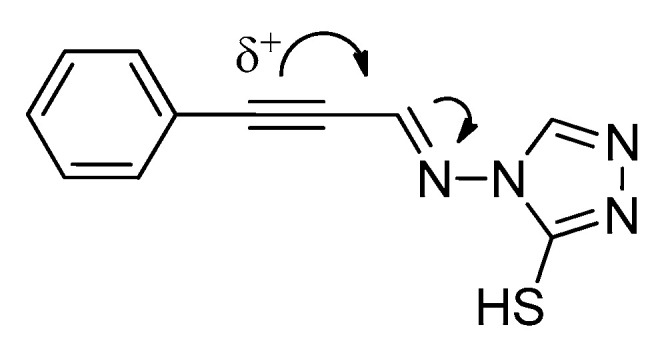
Polarization of the conjugate system of the compound **1a**.

The cyclization of aldimine **1a** was carried out in acetonitrile at room temperature (Scheme 3). In the case of using 50% potassium hydroxide solution as a catalyst, polymerization occurred and a dark viscous liquid was formed. At the same time, when using twofold molar excess of triethylamine, the reaction was completed in 2 h with the formation of a mixture of two substances. According to the NMR spectrum data, we unambiguously identified the structures as **6a** and **7a** in a ratio of 1:4, respectively:

The predominant formation of triazolothiadiazepine **7a** most likely occurs due to the polarization of conjugated multiple bonds system, resulting in the formation of a significant partial positive charge on the fourth atom of the azaenyne system of compound **1a** (Figure 6).

The obtained preliminary result showed the unacceptability of this method as synthetic due to the formation of a mixture of isomers **6a** and **7a**.

**Scheme 4:**
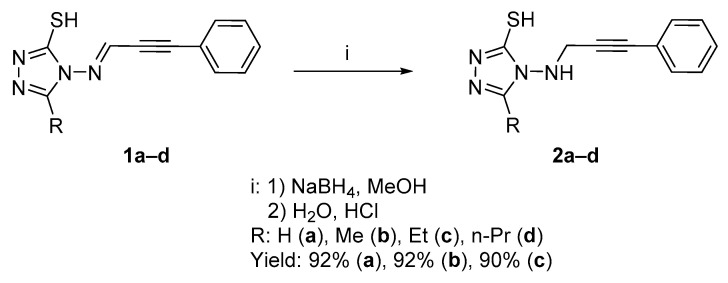
Synthesis of amines **2a–d**.

Primary information on the cyclization of 5-substituted 4-((3-phenylprop-2-yn-1-yl)amino)-4*H*-1,2,4-triazole-3-thiols **2** was obtained using compound **2a**. Compounds **2** themselves were synthesized by hydrogenation of the azomethine fragment of aldimines **1** using sodium borohydride in methanol (Scheme 4).

**Scheme 5:**
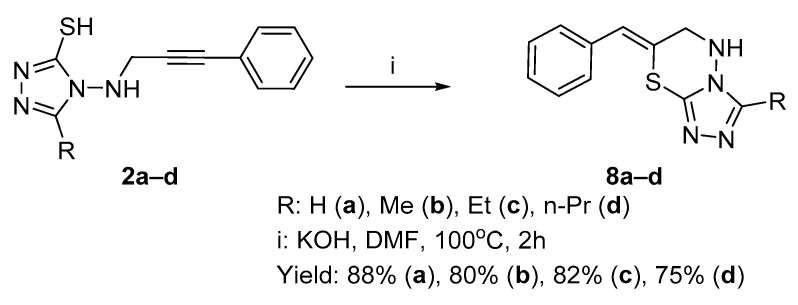
Synthesis of triazolothiadiazines **8a–d**.

Cyclization of α,β-acetylenic amine **2a** was carried out in DMF at 100 °C for 2 h using two drops of 50% aqueous potassium hydroxide solution as a catalyst (Scheme 5). As a result, 7-benzylidene-6,7-dihydro-5*H*-[1,2,4]triazolo[3,4-*b*][1,3,4]thiadiazine **8a** was obtained in high yield.

The cyclization of compounds **2b–d** was carried out under the same conditions and led to the formation of the corresponding triazolothiadiazines **8b–d**. When trying to use triethylamine as a catalyst, no cyclization was indicated, and in the case of using a) a molar excess of potassium carbonate or b) an equimolar amount of potassium hydroxide, polymerization of the starting material was observed.

**Scheme 6:**
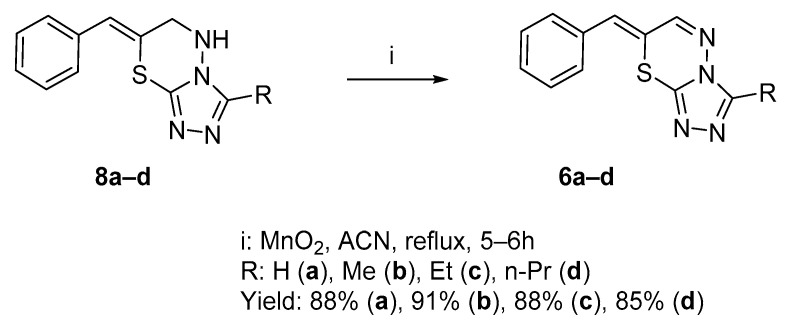
Synthesis of triazolothiadiazines **6a–d**.

Due to the impracticality of obtaining 3-substituted 7-benzylidene-7*H*-[1,2,4]triazolo[3,4-*b*][1,3,4]thiadiazines **6** by cyclization of the corresponding α,β-acetylenic aldimines **1**, we assumed the possibility of their synthesis by dehydrogenation of the C-N bond of the thiadiazine moiety of compounds **8** (Scheme 6). To implement this assumption, active manganese dioxide was chosen.

The reaction was carried out with efficient stirring in refluxing acetonitrile in the presence of active MnO_2_ for 5–6 h. The reaction progress was monitored by TLC, and the yields were 85–91%.

**Scheme 7:**
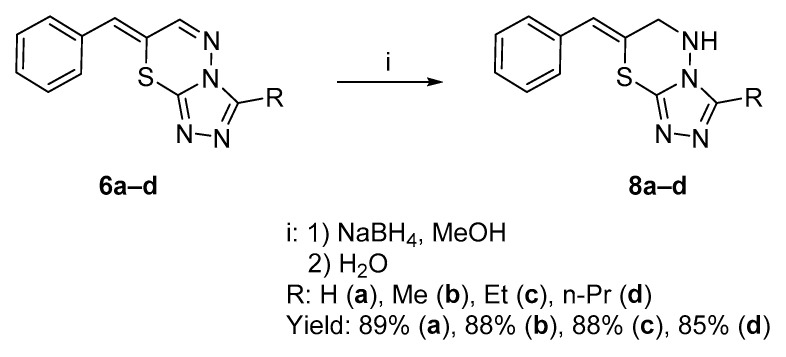
Principle synthesis of triazolothiadiazines **8a–d** from triazolothiadiazines **6a–d**.

We also demonstrated the possibility of synthesizing 3-substituted 7-benzylidene-6,7-dihydro-5*H*-[1,2,4]triazolo[3,4-*b*][1,3,4]thiadiazines **8a–d** by hydrogenation 3-substituted 7-benzylidene-7*H*-[1,2,4]triazolo[3,4-*b*][1,3,4]thiadiazines **6a–d** (Scheme 7). Compounds **8a-d** were obtained in high yields of 85–89%.

**Scheme 8:**
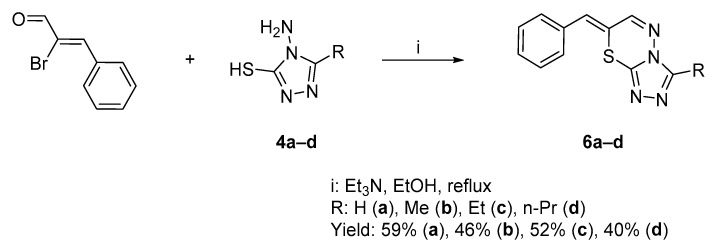
Counter synthesis of triazolothiadiazines **6a–d** from α-bromocinnamic aldehyde.

We also managed to obtain compounds **6a–d** by counter synthesis according to the existing procedure [22]. Samples synthesized by oxidation of compounds **8** and using 2-bromocinnamic aldehyde (Scheme 8) had identical melting points and spectral characteristics. Mixing testing did not give any depression of the melting point.

**Scheme 9:**
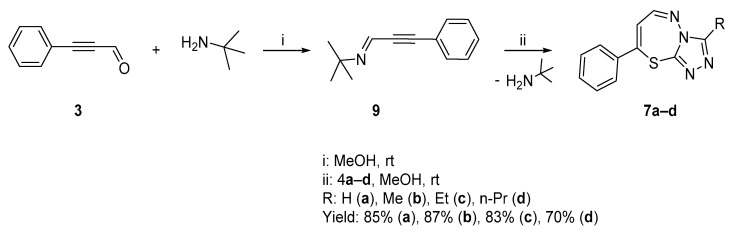
Synthesis of triazolothiadiazepines **7a–d**.

Previously, we obtained 8-phenyl-[1,2,4]triazolo[3,4-*b*][1,3,4]thiadiazepines **7a–b** from 3-phenyl-2-propynal **3** and 4-amino-4*H*-1,2,4-triazole-5-thiols **4a–b** using tert-butylamine under mild conditions—at room temperature [37]. *tert*-Butylamine was added to 3-phenyl-2-propynal **3**. Additionally, 3-substituted 4-amino-4*H*-1,2,4-triazole-5-thiols **4a–d** was added to a solution of the resulting azomethine **9**, which led to the formation of 3-substituted 8-phenyl-[1,2,4]triazolo[3,4-*b*][1,3,4]thiadiazepines **7a–d** (Scheme 9).

**Figure 7:**
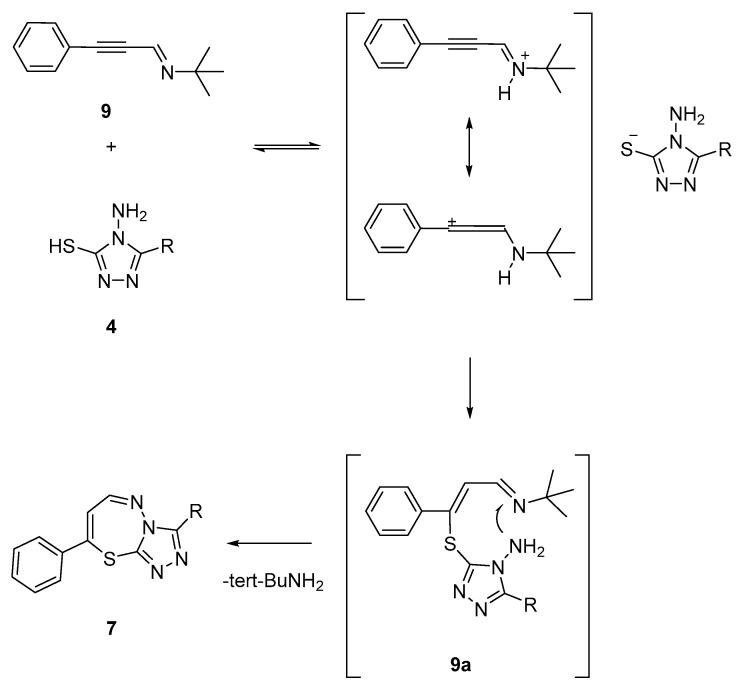
Proposed reaction mechanism for the formation of triazolothiadiazepines **7**.

The formation of the individual compound thiadiazepine **7** can be explained by the initial attack by the sulfur atom of aminotriazolthiol **4** on the carbon atom (having a partial positive charge) of the protonated conjugated system of azomethine **9**, followed by the substitution of tert-butylamine by the amino group of intermediate **9a** (Figure 7).

The reaction temperature decrease from 78 °C (in the case of using 3-phenyl-2-propynal [35]) to room temperature can be explained by the protonation of the nitrogen atom of azomethine **9** with triazolylthiol **4** at the prereaction stage (Figure 7).

**Scheme 10:**
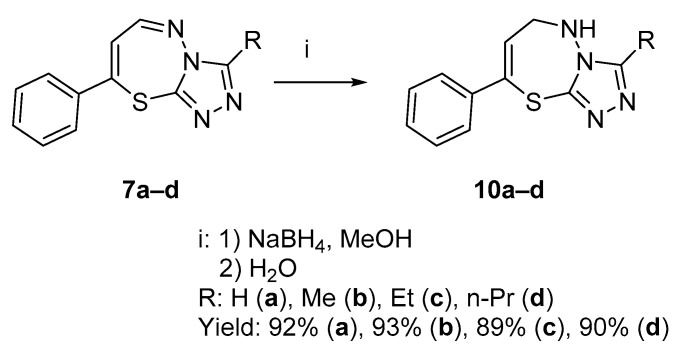
Synthesis of triazolothiadiazepines **10a–d**.

Compounds **7a–d** were treated with sodium borohydride to give 3-substituted 8-phenyl-5,6-dihydro-[1,2,4]triazolo[3,4-*b*][1,3,4]thiadiazepines **10a–d** (Scheme 10).

#### 2.1.2. Structural Analysis

**Figure 8:**
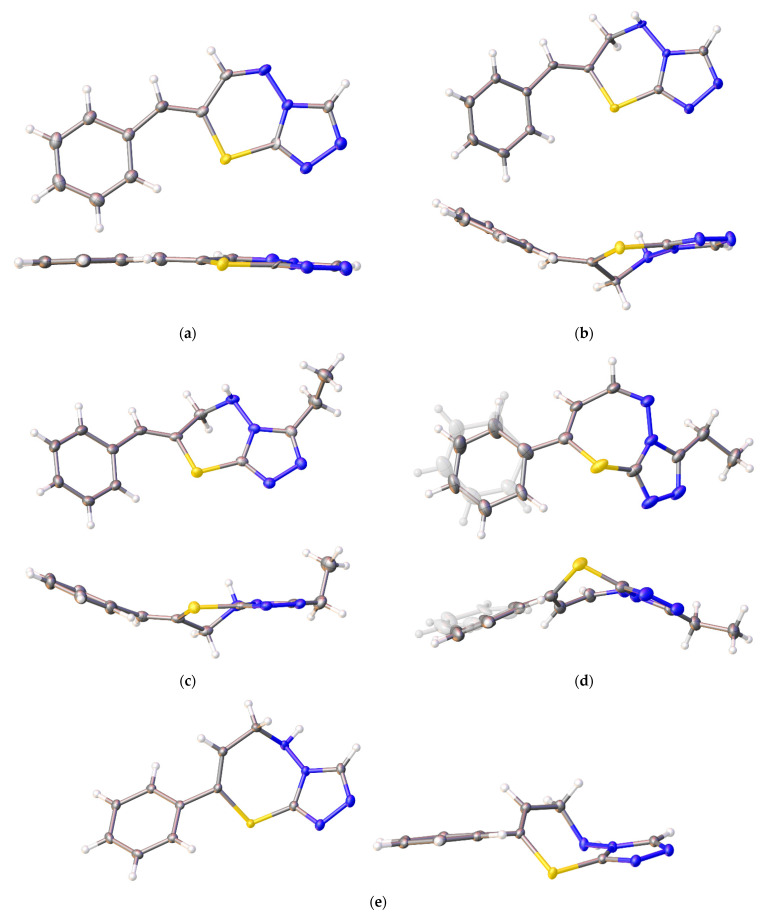
Structures of the compounds (**a**) **6a**; (**b**) **8a**; (**c**) **8c**; (**d**) **7c**; (**e**) **10a**.

Compounds **6** and **8** can exist as Z- or E-isomers. To determine the structure, we used X-ray diffraction analysis. By this method, the spatial structures of compounds **6a**, **7c**, **8a**, **8c** and **10a** were recorded in solid state. The data unambiguously interpreted the structure of compounds **6** and **8** as Z-isomers. The structures of compounds **6a**, **7c**, **8a**, **8c**, **10a** are shown in the Figure 8; Table 1 presents the crystal data for **6a**, **7c**, **8a**, **8c**, **10a**.

### 2.2. In Vitro Experiments

As shown earlier, heterocyclic nitrogen-containing compounds with exo- and endocyclic sulfur atoms (Figure 1) have attracted the attention of researchers as compounds with potential activity against various influenza viruses. These types include azomethines **1** and amines **2**, which are precursors for target [1,2,4]triazolo[3,4-*b*][1,3,4]thiadiazines **6** and **8**. Therefore, the need to study these, as well as target compounds, for activity against influenza virus A/Puerto Rico/8/34 (H1N1) was obvious. The results of our assessment of the cytotoxic and anti-viral activity of compounds are summarized in Table 2.

The compounds of the chemical library studied were of relatively low toxicity. Indeed, only one of 20 compounds tested (**7c**) had CC_50_ of one-digit micromoles. Nine compounds (**2a–b**, **6d**, **8a–d**, **10a–b**) did not demonstrate cytotoxicity, even at the highest concentration used (300 μg/mL, 1100–1300 μM).

Additionally, 10 out of 20 compounds (50%) (**1c**, **2a–c**, **6a**, **6d**, **8a**, **8c–d**, **10b**) tested against influenza virus demonstrated SI values of 11 and more. As follows from the presented results, compounds **1a–1c** were relatively toxic and of low activity. Although their IC_50_′s values were 11–25 μM, only one of them (compound **1c**) demonstrated a selectivity index higher than 10 due to relatively high toxicity. Hydrogenation of the azomethine fragment of aldimines **1** resulted in the appearance of secondary nitrogen (compounds **2**) and sharp increase of virus-suppressing properties. This was due to both a decrease of toxicity (**2a, 2b**) and, in the case of **2a**, an increase of activity in the single-digit micromolar range. Among all compounds tested, **2a** was the most promising (CC_50_ > 1300 μM, IC_50_ 4.3 μM, SI > 300). The nature of R was shown to be critical for anti-viral activity, as it decreased with R extension (H > Et > Me > n-Pr).

Cyclization of compounds **2a–2d** resulted in derivatives **6a–6d** with decreases in anti-viral efficacy. In contrast to **2a–2d,** n-propyl-bearing **6d** was of the highest activity (CC_50_ > 1100 μM, IC_50_ 34 μM, SI > 33) suggesting that the effect of R depended on the surrounding molecular context.

When comparing the analogs with secondary and tertiary nitrogen, it should be concluded that in the case of six-membered heterocycles (**6a–d** vs. **8a–d**), hydrogenation of the C=N bond of the azomethine moiety resulted in slight decreases of the compounds’ toxicity and did not substantially change their virus-inhibiting activity. In contrast, in the case of seven-membered rings (**7a–d** vs. **10a–d**), hydrogenation of the C=N bond of the azomethine moiety led to a sharp decrease of toxicity. Based on the obtained results, no conclusion could be made about the effect of the state of the atom N in the thiadiazine and thiadiazepine moieties on anti-viral activity; further studies would be needed to clarify the role of N state in biological activity and anti-viral perspectives of this class of compounds.

### 2.3. Activity Validation and Target Affinity Prediction

Docking results and values showed that the preferred interactant for our ligands was the M2 channel. All ligands showed better GlideScore values in the M2 proton channel (Table 3). Predicted affinity to neuraminidase for all ligands was significantly lower than control compound (oseltamivir).

In case of M2 channel IC_50_ values and scoring function, they did not correlate. We were unable to differentiate true/false positive results by GlideScore only (Table 3). Compounds with decreased activity level, such as **8b** or **10a**, had equal or better GlideScore values than active structures **1a** or **2a**. Thus, it was decided, for optimally clustered docking solutions, to calculate the free energy parameters and its components using the MM-GBSA method.

As a result, a correlation was established with the parameter of lipophilic interactions free energy and strained contacts energy with the M2 protein (channel). The first type of interaction is responsible by site-specific recognition and binding, the second type can describe interaction-induced strained contacts in protein-ligand complexes.

Thus, in a series of compounds **1a–c**, the growth of a substituent in triazole core (H→Et) led to increased activity levels due to stimulation of lipophilic interactions—and, as follows, lipophilic binding free energy was lower for **1c** (Table 3).

**Figure 9:**
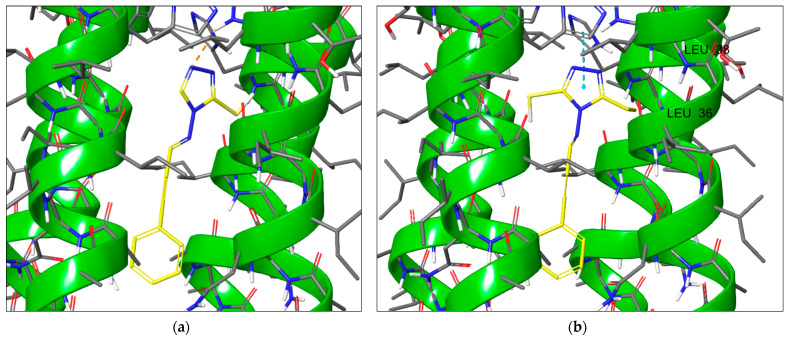
Binding poses of (**a**) **1a** and (**b**) **1c** in M2 channel active cavity; orange dashed line: strained protein-ligand contact, blue dashed line: pi-stacking interaction.

At the same time, it should be noted that in the case of **1c**, the scaffold was rotated in such a way that the ethyl substituent was oriented towards Leu36/38 (Figure 9), which was confirmed by a decrease in the free energy of lipophilic contacts. Increased strain energy was associated here with change in the torsion angle between triazole scaffold and imine moiety.

**Figure 10:**
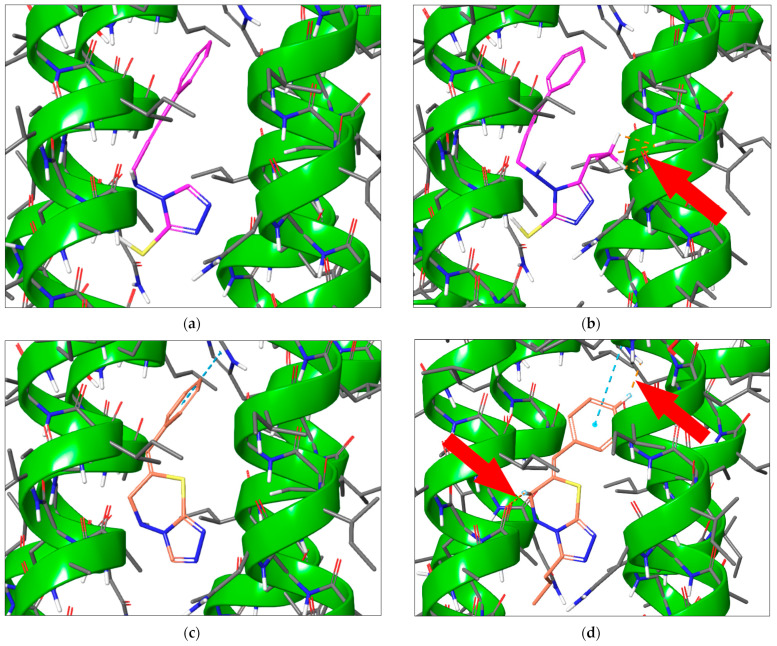
Binding poses of (**a**) **2a**; (**b**) **2d**; (**c**) **8a** and (**d**) **8d** in M2 channel active cavity, orange dashed line—strained protein-ligand contact (highlighted by red arrow), blue dashed line—pi-stacking interaction.

In series **2a–d**, we were able to observe serious changes in binding mode patterns. The extension of aliphatic substituent in triazole core possessed a negative role. For example, in case of compound **2d**, n-Pr substituent was oversized and led to the growth of strained contacts energy (Figure 10b, showed by an arrow, energy’s shown in Table 3). When passing to a series of compounds **8a–d**, the trend was preserved. With growth of the aliphatic substituent size, we observed activity diminution and increased strained contacts energy (Figure 10d). This phenomenon as associated with the influence of the lipophilic contact strength of compound **8d** with Ala27 in chains A–D of M2 channel. This interaction led to penetration of the ligand deeper into the proton channel and stimulated growth of strained contacts with other residues, which was energetically unfavorable.

All modeling procedures allowed us to conclude that the aliphatic substituent in the ligand structure was able to act as a steric key. This key can regulate activity profiles, dependent on scaffold structure, via balance between lipophilic contacts and strained contacts with M2 channel.

## 3. Materials and Methods

### 3.1. General Information

IR spectra were recorded on an IR Affinity-1 Fourier transform spectrometer for studies in the mid-IR range in KBr pellets. ^1^H and ^13^C NMR spectra (400 and 101 MHz, respectively) were acquired on a Bruker Avance III HD 400 NanoBay spectrometer in DMSO-d_6_, using the signals of the deuterated solvent DMSO-d6 as internal standard (the NMR spectra of the synthesized compounds are depicted in Supplementary Materials). High resolution mass spectra (electrospray ionization) were performed on a Shimadzu Nexera X2 LCMS-9030 liquid hybrid quadrupole time-of-flight mass spectrometer and Bruker micrOTOF. High performance liquid chromatography–high resolution mass spectrometry (HPLC-MS-HR) results were obtained on a Prominence LC-20 HPLC system (Shimadzu, Japan) in combination with an LTQ Orbitrap XL mass spectrometer (Thermo Fisher Scientific, Waltham, MA, USA) using electrospray ionization. Melting points were determined on a Büchi M-560 apparatus with a heating rate of 1 °C/min in the melting range. Monitoring of the reaction progress was done by TLC on Merck Kieselgel 60 F_254_ plates. Analytical grade solvents were used without additional purification.

### 3.2. Synthesis

**General procedure for the synthesis of 5-substituted 4-((3-phenylprop-2-yn-1-ylidene)amino)-4*H*-1,2,4-triazole-3-thiols (1a–c).**

First, 5-substituted 4-amino-4*H*-1,2,4-triazole-3-thiol **4a–c** (17.2 mmol) was dissolved under heating (60 °C) in isopropanol (20 mL). A catalytic amount of p-toluenesulfonic acid and 3-phenyl-2-propynal **3** (17.2 mmol) were added. Upon cooling to room temperature, a precipitate began to form. The reaction mixture was left overnight in the freezer, then filtered. The resulting compounds were characterized and used without further purification.

*4-((3-phenylprop-2-yn-1-ylidene)amino)-4H-1,2,4-triazole-3-thiol **1a**.* Yield 57%. Light yellow powder; mp 140–141 °C. IR (KBr), ν, cm^−1^: 1489 (Ph), 2201 (C≡C), 2349 (SH). ^1^H NMR (DMSO-d_6_), δ, ppm: 7.42–7.64 (3H, m, Ph), 7.68 (2H, d, Ph, *J =* 6.97 Hz), 8.91 (1H, s, CH=N), 9.26 (1H, s, CH_triazole_), 14.04 (1H, br. s, SH). ^13^C NMR (DMSO-d_6_), δ, ppm: 83.97, 99.69, 120.36, 129.57, 131.32, 132.70, 138.53 (CH=N), 144.58 (CH_triazole_), 163.48. HRMS (ESI), *m*/*z*: 251.0361 [M + Na]^+^ (calculated for C_11_H_8_N_4_SNa: 251.0362).

*5-methyl-4-((3-phenylprop-2-yn-1-ylidene)amino)-4H-1,2,4-triazole-3-thiol **1b**.* Yield 55%. Light yellow powder; mp 161–162 °C. IR (KBr), ν, cm^−1^: 1489 (Ph), 2204 (C≡C), 2358 (SH). ^1^H NMR (DMSO-d_6_), δ, ppm: 2.33 (3H, s, CH_3_), 7.47–7.57 (3H, m, Ph), 7.69 (2H, d, Ph, *J =* 6.97 Hz), 10.05 (1H, s, CH=N), 13.86 (1H, br. s, SH). ^13^C NMR (DMSO-d_6_), δ, ppm: 11.26 (CH_3_), 83.94, 100.54, 120.37, 129.53, 131.29, 132.71, 146.44 (CH=N), 149.10, 161.69. HRMS (ESI), *m*/*z*: 243.0699 [M + H]^+^ (calculated for C_12_H_11_N_4_S: 243.0699).

*5-ethyl-4-((3-phenylprop-2-yn-1-ylidene)amino)-4H-1,2,4-triazole-3-thiol **1c**.* Yield 50%. Light yellow powder; mp 115–116 °C. IR (KBr), ν, cm^−1^: 1490 (Ph), 2201 (C≡C), 2359 (SH). ^1^H NMR (DMSO-d_6_), δ, ppm: 1.21 (3H, t, CH_3_, *J =* 7.46 Hz), 2.71 (2H, q, CH_2_, *J =* 7.46 Hz), 7.37–7.65 (3H, m, Ph), 7.69 (2H, d, Ph, *J =* 6.97 Hz), 10.01 (1H, s, CH=N), 13.89 (1H, br. s, SH). ^13^C NMR (DMSO-d_6_), δ, ppm: 10.48 (CH_3_), 18.64 (CH_2_), 83.94, 100.57, 120.36, 129.54, 131.32, 132.73, 146.90 (CH=N), 152.87, 161.82. HRMS (ESI), *m*/*z*: 257.0856 [M + H]^+^ (calculated for C_13_H_13_N_4_S: 257.0855).

**Procedure for the synthesis of 5-propyl-4-((3-phenylprop-2-yn-1-ylidene)amino)-4*H*-1,2,4-triazole-3-thiol (1d).**

First, 4-amino-5-propyl-4*H*-1,2,4-triazole-3-thiol **4d** (0.76 mmol) was dissolved in methanol (10 mL) under heating (60 °C). Then, 3-phenyl-2-propynal **3** (0.76 mmol) and sulfuric acid (0.91 mmol) were added. The completion of the reaction was monitored by TLC (ethyl acetate/petroleum ether, 1:3 by volume). The resulting substance was used without further isolation from the reaction mixture.

**General procedure for the synthesis of 5-substituted 4-((3-phenylprop-2-yn-1-yl)amino)-4*H*-1,2,4-triazole-3-thiols (2a–c).**

In a flat-bottomed flask equipped with a magnet stirrer and condenser, 5-substituted 4-((3-phenylprop-2-yn-1-ylidene)amino)-4*H*-1,2,4-triazole-3-thiol **1a–d** (9.8 mmol) was dissolved in methanol (50 mL). Sodium borohydride (14.8 mmol) was added in portions to the resulting solution, avoiding substantial foaming. Then, the reaction mixture was refluxed for 5 min, cooled, and diluted with 180 mL of cold water. The cooled solution was acidified with hydrochloric acid to pH 2. The precipitate formed was filtered off and recrystallized from aqueous ethanol.

*4-((3-phenylprop-2-yn-1-yl)amino)-4H-1,2,4-triazole-3-thiol **2a**.* Yield 92%. Colorless powder; mp 156–157 °C. IR (KBr), ν, cm^−1^: 1488 (Ph), 2334 (C≡C), 2360 (SH), 3235 (NH). ^1^H NMR (DMSO-d_6_), δ, ppm: 4.21 (2H, s, CH_2_), 6.82 (1H, br. s, NH-CH2), 7.26–7.51 (5H, m, Ph), 8.54 (1H, s, CH_triazole_), 13.77 (1H, br. s, SH). ^13^C NMR (DMSO-d_6_), δ, ppm: 39.64 (CH_2_), 85.08, 85.56, 122.42, 129.11, 129.16, 131.78, 143.18 (CH_triazole_), 165.42. HRMS (ESI), *m*/*z*: 231.0699 [M + H]^+^ (calculated for C_11_H_11_N_4_S: 231.0699).

*5-methyl-4-((3-phenylprop-2-yn-1-yl)amino)-4H-1,2,4-triazole-3-thiol **2b**.* Yield 92%. Colorless powder; mp 145–146 °C. IR (KBr), ν, cm^−1^: 1491 (Ph), 2339 (C≡C), 2360 (SH), 3215 (NH). ^1^H NMR (DMSO-d_6_), δ, ppm: 2.35 (3H, s, CH_3_), 4.28 (2H, d, CH_2_, *J =* 3.42 Hz), 6.69 (1H, t, NH, *J =* 3.42 Hz), 7.38 (5H, s, Ph), 13.58 (1H, br. s, SH). ^13^C NMR (DMSO-d_6_), δ, ppm: 11.04 (CH_3_), 38.90 (CH_2_), 84.50, 85.87, 122.33, 129.22, 131.67, 150.80, 165.80. HRMS (ESI), *m*/*z*: 267.0675 [M + Na]^+^ (calculated for C_12_H_12_N_4_SNa: 267.0675).

*5-ethyl-4-((3-phenylprop-2-yn-1-yl)amino)-4H-1,2,4-triazole-3-thiol **2c**.* Yield 90%. Colorless powder; mp 111–112 °C. IR (KBr), ν, cm^−1^: 1489 (Ph), 2337 (C≡C), 2360 (SH), 3244 (NH). ^1^H NMR (DMSO-d_6_), δ, ppm: 1.19 (3H, t, CH_3_, *J =* 7.53 Hz), 2.78 (2H, q, CH_2_, *J =* 7.53 Hz), 4.30 (2H, d, CH_2_-NH, *J =* 3.21 Hz), 6.62 (1H, t, NH, *J =* 3.21 Hz), 7.28–7.46 (5H, m, Ph), 13.56 (1H, s, SH). ^13^C NMR (DMSO-d_6_), δ, ppm: 10.76 (CH_3_), 18.44 (CH_2_), 38.79 (CH_2_-NH), 84.51, 85.85, 122.33, 129.21, 131.67, 154.86, 165.99. HRMS (ESI), *m*/*z*: 259.1014 [M + H]^+^ (calculated for C_13_H_15_N_4_S: 259.1012).

**Procedure for the synthesis of 5-propyl-4-((3-phenylprop-2-yn-1-yl)amino)-4*H*-1,2,4-triazole-3-thiol (2d).**

To the solution of previously obtained 5-propyl-4-((3-phenylprop-2-yn-1-ylidene)amino)-4*H*-1,2,4-triazole-3-thiol **1d** in methanol, sodium borohydride (1.5 mmol) was added in portions, avoiding substantial foaming. Then, the reaction mixture was refluxed for 5 min, cooled, and diluted with 50 mL of cold water. The cooled solution was acidified with hydrochloric acid to pH 2. The precipitate that formed was filtered off and recrystallized from aqueous ethanol. The overall yield for the two stages was 40%. Colorless powder; mp 113–114 °C. IR (KBr), ν, cm^−1^: 1490 (Ph), 2336 (C≡C), 2360 (SH), 3247 (NH). ^1^H NMR (DMSO-d_6_), δ, ppm: 0.89 (3H, t, CH_3_, *J =* 7.303 Hz), 1.73–1.57 (2H, m, CH_2_), 2.73 (2H, t, CH_2_, *J =* 7.303 Hz), 4.30 (2H, br. s, CH_2_-NH), 6.66 (1H, br. s, NH), 7.37 (5H, m, Ph), 13.59 (1H, br. s, SH). ^13^C NMR (DMSO-d_6_), δ, ppm: 13.95 (CH_3_), 19.52 (CH_2_), 26.59 (CH_2_), 38.81 (CH_2_-NH), 84.51, 85.90, 122.35, 129.19, 131.68, 153.70, 165.90. HRMS (ESI), *m*/*z*: 273.1173 [M + H]^+^ (calculated for C_14_H_17_N_4_S: 273.1168).

**General procedure for the synthesis of 3-substituted (Z)-7-benzylidene-6,7-dihydro-5*H*-[1,2,4]triazolo[3,4-*b*][1,3,4]thiadiazines (8a–d) by cyclization 5-substituted 4-((3-phenylprop-2-yn-1-yl)amino)-4*H*-1,2,4-triazole-3-thiols (2a–d).**

In a flat-bottomed flask, 5-substituted 4-((3-phenylprop-2-yn-1-yl)amino)-4*H*-1,2,4-triazole-3-thiol **2a–c** (9.1 mmol) was dissolved in 10 mL of DMF. Two drops of 50% aqueous KOH solution were added as a catalyst. Reaction mixture was heated for 2.5 h at 100 °C. The reaction progress was monitored by TLC (ethyl acetate/petroleum ether, 1:2 by volume). Then, the reaction mixture was diluted with 40 mL of cold water. The resulting suspension was cooled to +5 °C, the precipitate was filtered off and recrystallized from aqueous ethanol.

*(Z)-7-benzylidene-6,7-dihydro-5H-[1,2,4]triazolo[3,4-b][1,3,4]thiadiazine **8a**.* Yield 88%. Colorless crystals; mp 194–195 °C. IR (KBr), ν, cm^−1^: 1489 (Ph), 3286 (NH). ^1^H NMR (DMSO-d_6_), δ, ppm: 4.02–4.17 (2H, d, CH_2_, *J =* 6.85 Hz), 6.91 (1H, s, CH_benzylidene_), 7.06 (1H, t, NH, *J =* 6.85 Hz), 7.26–7.54 (5H, m, Ph), 8.69 (1H, s, CH_triazole_). ^13^C NMR (DMSO-d_6_), δ, ppm: 53.15 (CH_2_), 124.66, 126.48, 128.31, 128.93, 129.30, 135.22, 141.27, 143.96 (CH_triazole_). HRMS (ESI), *m*/*z*: 231.0709 [M + H]^+^ (calculated for C_11_H_11_N_4_S: 231.0699).

*(Z)-7-benzylidene-3-methyl-6,7-dihydro-5H-[1,2,4]triazolo[3,4-b][1,3,4]thiadiazine **8b**.* Yield 80%. Colorless crystals; mp 202–203 °C. IR (KBr), ν, cm^−1^: 1490 (Ph), 3162 (NH). ^1^H NMR (DMSO-d_6_), δ, ppm: 2.29 (3H, s, CH_3_), 4.08 (2H, d, CH_2_, *J =* 6.24 Hz), 6.82 (1H, br. s, NH), 6.90 (1H, s, CH), 7.34 (1H, br. s, Ph), 7.46 (4H, br. s, Ph). ^13^C NMR (DMSO-d_6_), δ, ppm: 9.67 (CH_3_), 53.34 (CH_2_), 124.33, 126.17 (CH-Ph), 128.25, 128.93, 129.28, 135.31, 140.79, 151.14. HRMS (ESI), *m*/*z*: 245.0859 [M + H]^+^ (calculated for C_12_H_13_N_4_S: 245.0855).

*(Z)-7-benzylidene-3-ethyl-6,7-dihydro-5H-[1,2,4]triazolo[3,4-b][1,3,4]thiadiazine **8c**.* Yield 82%. Colorless crystals; mp 208–209 °C. IR (KBr), ν, cm^−1^: 1490 (Ph), 3149 (NH). ^1^H NMR (DMSO-d_6_), δ, ppm: 1.22 (3H, t, CH_3_, *J =* 7.52 Hz), 2.68 (2H, q, CH_2_, *J =* 7.58 Hz), 4.08 (2H, d, CH_2_-NH, *J =* 6.97 Hz), 6.85 (1H, t, NH, *J =* 6.91 Hz), 6.90 (1H, s, CH), 7.27–7.40 (1H, m, Ph), 7.42–7.55 (4H, m, Ph). ^13^C NMR (DMSO-d_6_), δ, ppm: 11.93 (CH_3_), 17.48 (CH_2_), 53.35 (CH_2_-NH), 124.49, 126.11 (CH-Ph), 128.23, 128.92, 129.28, 135.32, 140.93, 155.40. HRMS (ESI), *m*/*z*: 259.1005 [M + H]^+^ (calculated for C_13_H_15_N_4_S: 259.1012).

*(Z)-7-benzylidene-3-propyl-6,7-dihydro-5H-[1,2,4]triazolo[3,4-b][1,3,4]thiadiazine **8d**.* Yield 75%. Colorless crystals; mp 196–198 °C. IR (KBr), ν, cm^−1^: 1489 (Ph), 3166 (NH). ^1^H NMR (DMSO-d_6_), δ, ppm: 0.93 (3H, t, CH_3_, *J =* 7.41 Hz), 1.66 (2H, m, CH_2_, *J =* 7.41 Hz), 2.64 (2H, t, CH_2_, *J =* 7.46 Hz), 4.07 (2H, d, CH_2_-NH, *J =* 6.72 Hz), 6.85 (1H, t, *J =* 6.97 Hz), 6.89 (1H, s, CH-Ph), 7.30–7.40 (1H, m, Ph), 7.42–7.50 (4H, m, Ph). ^13^C NMR (DMSO-d_6_), δ, ppm: 14.04 (CH_3_), 20.62 (CH_2_), 25.67 (CH_2_), 53.42 (CH_2_-NH), 124.53, 126.06, 128.23, 128.92, 129.28, 135.32, 140.89, 154.26. HRMS (ESI), *m*/*z*: 273.1164 [M + H]^+^ (calculated for C_14_H_17_N_4_S: 273.1168).

**General procedure for the synthesis of 3-substituted (Z)-7-benzylidene-7*H*-[1,2,4]triazolo[3,4-*b*][1,3,4]thiadiazines (6a–d) by oxidation of 3-substituted (Z)-7-benzylidene-6,7-dihydro-5*H*-[1,2,4]triazolo[3,4-*b*][1,3,4]thiadiazines (8a–d).**

In a flat-bottomed flask equipped with a magnetic stirrer and condenser, 3-substituted (Z)-7-benzylidene-6,7-dihydro-5*H*-[1,2,4]triazolo[3,4-*b*][1,3,4]thiadiazine **8a–d** (0.6 mmol) was dissolved in acetonitrile (50 mL) and active manganese dioxide was added (9 mmol). The reaction mixture was refluxed with vigorous stirring for 5–6 h. The completion of the reaction was monitored by TLC in pure ethyl acetate. Next, manganese dioxide was filtered off and 100 mL of water was added to the filtrate. The precipitate that formed was filtered off without need of further purification.

*(Z)-7-benzylidene-7H-[1,2,4]triazolo[3,4-b][1,3,4]thiadiazine **6a**.* Yield 88%. Colorless crystals; mp 226–227 °C. IR (KBr), ν, cm^−1^: 1489 (Ph), 1580 (C=N). ^1^H NMR (DMSO-d_6_), δ, ppm: 7.37–7.67 (6H, m, benzylidene), 8.13 (1H, s, CH=N), 9.10 (1H, s, CH_triazole_). ^13^C NMR (DMSO-d_6_), δ, ppm: 116.90, 129.67, 129.91, 130.38, 133.97, 134.66, 136.78, 143.64, 149.03. HRMS (ESI), *m*/*z*: 251.0360 [M + Na]^+^ (calculated for C_11_H_8_N_4_SNa: 251.0362).

*(Z)-7-benzylidene-3-methyl-7H-[1,2,4]triazolo[3,4-b][1,3,4]thiadiazine **6b**.* Yield 91%. Colorless crystals; mp 201–202 °C. IR (KBr), ν, cm^−1^: 1489 (Ph), 1582 (C=N). ^1^H NMR (DMSO-d_6_), δ, ppm: 2.44 (3H, s, CH_3_), 7.43–7.51 (2H, m), 7.52–7.64 (4H, m), 8.12 (1H, s, CH=N). ^13^C NMR (DMSO-d_6_), δ, ppm: 9.82 (CH_3_), 116.36, 129.67, 129.92, 130.32, 134.06, 134.32, 136.38 (CH-Ph), 148.17 (CH=N), 150.91. HRMS (ESI), *m*/*z*: 265.0516 [M + Na]^+^ (calculated for C_12_H_10_N_4_SNa: 265.0518).

*(Z)-7-benzylidene-3-ethyl-7H-[1,2,4]triazolo[3,4-b][1,3,4]thiadiazine **6c**.* Yield 88%. Colorless crystals; mp 165–166 °C. IR (KBr), ν, cm^−1^: 1490 (Ph), 1581 (C=N). ^1^H NMR (DMSO-d_6_), δ, ppm: 1.27 (3H, t, CH_3_, *J =* 7.40 Hz), 2.84 (2H, q, CH_2_, *J =* 7.34 Hz), 7.42–7.72 (6H, m, benzylidene), 8.11 (1H, s, CH=N). ^13^C NMR (DMSO-d_6_), δ, ppm: 11.59 (CH_3_), 17.62 (CH_2_), 116.40, 129.65, 129.90, 130.30, 134.06, 134.45, 136.39, 148.11, 154.86. HRMS (ESI), *m*/*z*: 257.0855 [M + H]^+^ (calculated for C_13_H_13_N_4_S: 257.0855).

*(Z)-7-benzylidene-3-propyl-7H-[1,2,4]triazolo[3,4-b][1,3,4]thiadiazine **6d**.* Yield 85%. Colorless crystals; mp 163–164 °C. IR (KBr), ν, cm^−1^: 1491 (Ph), 1581 (C=N). ^1^H NMR (DMSO-d_6_), δ, ppm: 0.96 (3H, t, CH_3_, *J =* 7.40 Hz), 1.63–1.83 (2H, m, CH_2_), 2.80 (2H, t, CH_2_, *J =* 7.46 Hz), 7.45–7.63 (6H, m, benzylidene), 8.11 (1H, s, CH=N). ^13^C NMR (DMSO-d_6_), δ, ppm: 13.98 (CH_3_), 20.43 (CH_2_), 25.69 (CH_2_), 116.39, 129.64, 129.90, 130.30, 134.07, 134.37, 136.40, 148.14, 153.73. HRMS (ESI), *m*/*z*: 293.0829 [M + Na]^+^ (calculated for C_14_H_14_N_4_SNa: 293.0831).

**General procedure for the synthesis of (Z)-7-benzylidene-7*H*-[1,2,4]triazolo[3,4-*b*][1,3,4]thiadiazines (6a–c) from** **α-bromocinnamic aldehyde.**

3-substituted 4-amino-4*H*-1,2,4-triazole-3-thiol **4a–c** (9.2 mmol), α-bromocinnamic aldehyde (13.8 mmol), and triethylamine (18.4 mmol) in 40 mL of ethanol were added to a single-necked flask equipped with magnetic stirrer and condenser. The reaction mixture was refluxed for one and a half hour. The reaction progress was monitored by TLC (ethyl acetate/petroleum ether, 1:1 by volume). The reaction mixture was cooled in the freezer for two hours. The resulting white precipitate was filtered off and recrystallized from aqueous ethanol. Yields: 59% (**6a**), 46% (**6b**), 52% (**6c**), 40% (**6d**).

**General procedure for the synthesis of 3-substituted (Z)-7-benzylidene-6,7-dihydro-5*H*-[1,2,4]triazolo[3,4-*b*][1,3,4]thiadiazines (8a–d) by hydrogenation 3-substituted (Z)-7-benzylidene-7*H*-[1,2,4]triazolo[3,4-*b*][1,3,4]thiadiazines (6a–d).**

In a round-bottomed flask equipped with a magnetic stirrer and a condenser, 3-substituted (Z)-7-benzylidene-7*H*-[1,2,4]triazolo[3,4-*b*][1,3,4]thiadiazines **6a–d** (4.2 mmol) was dissolved in 20 mL of methanol. To the resulting solution, sodium borohydride (6.4 mmol) was added in portions, avoiding substantial foaming. Then, the reaction mixture was refluxed for 5 min, cooled, and diluted with 50 mL of cold water. The precipitate that formed was filtered off and recrystallized from aqueous ethanol. Yields: 89% (**8a**), 88% (**8b**), 88% (**8c**), 85% (**8d**).

**General procedure for the synthesis of 3-substituted 8-phenyl-[1,2,4]triazolo[3,4-*b*][1,3,4]thiadiazepines (7a–d).**

Tert-butylamine (0.01 mol) and 3-phenyl-2-propynal **3** (0.01 mol) were added to methanol (20 mL). To the solution of the resulting aldimine **9** 5-substituted 4-amino-4*H*-1,2,4-triazole-3-thiol **4a–c** (0.01 mol) was added with stirring in small portions over a period of several minutes. The reaction mixture slightly warmed up, with little darkening. The reaction progress was monitored by TLC (ethyl acetate/petroleum ether, 1:1 by volume). The mixture was kept for 1 h at room temperature, after which the solvent was evaporated and the resulting compound was recrystallized from aqueous ethanol.

*8-phenyl-[1,2,4]triazolo[3,4-b][1,3,4]thiadiazepine **7a.*** Yield 85%. Colorless crystals; mp 196–197 °C. ^1^H NMR (DMSO-d_6_), δ, ppm: 6.91 (1H, d, CH, J = 4.03 Hz), 7.46–7.56 (3H, m, Ph), 7.81 (2H, dd, Ph, *J =* 7.40, 1.77 Hz), 8.22 (1H, d, CH=N, *J =* 4.03 Hz), 9.04 (1H, s, CH_triazole_). ^13^C NMR (DMSO-d_6_), δ, ppm: 124.72 (CH=C-S), 127.80 (CH_Ph_), 129.59 (CH_Ph_), 131.55 (CH_Ph_), 136.22, 144.48, 145.88 (CH_triazole_), 146.90, 158.30 (CH=N). LCMS (ESI), *m*/*z*: 229.0547 [M + H]^+^ (calculated for C_11_H_9_N_4_S: 229.0542).

*3-methyl-8-phenyl-[1,2,4]triazolo[3,4-b][1,3,4]thiadiazepine **7b.*** Yield 87%. yellow crystals; mp 212 °C. ^1^H NMR (DMSO-d_6_), δ, ppm: 2.40 (3H, s, CH_3_), 6.90 (1H, d, CH, *J =* 4.03 Hz), 7.46–7.57 (3H, m, Ph), 7.76–7.86 (2H, m, Ph), 8.21 (1H, d, CH=N, *J =* 4.03 Hz). ^13^C NMR (DMSO-d_6_), δ, ppm: 10.69 (CH_3_), 124.71 (CH=C-S), 127.74 (CH_Ph_), 129.60 (CH_Ph_), 131.48, 136.30, 144.44, 146.65, 153.38, 157.50 (CH=N). HPLC-MS-HR (ESI), *m*/*z*: 243.0701[M + H]^+^ (calculated for C_12_H_11_N_4_S: 243.0698).

*3-ethyl-8-phenyl-[1,2,4]triazolo[3,4-b][1,3,4]thiadiazepine **7c**.* Yield 83%. Yellow crystals; mp 155–156 °C. ^1^H NMR (DMSO-d_6_), δ, ppm: 1.24 (3H, t, CH_3_, *J =* 7.52 Hz), 2.79 (2H, q, CH_2_, *J =* 7.54 Hz), 6.90 (1H, d, CH, *J =* 4.03 Hz), 7.48–7.59 (3H, m, Ph), 7.77–7.86 (2H, m, Ph), 8.21 (1H, d, CH=N, *J =* 4.03 Hz). ^13^C NMR (DMSO-d_6_), δ, ppm: 11.26 (CH3), 18.31 (CH2), 124.73 (CH=C-S), 127.74 (CH_Ph_), 129.59 (CH_Ph_), 131.48 (CH_Ph_), 136.27, 144.52, 146.71, 157.23, 157.50 (CH=N). LCMS (ESI), *m*/*z*: 257.0861 [M + H]^+^ (calculated for C_13_H_13_N_4_S: 257.0855).

*8-phenyl-3-propyl-[1,2,4]triazolo[3,4-b][1,3,4]thiadiazepine **7d**.* Yield 70%. yellow crystals; mp 88–89 °C. ^1^H NMR (DMSO-d_6_), δ, ppm: 0.94 (3H, t, CH_3_, *J =* 7.34 Hz), 1.70 (2H, sxt, CH_2_, *J =* 7.41 Hz), 2.75 (2H, t, CH_2_, *J =* 7.46 Hz), 6.91 (1H, d, CH, *J =* 4.03 Hz), 7.46–7.58 (3H, m), 7.77–7.86 (2H, m), 8.22 (1H, d, CH=N, *J =* 4.03 Hz). ^13^C NMR (DMSO-d_6_), δ, ppm: 14.00 (CH_3_), 20.13 (CH_2_), 26.41 (CH_2_), 124.65 (CH=C-S), 127.76 (CH_Ph_), 129.37 (CH_Ph_), 131.51 (CH_Ph_), 136.22, 144.46, 146.81, 156.15, 157.61 (CH=N). HPLC-MS-HR (ESI), *m*/*z*: 271.1021 [M + H]^+^ (calculated for C_14_H_15_N_4_S: 271.1011).

**General procedure for the synthesis of 3-substituted 8-phenyl-5,6-dihydro-[1,2,4]triazolo[3,4-*b*][1,3,4]thiadiazepines (10a–d).**

In a flat-bottomed flask equipped with a magnetic stirrer and a condenser, 3-substituted 8-phenyl-[1,2,4]triazolo[3,4-*b*][1,3,4]thiadiazepine **7a–d** (4.2 mmol) was dissolved in 20 mL of methanol. To the resulting solution, sodium borohydride (6.4 mmol) was added in portions, avoiding substantial foaming. Then, the reaction mixture was refluxed for 5 min, cooled and diluted with 50 mL of cold water. The precipitate that formed was filtered off and recrystallized from aqueous ethanol.

*8-phenyl-5,6-dihydro-[1,2,4]triazolo[3,4-b][1,3,4]thiadiazepine **10a**.* Yield 92%. Colorless crystals; mp 173–174 °C. ^1^H NMR (DMSO-d_6_), δ, ppm: 3.96 (2H, t, CH_2_, J = 4.58 Hz), 6.05 (1H, t, CH-CH_2_, *J =* 3.73 Hz), 7.37–7.49 (3H, m), 7.51–7.59 (3H, m), 8.74 (1H, s, CH_triazole_). ^13^C NMR (DMSO-d_6_), δ, ppm: 50.80 (CH_2_), 127.64 (CH_Ph_), 128.36 (CH_Ph_), 128.82 (CH=C-S), 129.30 (CH_Ph_), 129.39, 139.69, 145.69 (CH_triazole_), 149.86. LCMS (ESI), *m*/*z*: 231.0704 [M + H]^+^ (calculated for C_11_H_11_N_4_S: 231.0699).

*3-methyl-8-phenyl-5,6-dihydro-[1,2,4]triazolo[3,4-b][1,3,4]thiadiazepine **10b**.* Yield 93%. Colorless crystals; mp 175–176 °C. ^1^H NMR (DMSO-d_6_), δ, ppm: 2.37 (3H, s, CH_3_), 3.95 (2H, s, CH_2_), 6.02 (1H, s, CH-CH_2_), 7.24–7.49 (4H, m), 7.49–7.78 (2H, m). ^13^C NMR (DMSO-d_6_), δ, ppm: 9.94 (CH_3_), 50.25 (CH_2_), 127.58 (CH_Ph_), 128.62 (CH-CH_2_), 129.01 (CH_Ph_), 129.29 (CH_Ph_), 139.79, 149.72, 153.47. HPLC-MS-HR (ESI), *m*/*z*: 245.0857 [M + H]^+^ (calculated for C_12_H_13_N_4_S: 245.0855).

*3-ethyl-8-phenyl-5,6-dihydro-[1,2,4]triazolo[3,4-b][1,3,4]thiadiazepine **10c**.* Yield 89%. Colorless crystals; mp 149–150 °C. ^1^H NMR (DMSO-d_6_), δ, ppm: 1.25 (3H, t, CH_3,_ *J =* 7.58 Hz), 2.75 (2H, q, CH_2_, *J =* 7.54 Hz), 3.94 (2H, dd, CH_2_-NH, *J =* 5.87, 3.91 Hz), 6.01 (1H, t, CH-CH_2_, *J =* 3.73 Hz), 7.36 (1H, t, *J =* 6.17 Hz), 7.38–7.46 (3H, m), 7.55 (2H, d, *J =* 6.85 Hz). ^13^C NMR (DMSO-d_6_), δ, ppm: 12.18 (CH_3_), 17.66 (CH_2_), 50.41 (CH_2_-NH), 127.57, 128.52, 128.98 (CH-CH_2_), 129.28, 129.31, 139.76, 149.94, 157.61. LCMS (ESI), *m*/*z*: 259.1018 [M + H]^+^ (calculated for C_13_H_15_N_4_S: 259.1012).

*8-phenyl-3-propyl-5,6-dihydro-[1,2,4]triazolo[3,4-b][1,3,4]thiadiazepine **10d**.* Yield 90%. Colorless crystals; mp 103–104 °C. ^1^H NMR (DMSO-d_6_), δ, ppm: 0.96 (3H, t, CH_3_, *J =* 7.40 Hz), 1.69 (2H, sxt, CH_2_, *J =* 7.43 Hz), 2.71 (2H, t, CH_2_, *J =* 7.52 Hz), 3.93 (2H, dd, CH_2_-NH, *J =* 6.11, 3.91 Hz), 6.01 (1H, t, CH-CH_2_, *J =* 3.85 Hz), 7.33–7.48 (4H, m), 7.51–7.60 (2H, m). ^13^C NMR (DMSO-d_6_), δ, ppm: 14.13 (CH_3_), 20.93 (CH_2_), 25.82 (CH_2_), 50.45 (CH_2_-NH), 127.58, 128.46, 128.93 (CH-CH_2_), 129.30, 129.34, 139.72, 149.86, 156.48. HPLC-MS-HR (ESI), *m*/*z*: 273.1174 [M + H]^+^ (calculated for C_14_H_17_N_4_S: 273.1168).

### 3.3. X-ray Diffraction

The single crystal X-ray diffraction data were collected using an Agilent Technologies SuperNova(s15383: **10a**), Rigaku XtaLAB SuperNova (s15380: **7c**), Rigaku «XtaLAB Synergy-S» (s15979: **6a**) and Agilent Technologies «Xcalibur» (e15: **8a**, e16: **8c**) diffractometer. The temperature was kept at 100 K throughout the experiment. Empirical absorption correction was applied in CrysAlisPro (Agilent Technologies, 2014) program complex using spherical harmonics, implemented in SCALE3 ABSPACK scaling algorithm. The structures were solved by SHELXT program [38], using the least squares minimization in anisotropic (for non-hydrogen atoms) approximation, and refined with the SHELXL package [39] incorporated in the Olex2 program package [40]. The hydrogen atoms were introduced to the geometrically calculated positions and refined by attachment to the corresponding parent atoms. The disorder within one independent half of molecule s15380 was modeled by the free-populated superposition of two sets of the benzene ring coordinates, corresponding to slightly different positions of the ring in space. Crystallographic data for the studied samples were deposited at Cambridge Crystallographic Data center (**6a**: CCDC 2212189; **7c**: CCDC—2212192; **8a**: CCDC 1813554; **8c**: CCDC 1813556 and **10a**: CCDC 2212194).

### 3.4. Molecular Docking

*Protein structure preparation.* We observed two main targets of our ligands: M2 channel of influenza virus and neuraminidase N1 of influenza virus. Neuraminidase structure was downloaded from RCSB Protein Data bank [41]. PDB structure id 3TI6 (A/California/04/2009(H1N1)), protein model for virus strain A/Puerto Rico/8/1934 H1N1 was not presented, so we used the closest by sequence/structure model, with addition of point mutations in sequence, equalizing it to neuraminidase from strain A/Puerto Rico/8/1934 H1N1.

In the case of the M2 channel, the protein structure for A/Puerto Rico/8/1934 H1N1 strain was not available in RCSB PDB. However, the protein sequence could be found in the Uniprot database: Matrix protein 2 Influenza A virus (strain A/Puerto Rico/8/1934 H1N1), accession code P06821. M2 protein structure was built with use of the homology modeling method. The following protein models, obtained from RCSB Protein Data Bank, were used as templates: 2KIH, 2N70, 4N8C, 6SOY (H3N2), 5JOO, 5TTC, 5UM1 (H1N1). Resulting chimeric model geometry was optimized with use of the Prime module from Schrodinger Suite 2022-2. The model quality was controlled via Ramachandran plot (Figure S47).

All models needed to be preprocessed using Schrodinger Protein PrepWizard [42]. On this step, we excluded typical structure errors, such as invalid protonation state, missing amino acid sidechains, missing loops, and incorrect bond orders.

For all observed protein models, protonation states, hydrogen bonds and charges calculated for pH equated to 7,4 using the PROPKA method [43].

Protein structure was refined using restrained minimization (needed for elimination of local strained contacts in protein). All manipulations with proteins and ligands were carried out in OPLS4 forcefield [44], used in Schrodinger Suite 2022-2.

*Ligand structure preparation.* Three-dimensional structure of ligands, used for calculations, generated in the same forcefield—OPLS4 [44].

*Molecular docking: GridBox building.* Before the molecular docking, a grid box was built, covering the M2 channel and neuraminidase active pockets. The size of the gridbox was chosen in accordance with the reference ligand size, which was present in model (for neuraminidase: oseltamivir; for M2 channel: rimantadine), and is equal to 10 Å (one side of cube). In M2 channel cavity, the gridbox was placed on the centroid of selected residues: Gly34 in chains A-D, cube side size was 10 Å. VdW scaling was 1.0 Å, partial charge cutoff—0.25 Å. No constraints were applied for all observed proteins. GlideGrid program [45] from Schrodinger Suite 2022-2 was used for calculation.

*Molecular docking: docking procedure.* The prepared ligands, docked into previously generated GridBoxes of our proteins, were neuraminidase and M2 channel. For all the selected ligands, the standard precision mode with enhanced flexible sampling was used. For each ligand state, a penalty value (if ligand was ionizable) was added, generating 15 docking solutions for each structure, Strain correction terms were added (necessary for ligand torsions’ parametrization before final scoring). The resulting docking solutions were clustered. The best-fitting solution was compared to the reference structure binding mode by Glidescore/Emodel values and its components.

*MM-GBSA.* Binding free energy and its components, such as Gibb’s free energy, strain energy, and other parameters, were calculated for all the best-fitting docking solutions using the MM-GBSA method [46]. Solvation model was VSGB, protein flexibility radius is 6 Å around ligand.

### 3.5. The Study of the Biological Activity

The cytotoxic properties of the compounds were investigated in a separate series of experiments. A series of threefold dilutions (300–3.7 µg/mL) were prepared from the test compounds, after which they were added to the wells of plates with a monolayer of MDCK cells (ATCC CCL-34). The plates were incubated for 72 h at 36 °C. The cell viability was analyzed using the methyltetrazolium assay; a solution of methyltetrazolium bromide was introduced into the wells, which, by the action of mitochondrial enzymes, was converted into an insoluble violet formazan derivative [47]. The plates with cells were incubated for 2 h. The precipitate was dissolved in DMSO (0.1 mL per well). The optical density in the wells was measured on a Thermo Multiskan FC plate photometer (Thermo Fisher Scientific, USA) at a wavelength of 540 nm. Based on the obtained data, CC_50_ values (the concentration of the compound, leading to a decrease in optical density by half, compared to wells without the addition of compounds) were calculated.

### 3.6. The Study of the Anti-Viral Activity

The study of the anti-viral activity was carried out by determining the reduction of the degree of cytopathic action. The experiments used the influenza A/Puerto Rico/8/34 (H1N1) virus from the collection of viral strains of Saint Petersburg Pasteur Research Institute of Epidemiology and Microbiology. The studied compounds, in the range of concentrations, were applied to the cells in the wells of the plate and incubated for 1 h. Then, the cells were infected with the virus at a dose of 0.01 TCID_50_ per cell. The cells were incubated for 72 h, after which cell survival was analyzed using the methyltetrazolium assay, as described above. Based on the obtained data, a 50% inhibitory concentration (IC_50_) was calculated for each compound as the concentration that reduced the degree of viral destruction of cells by 50%, and the selectivity index (SI) as the ratio of CC_50_ to IC_50_. Compounds with an SI of 10 or higher were considered active.

## 4. Conclusions

As a result of the present work, new pathways for the synthesis of (Z)-7-benzylidene-[1,2,4]triazolo[3,4-*b*][1,3,4]thiadiazines **6**, **8** and 8-phenyl-[1,2,4]triazolo[3,4-*b*][1,3,4]thiadiazepines **7**, **10** have been developed based on the interaction of 3-phenyl-2-propynal **3** and 3-substituted 4-amino-4*H*-1,2,4-triazole-5-thiols **4**. It was shown that azomethines **1** could be obtained using acid catalysis, and their cyclization led to the formation of a mixture of isomeric compounds **6** and **7**. At the same time, cyclization of amines **2** led to the formation of only thiadiazines **8** in high yields. Thiadiazines **6** were obtained by dehydrogenation of the C-N bond of the compounds **8** using manganese dioxide. Thiadiazepines **7** were obtained by introducing tert-butylamine into the reaction between 3-phenyl-2-propynal **3** and 3-substituted 4-amino-4*H*-1,2,4-triazole-5-thiols **4**, which made it possible to reduce the reaction time and decrease the reaction temperature, compared to the method described in the literature [35].

Among the 20 compounds studied, 10 (50%) demonstrated low cytotoxicity and high activity against influenza virus A/Puerto Rico/8/34 (H1N1) with selectivity index (SI) of 10 and higher, and one of them possessed SI of >300. The results suggested that this group of compounds could be considered prospective for further focus and development of effective antivirals against influenza virus.

Docking results and values showed that the preferred interactant for our ligands was M2 channel. All ligands showed better GlideScore values in the M2 channel active cavity. Predicted affinity to neuraminidase for all ligands was significantly lower than control compound (oseltamivir).

Protein–ligand interaction modeling showed that the aliphatic moiety of ligands could negatively regulate target activity levels via changes in lipophilic interaction balance between core and sidechains. This can be explained by strain energy value.

## Figures and Tables

**Table 1:** Crystal data and structure refinement for **6a**, **7c**, **8a**, **8c** and **10a**.

Parameter	6a	7c	8a	8c	10a
Crystal system	orthorhombic	monoclinic	monoclinic	triclinic	monoclinic
Space group	P2_1_2_1_2_1_	P2_1_/c	P2_1_/c	P-1	P2_1_/c
a/Å	3.84480(10)	22.4518(5)	22.7514(9)	9.6530(5)	9.0504(3)
b/Å	6.6325(2)	3.92160(10)	7.8068(3)	9.7098(5)	9.9977(3)
c/Å	39.1172(15)	13.8956(3)	12.3438(5)	13.7763(6)	12.1907(4)
α/°	90	90	90	93.858(4)	90
β/°	90	94.485(2)	105.173(4)	100.186(4)	111.125(4)
γ/°	90	90	90	91.383(4)	90
Volume/Å^3^	997.51(6)	1219.72(5)	2116.03(15)	1267.15(11)	1028.92(6)
Z	4	4	8	4	4
ρ_calc_g/cm^3^	1.520	1.396	1.446	1.354	1.487
μ/mm^−1^	2.669	2.243	0.281	0.243	2.588
F(000)	472.0	536.0	960.0	544.0	480.0
Crystal size/mm^3^	0.1 × 0.05 × 0.03	0.1 × 0.05 × 0.03	0.30 × 0.22 × 0.12	0.32 × 0.20 × 0.14	0.15 × 0.09 × 0.06
Radiation	Cu Kα (λ = 1.54184 Å)	Cu Kα (λ = 1.54184 Å)	MoKα (λ = 0.71073 Å)	MoKα (λ = 0.71073 Å)	Cu Kα (λ = 1.54184 Å)
2Θ range for data collection/°	4.518 to 139.91	3.948 to 138.074	5.538 to 54.998	5.666 to 54.998	10.478 to 152.372
Index ranges	−4 ≤ h ≤ 3, −7 ≤ k ≤ 8, −43 ≤ l ≤ 47	−27 ≤ h ≤ 24, −4 ≤ k ≤ 4, −16 ≤ l ≤ 15	−29 ≤ h ≤ 24, −10 ≤ k ≤ 10, −11 ≤ l ≤ 16	−12 ≤ h ≤ 12, −12 ≤ k ≤ 10, −17 ≤ l ≤ 17	−11 ≤ h ≤ 10, −12 ≤ k ≤ 12, −15 ≤ l ≤ 15
Reflections collected	4912	9507	9608	10799	8969
Independent reflections	1892 [R_int_ = 0.0506, R_sigma_ = 0.0467]	2276 [R_int_ = 0.0334, R_sigma_ = 0.0288]	4823 [R_int_ = 0.0283, R_sigma_ = 0.0468]	5805 [R_int_ = 0.0268, R_sigma_ = 0.0463]	2128 [R_int_ = 0.0367, R_sigma_ = 0.0270]
Data/restraints/ parameters	1892/126/145	2276/194/209	4823/0/297	5805/0/335	2128/0/149
Goodness-of-fit on F^2^	1.112	1.081	1.177	1.028	1.061
Final R indexes [I ≥ 2σ (I)]	R_1_ = 0.0552, wR_2_ = 0.1376	R_1_ = 0.0415, wR_2_ = 0.1043	R_1_ = 0.0710, wR_2_ = 0.1707	R_1_ = 0.0390, wR_2_ = 0.0862	R_1_ = 0.0378, wR_2_ = 0.0928
Final R indexes [all data]	R_1_ = 0.0571, wR_2_ = 0.1384	R_1_ = 0.0443, wR_2_ = 0.1061	R_1_ = 0.0809, wR_2_ = 0.1772	R_1_ = 0.0514, wR_2_ = 0.0943	R_1_ = 0.0414, wR_2_ = 0.0946
Largest diff. peak/hole/e Å^−3^	0.64/−0.50	0.43/−0.48	1.62/−0.43	0.36/−0.29	0.39/−0.34
Flack parameter	0.00(3)	-	-	-	-
CCDC	2212189	2212192	1813554	1813556	2212194

**Table 2:** Cytotoxic and anti-viral properties of synthesized compounds **1a–c**, **2a–d**, **6a–d**, **7a–d**, **8a–d**, **10a–d** according to in vitro tests against influenza virus A/Puerto Rico/8/34 in MDCK cells.

Compound	^a^ CC_50_, µM	^b^ IC_50_, µM	^c^ SI
**1a**	134 ± 11	16.2 ± 2.1	8
**1b**	125.5 ± 11.3	24.8 ± 2.9	5
**1c**	119.4 ± 9.6	11.3 ± 2.2	11
**2a**	>1300	4.3 ± 1.0	>300
**2b**	>1200	106.4 ± 12.5	>12
**2c**	487.3 ± 22.5	37.2 ± 4.1	13
**2d**	138 ± 12.0	100.9 ± 12.4	1
**6a**	578.2 ± 32.3	34.2 ± 4.2	17
**6b**	433.3 ± 37.6	99.9 ± 11.8	4
**6c**	979 ± 55.1	117.8 ± 12.7	8
**6d**	>1100	33.6 ± 4.4	>33
**7a**	42.9 ± 3.2	>13	<3
**7b**	NT	NT	NT
**7c**	6.1 ± 0.3	>3	<2
**7d**	NT	NT	NT
**8a**	>1300	39.5 ± 5.1	>33
**8b**	>1200	>1200	1
**8c**	>1100	50.3 ± 6.2	>23
**8d**	>1100	58 ± 6.6	>19
**10a**	>1300	703 ± 82.9	>2
**10b**	>1100	41.4 ± 5.1	27
**10c**	971.7 ± 66.4	>300	<3
**10d**	NT	NT	NT
**Rimantadine**	312.3 ± 22.8	64.1 ± 7.2	5
**Oseltamivir** **carboxylate**	>100	0.17 ± 0.02	>588

^a^ CC_50_, cytotoxic concentration; the concentration resulting in 50% death of cells; ^b^ IC_50_, effective concentration; the concentration resulting in 50% inhibition of virus replication; ^c^ SI, selectivity index; ratio CC_50_/IC_50_; NT—not tested.

**Table 3:** Glide score values and MM-GBSA free energy components calculated for best-fitting binding poses of observed ligands in comparison with IC_50_ values.

Compound	Glidescore, (kcal/mol)		MMGBSA ΔG, (kcal/mol)		IC_50_
	Neuraminidase	M2	M2—Lipo	M2—Strain Energy	
**1a**	−4.39	−6.42	−12.06	0.85	16.2 ± 2.1
**1b**	−3.72	−6.66	−11.49	2.57	24.8 ± 2.9
**1c**	−4.07	−6.96	−14.21	1.11	11.3 ± 2.2
**2a**	−4.18	−6.12	−16.42	0.68	4.3 ± 1.0
**2b**	−4.57	−6.38	−13.41	1.56	106.4 ± 12.5
**2c**	−4.41	−6.50	−15.80	0.45	37.2 ± 4.1
**2d**	−3.48	−5.96	−14.62	1.82	100.9 ± 12.4
**6a**	−4.52	−6.09	−12.25	0.41	34.2 ± 4.2
**6b**	−4.28	−6.15	−10.91	0.58	99.9 ± 11.8
**6c**	−4.19	−6.15	−11.56	1.37	117.8 ± 12.7
**6d**	−3.85	−6.00	−12.20	0.70	33.6 ± 4.4
**7a**	−4.15	−6.48	−10.22	1.27	>13
**7c**	−3.75	−6.30	−13.35	0.50	>3
**8a**	−4.53	−6.70	−13.75	0.38	39.5 ± 5.1
**8b**	−4.50	−6.19	−11.62	0.34	>1200
**8c**	−4.42	−6.43	−11.99	0.42	50.3 ± 6.2
**8d**	−4.20	−6.38	−12.70	0.61	58 ± 6.6
**10a**	−4.78	−6.54	−11.51	0.51	703 ± 82.9
**10b**	−3.41	−6.47	−14.77	0.75	41.4 ± 5.1
**10c**	−3.40	−6.37	−13.02	1.70	>300
**Rimantadine**	–	−6.60	−13.25	0.78	64.1 ± 7.2
**Oseltamivir**	−5.50	–	–	–	0.17 ± 0.02

## Data Availability

Not applicable.

## Supplementary Materials

The following supporting information can be downloaded at: https://www.mdpi.com/article/10.3390/molecules27227940/s1, Spectral data of the new compounds **1a–c**, **2a–d**, **6a–d**, **7a–d**, **8a–d**, **10a–d**.

## Sample Availability

Samples of the compounds **1a–c, 2a–d, 6a–d, 7a–d, 8a–d, 10a–d** are available from the authors.
