# Supplementary material for: Novel [1,2,4]triazolo[3,4-b][1,3,4]thiadiazine and [1,2,4]triazolo[3,4-b][1,3,4]thiadiazepine Derivatives: Synthesis, Anti-Viral In Vitro Study and Target Validation Activity
Source: Molecules. 2022 Nov 16;27(22):7940. doi: 10.3390/molecules27227940 (PMC9694146; doi:10.3390/molecules27227940)
Supplement: Supplementary file 1 [file molecules-27-07940-s001.zip › molecules-2023710-supplementary.pdf]

S1–S23. ^{1}H NMR spectra of the new compounds **1a–c**, **2a–d**, **6a–d**, **7a–d**, **8a–d**, **10a–d**	**pp 3–25**
S24–S46. ^{13}C NMR spectra of the new compounds **1a–c**, **2a–d**, **6a–d**, **7a–d**, **8a–d**, **10a–d**	**pp 26–48**
S47. Ramachandran plot for the engineered model of M2 channel (strain A/Puerto Rico/8/1934 H1N1)	**p 49**

S1. ^{1}H NMR (400 MHz, DMSO-d_6) spectrum of the new compound **1a**

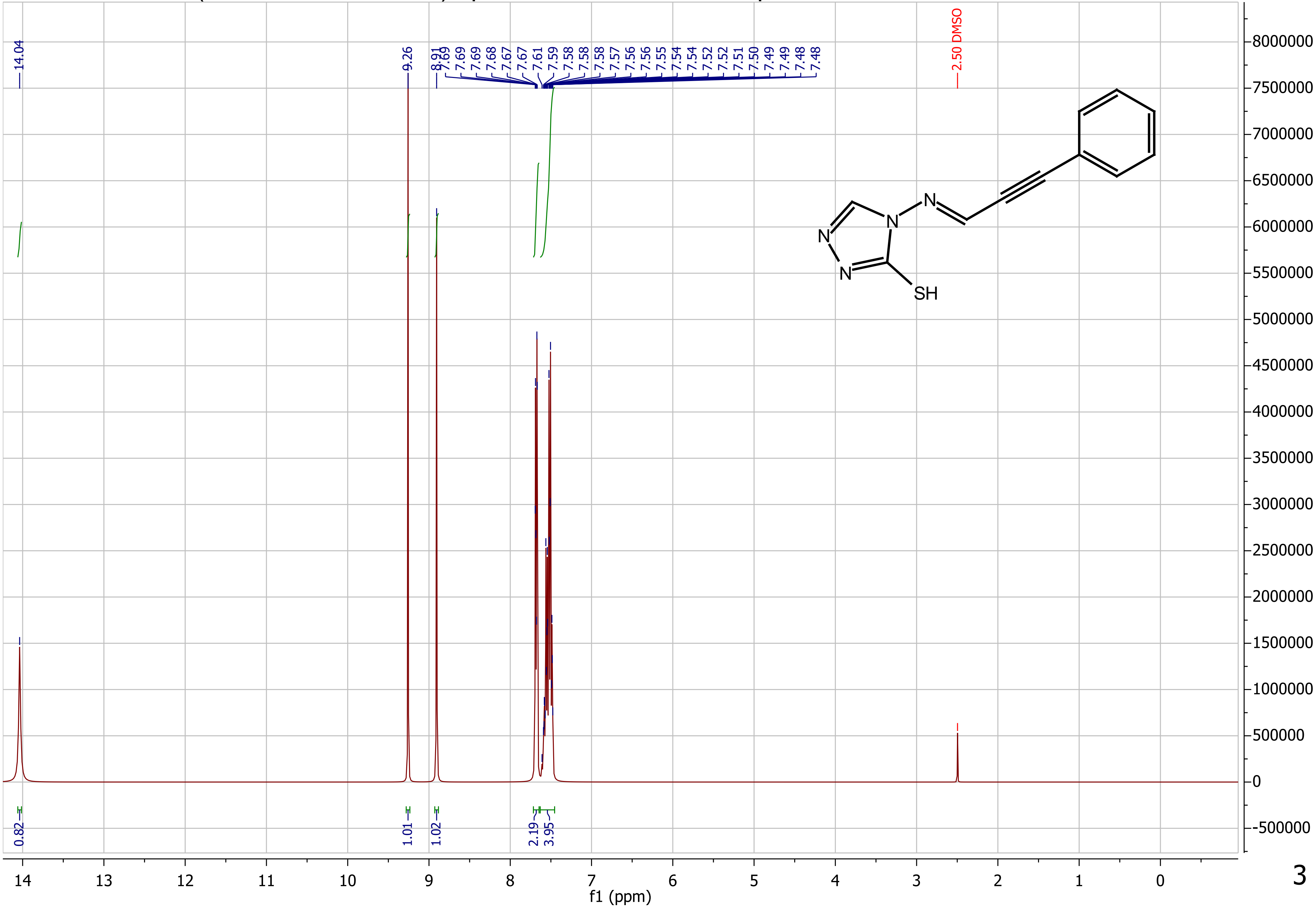

S2. ^{1}H NMR (400 MHz, DMSO-d_6) spectrum of the new compound **1b**

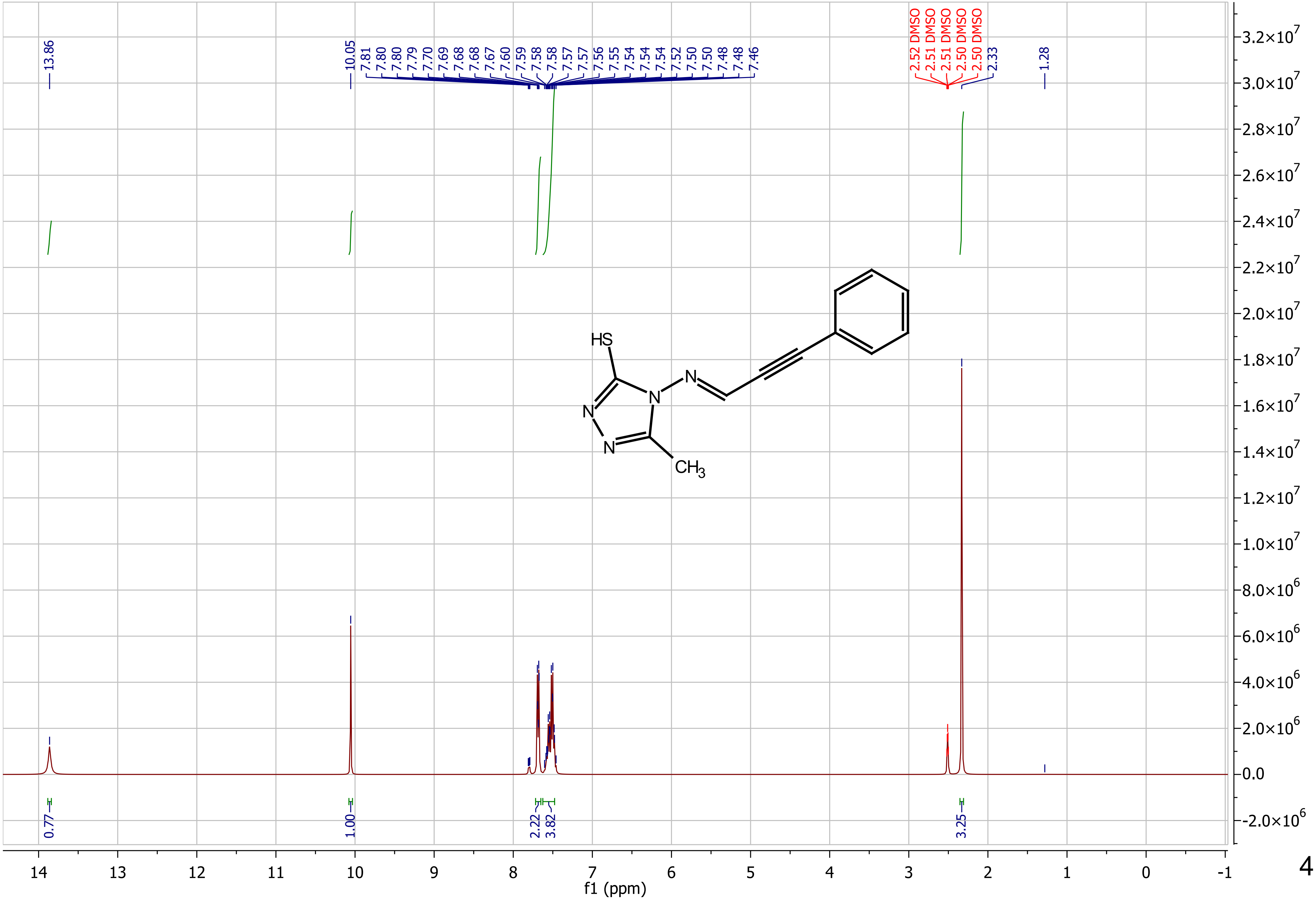

S3. ^{1}H NMR (400 MHz, DMSO-d_6) spectrum of the new compound **1c**

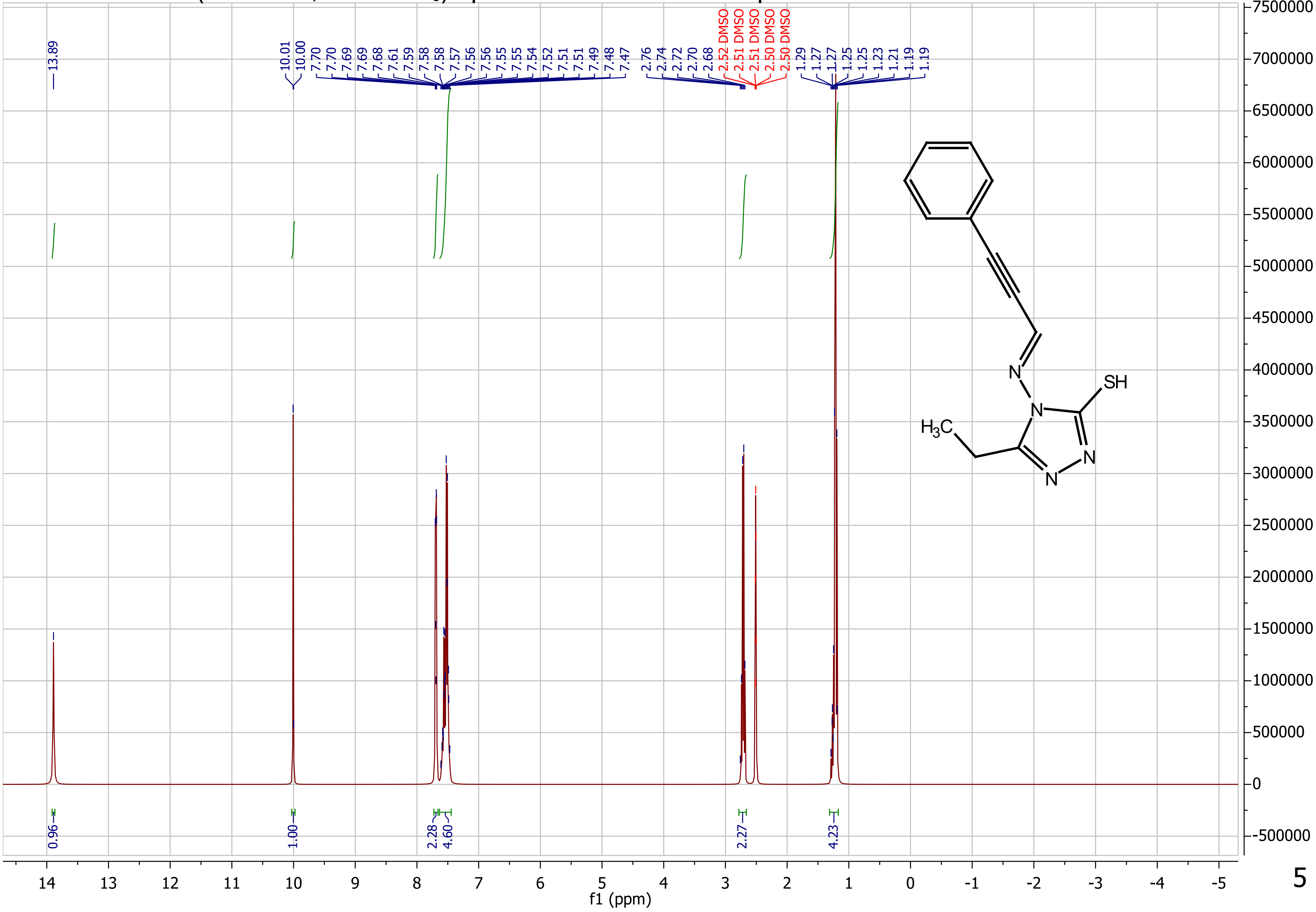

S4. ^{1}H NMR (400 MHz, DMSO-d_6) spectrum of the new compound **2a**

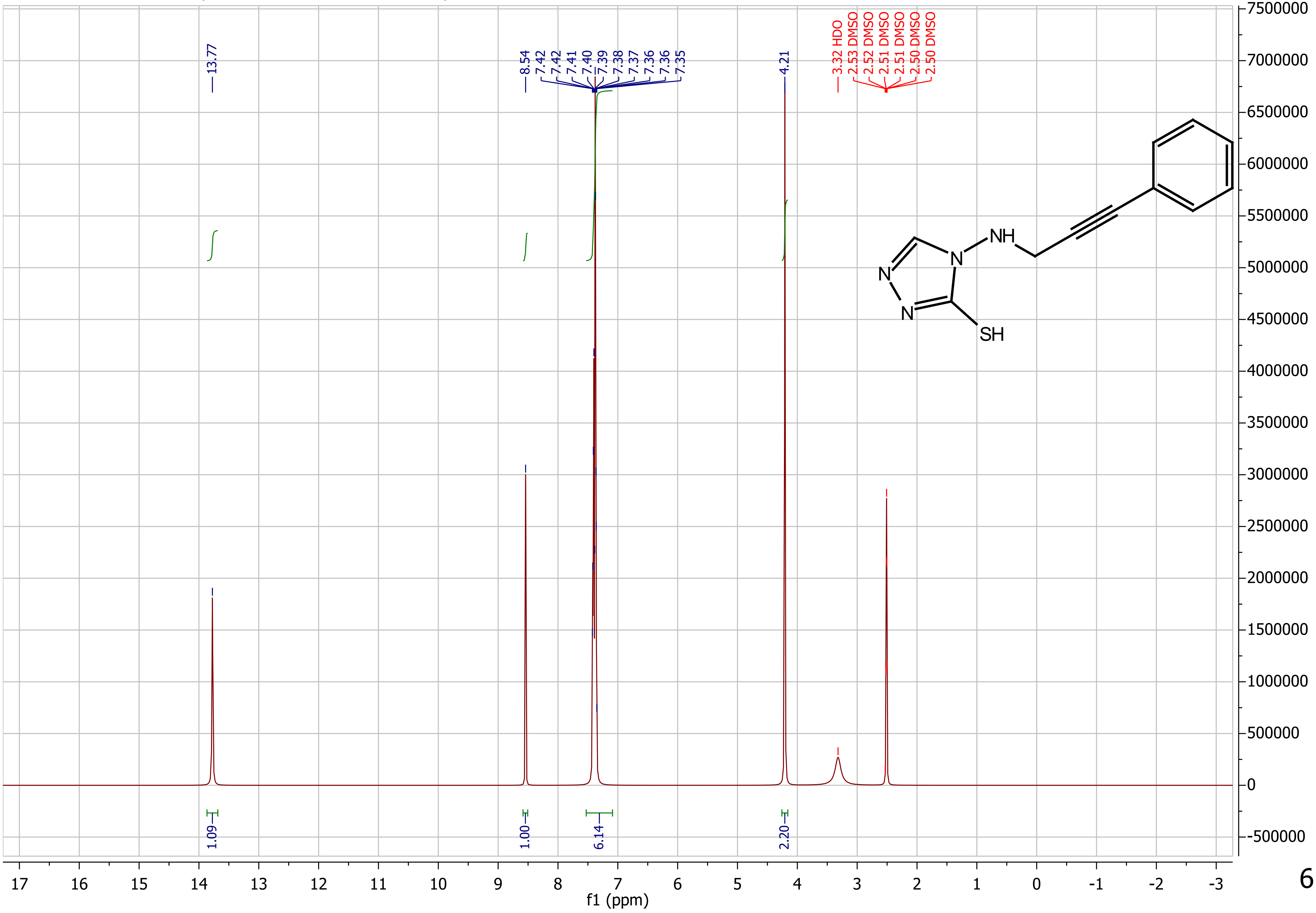

S5. ^{1}H NMR (400 MHz, DMSO-d_6) spectrum of the new compound **2b**

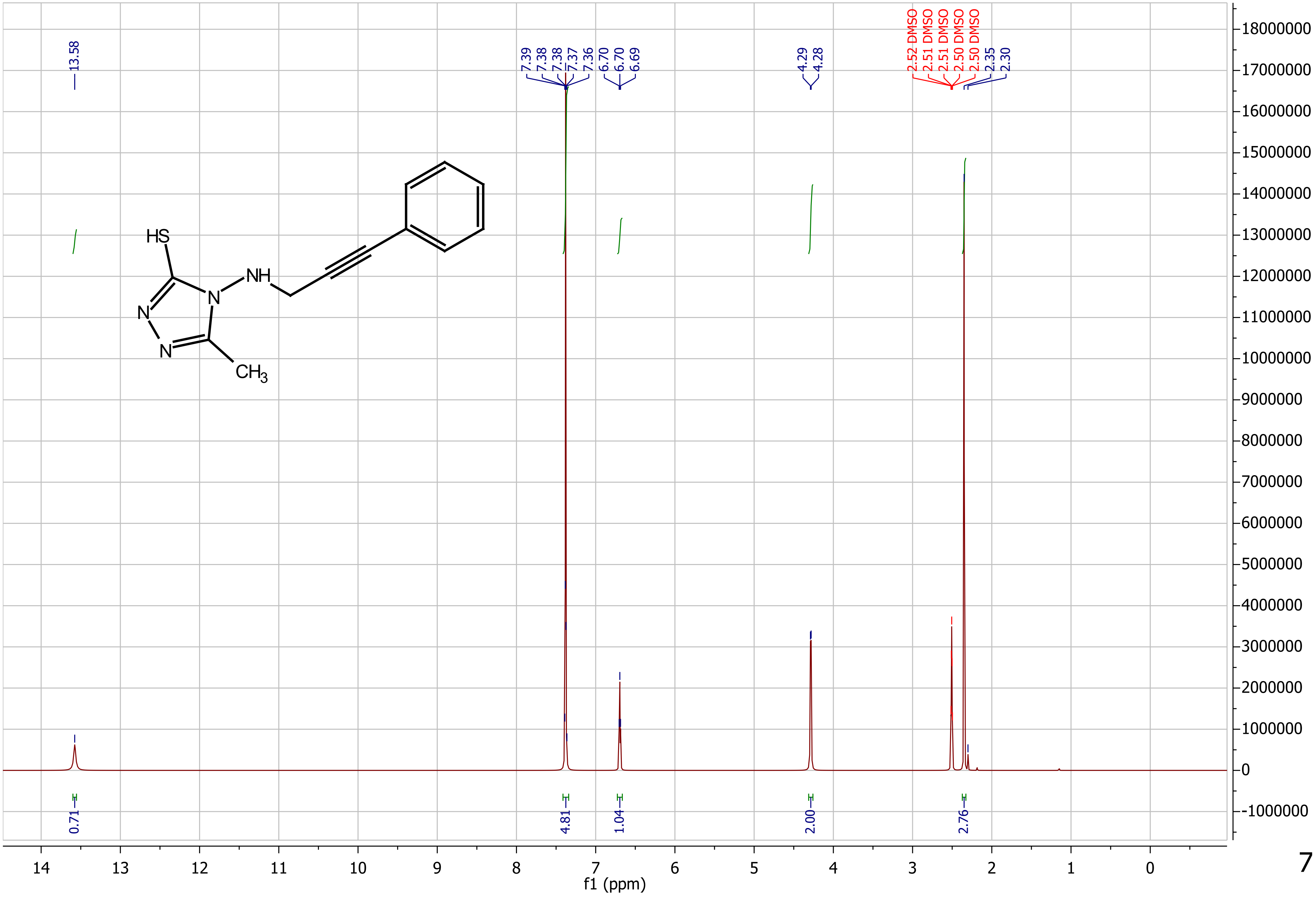

S6. ^{1}H NMR (400 MHz, DMSO-d_6) spectrum of the new compound **2c**

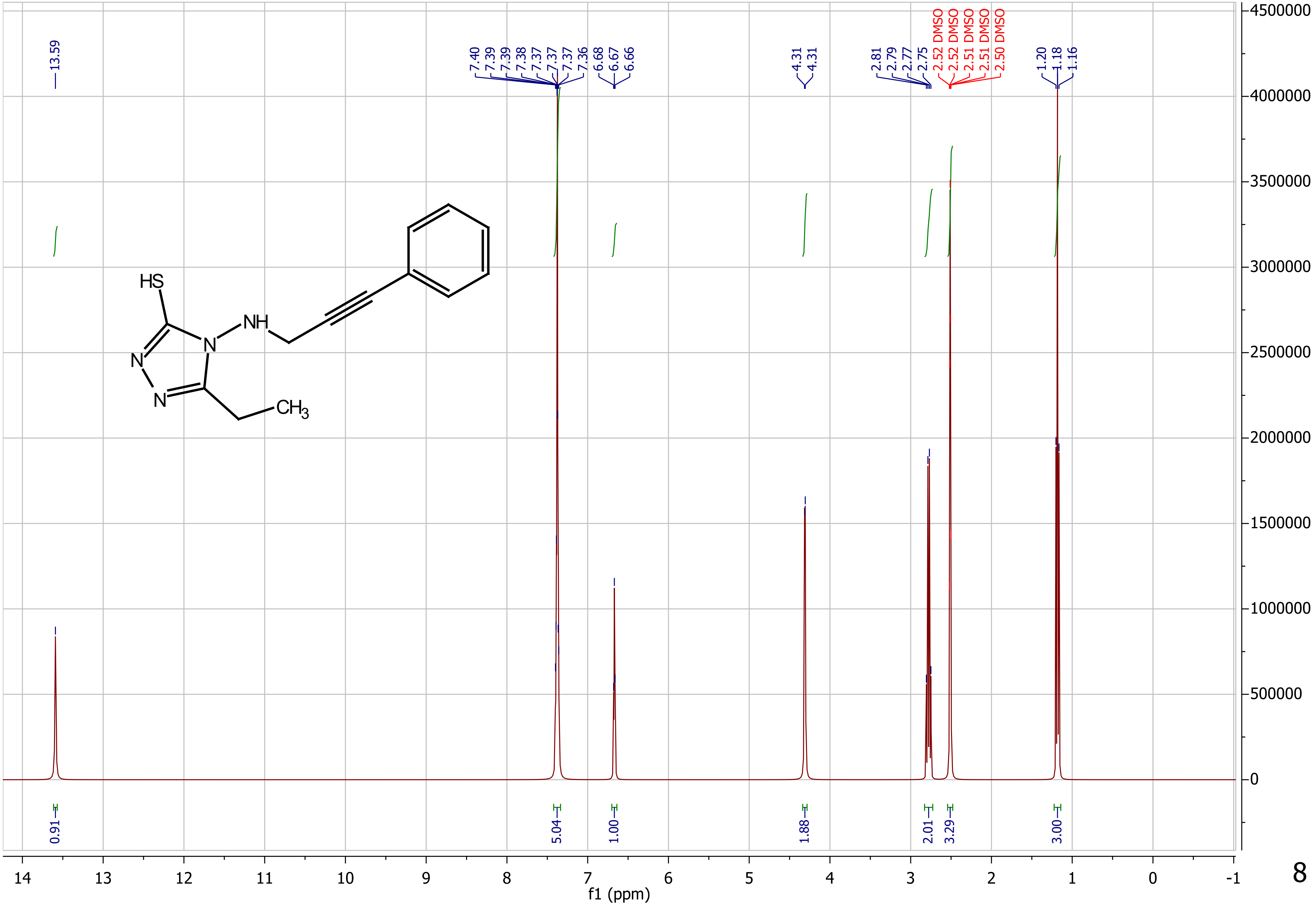

S7. ^{1}H NMR (400 MHz, DMSO-d_6) spectrum of the new compound **2d**

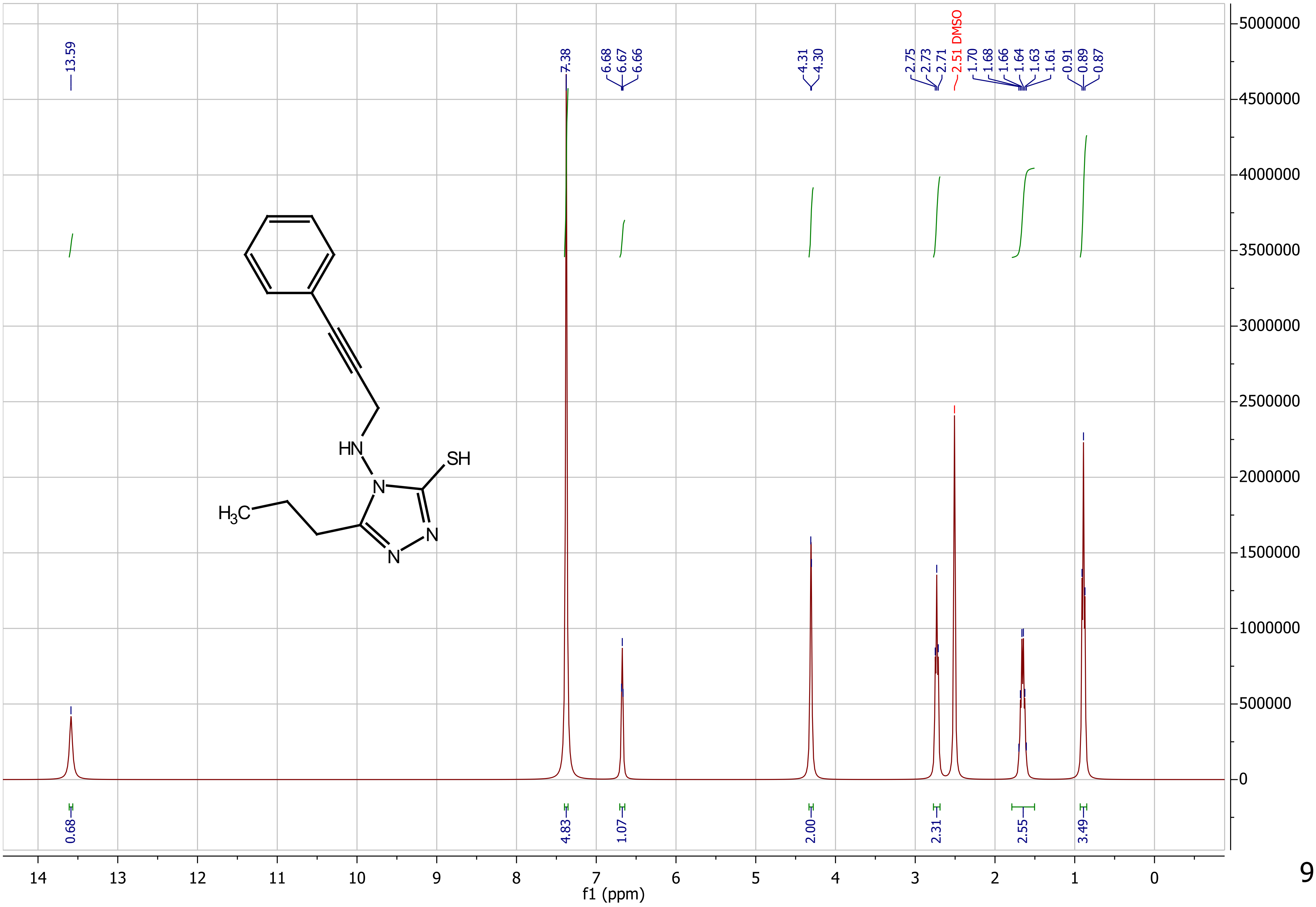

S8. ^{1}H NMR (400 MHz, DMSO-d_6) spectrum of the new compound **6a**

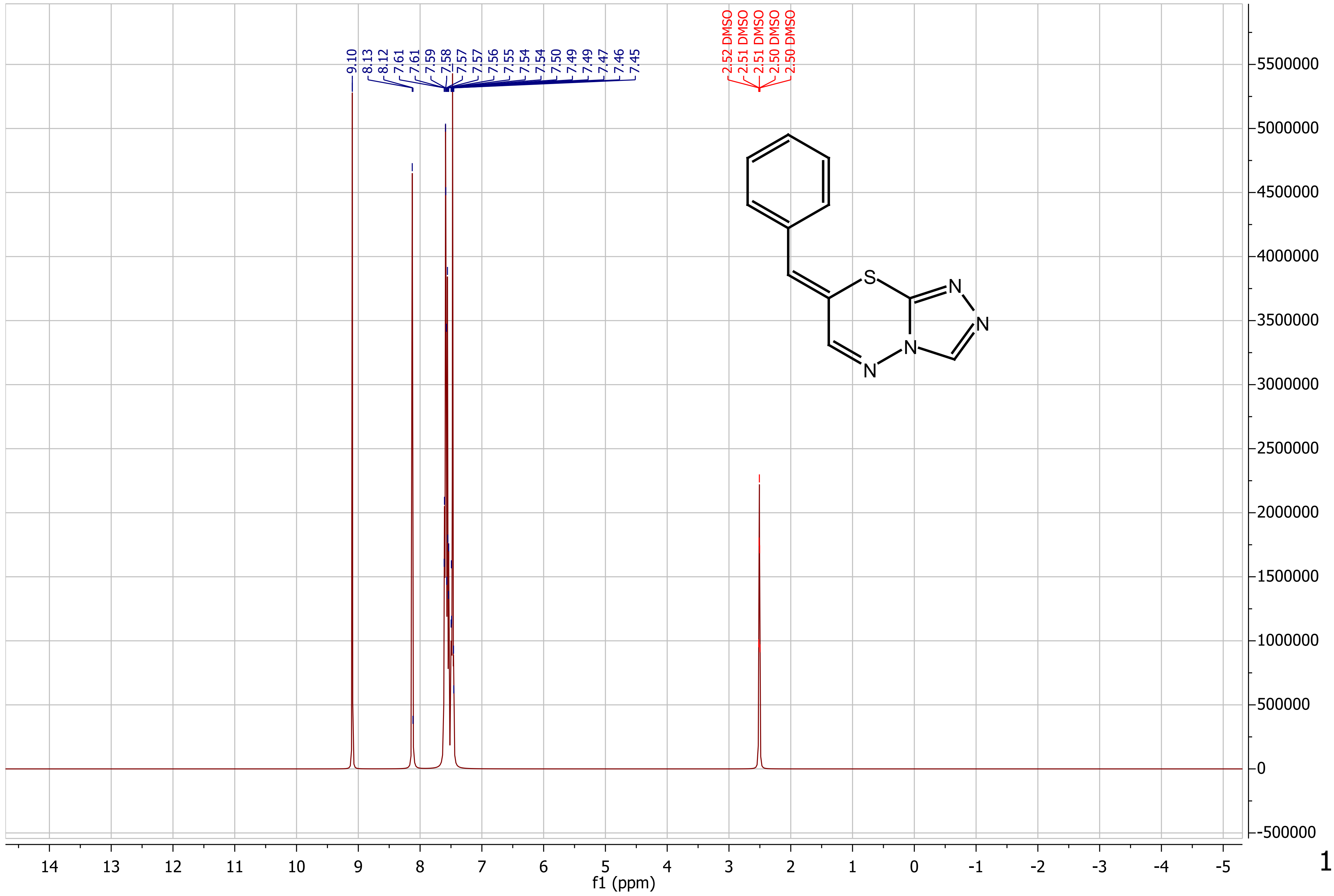

S9. ^{1}H NMR (400 MHz, DMSO-d_6) spectrum of the new compound **6b**

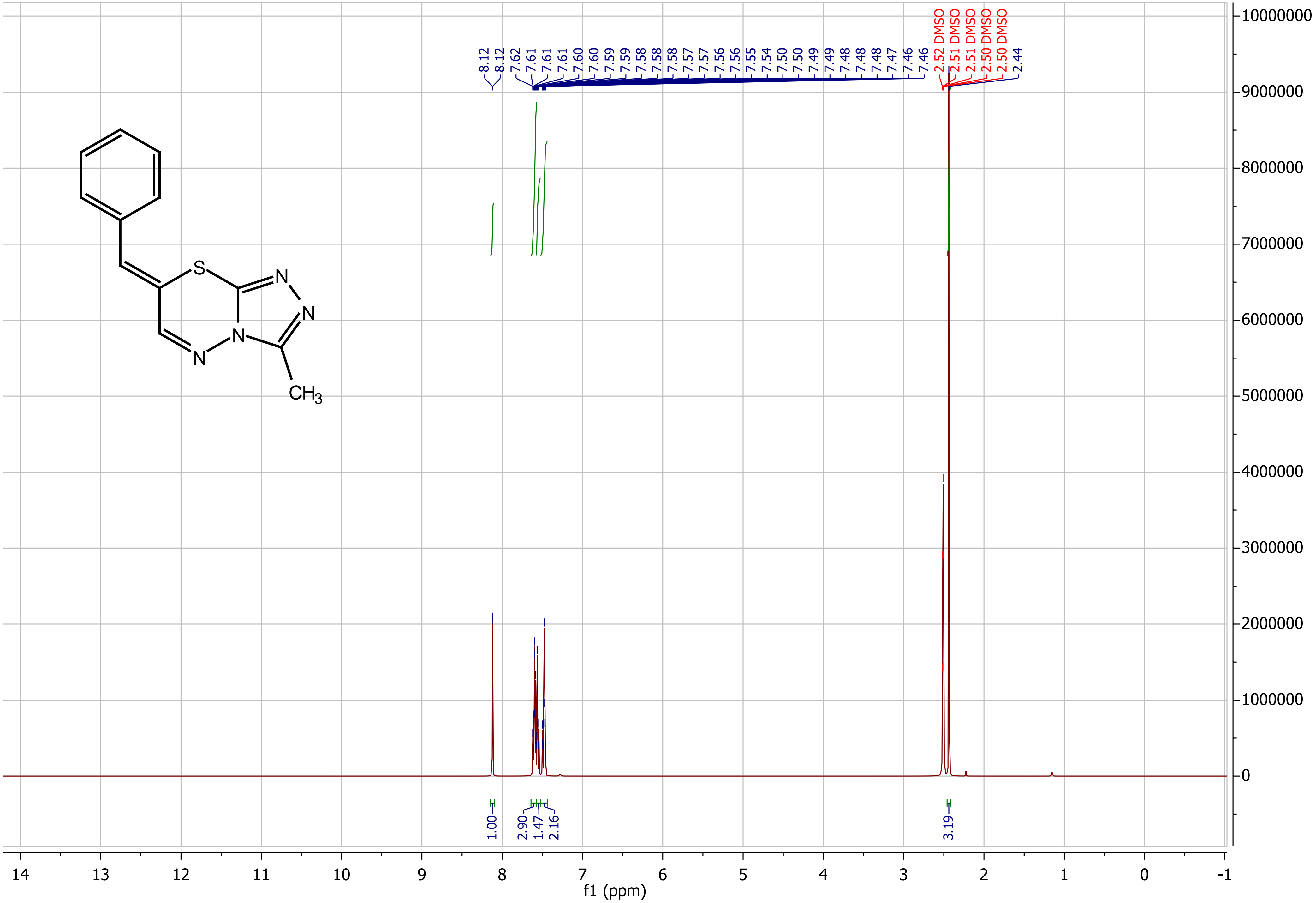

S10. ^{1}H NMR (400 MHz, DMSO-d_6) spectrum of the new compound **6c**

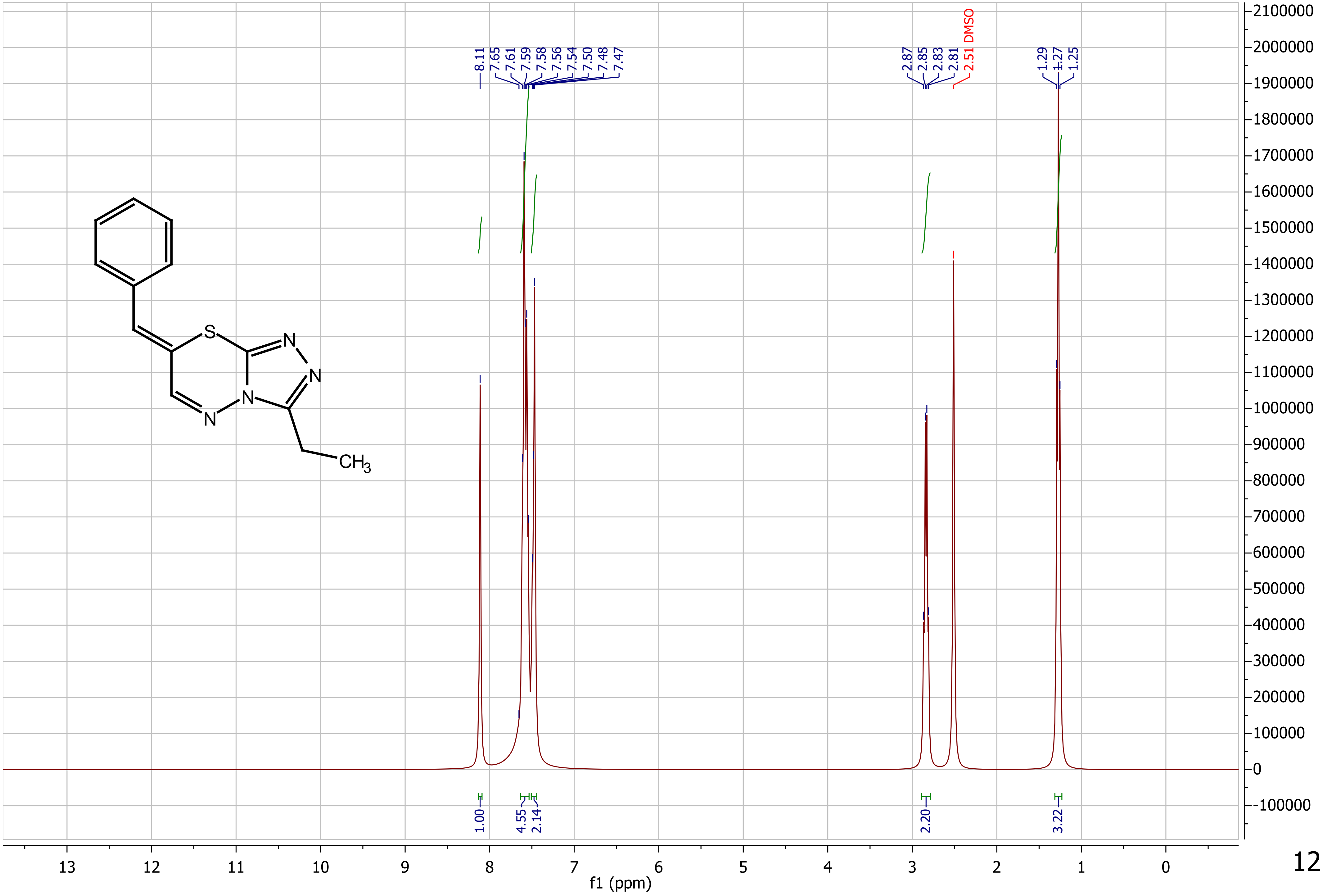

S11. ^{1}H NMR (400 MHz, DMSO-d_6) spectrum of the new compound **6d**

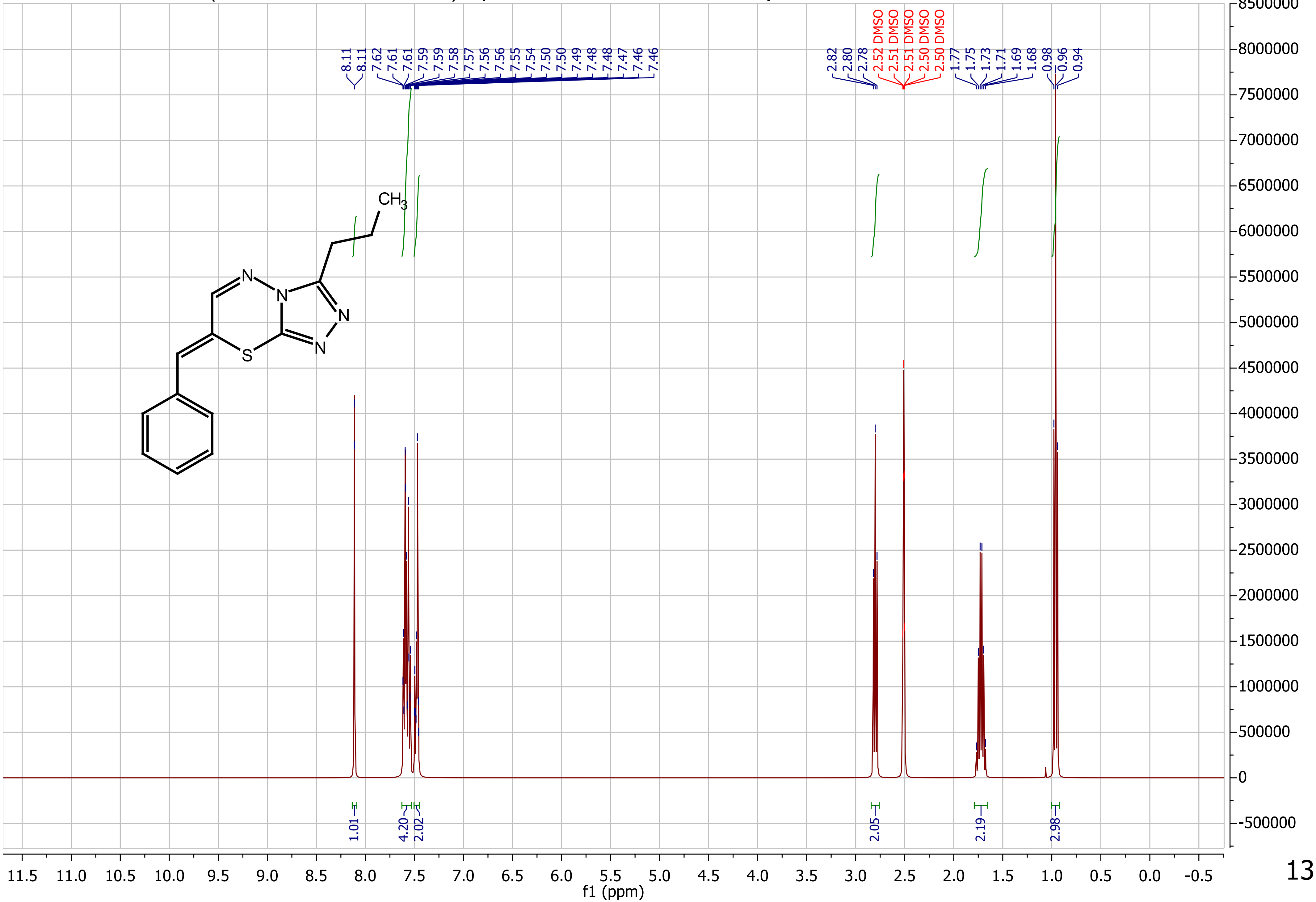

S12. ^{1}H NMR (400 MHz, DMSO-d_6) spectrum of the new compound **7a**

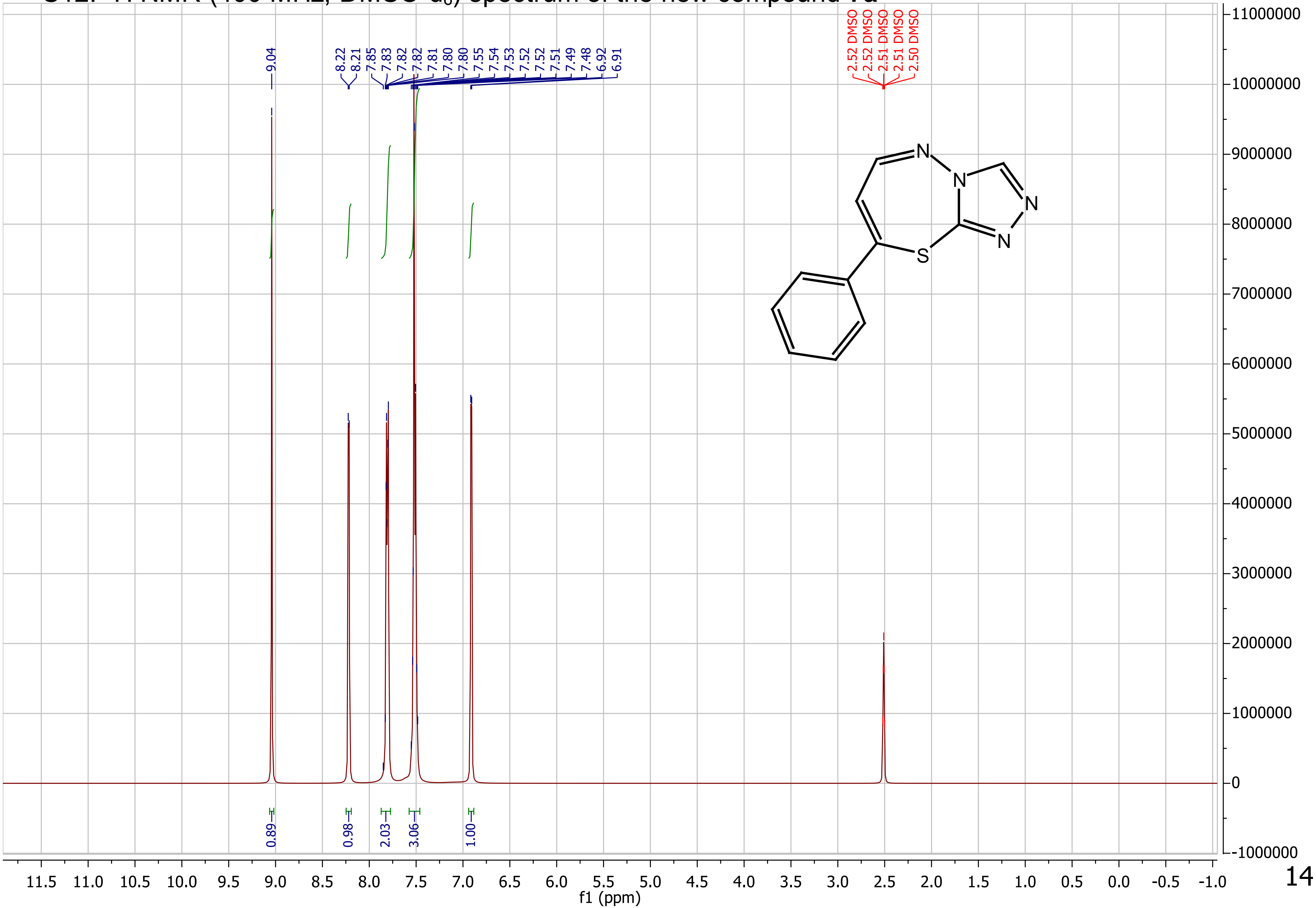

S13. ^1H NMR (400 MHz, DMSO-d_6) spectrum of the new compound **7b**

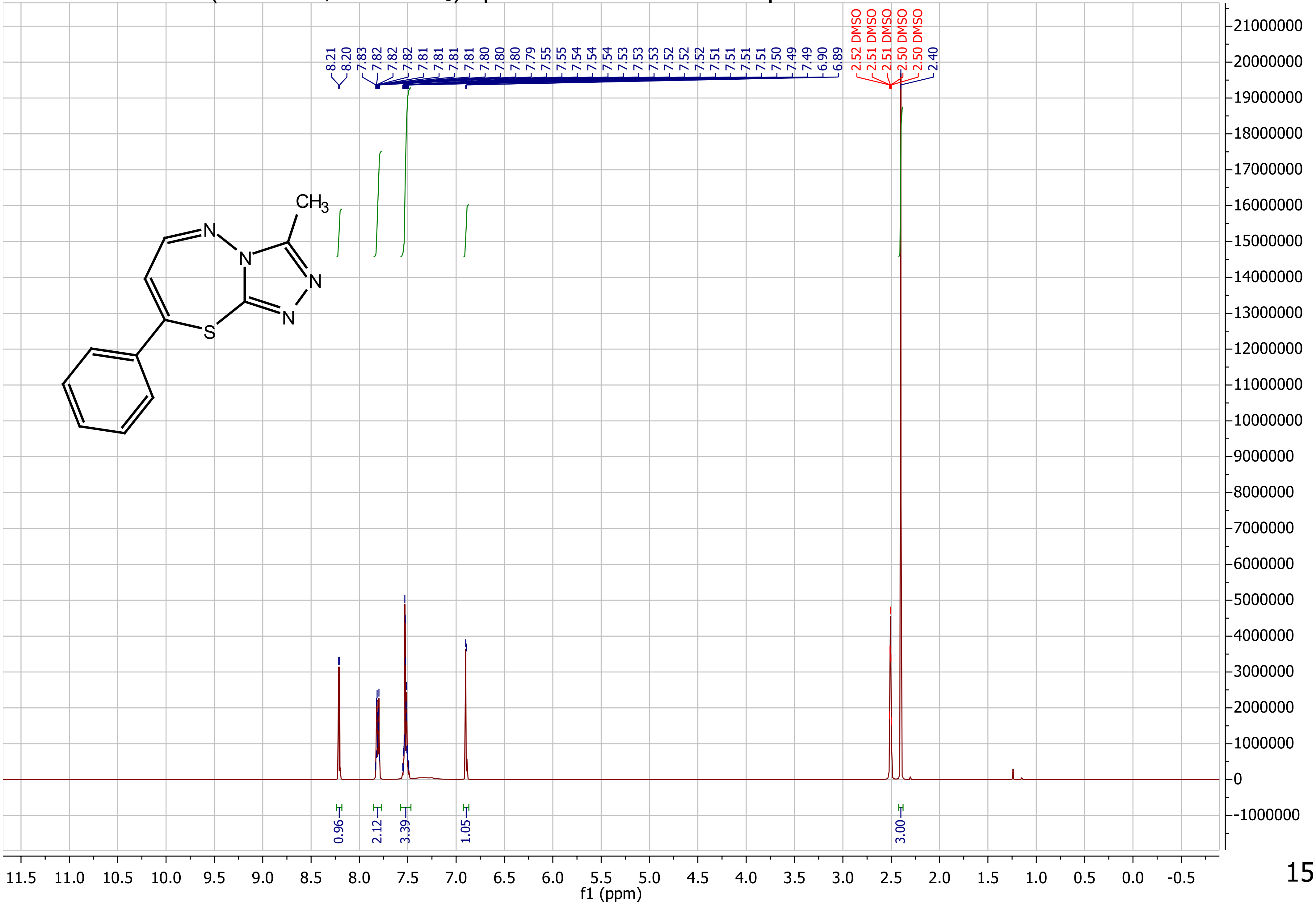

S14. ^{1}H NMR (400 MHz, DMSO-d_6) spectrum of the new compound **7c**

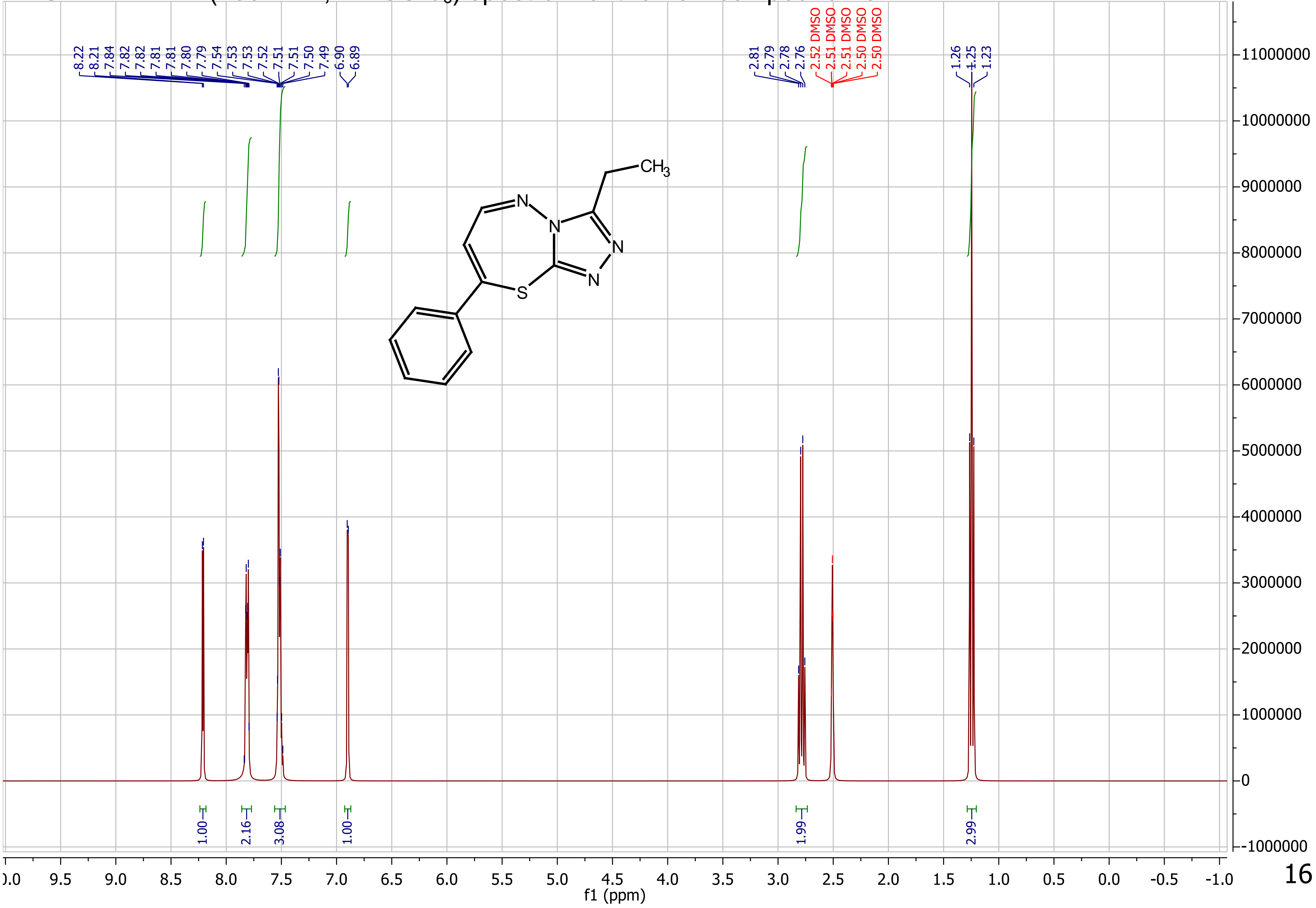

S15. ^{1}H NMR (400 MHz, DMSO-d_6) spectrum of the new compound **7d**

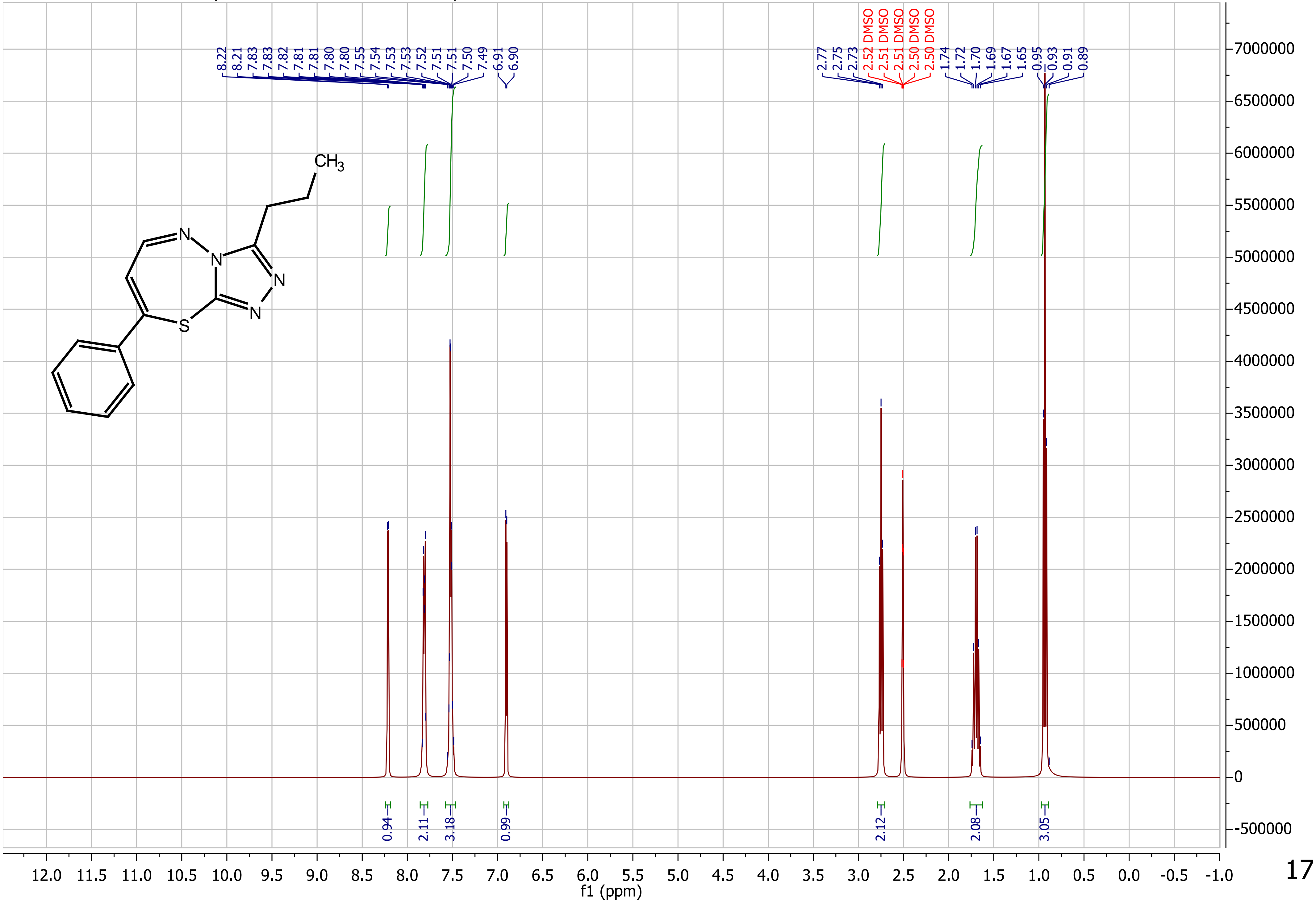

S16. ^{1}H NMR (400 MHz, DMSO-d_6) spectrum of the new compound **8a**

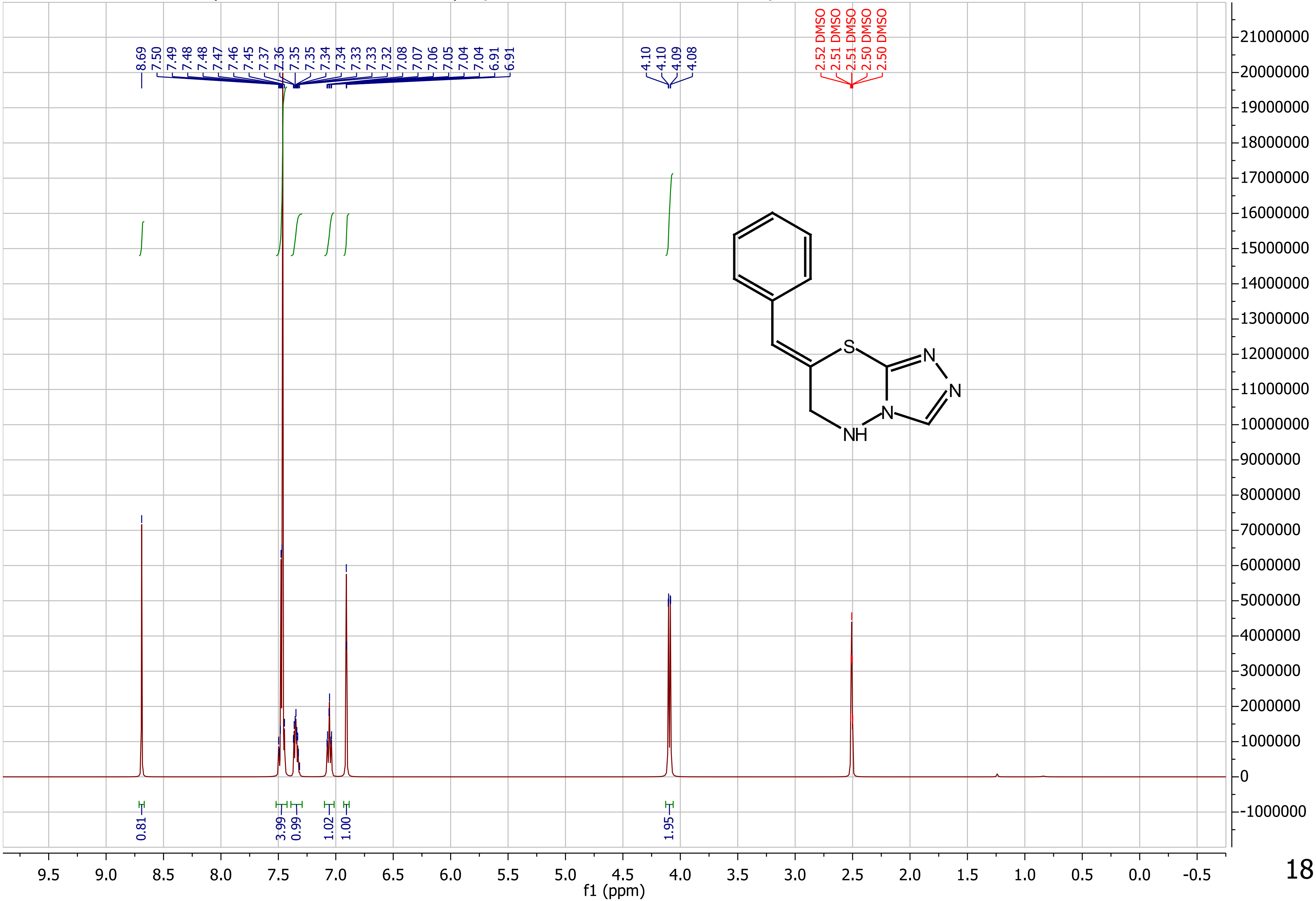

S17. ^{1}H NMR (400 MHz, DMSO-d_6) spectrum of the new compound **8b**

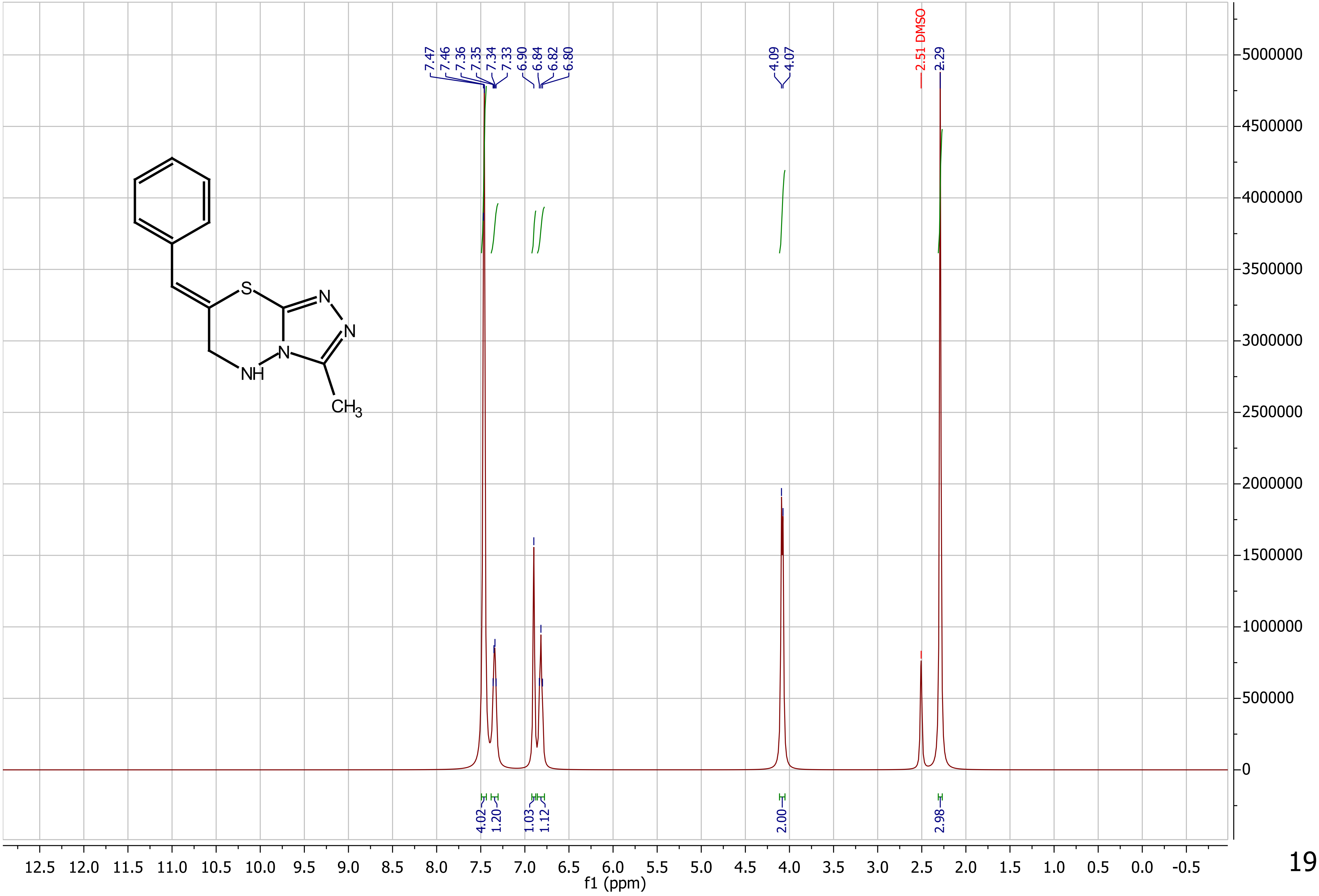

S18. ^{1}H NMR (400 MHz, DMSO-d_6) spectrum of the new compound **8c**

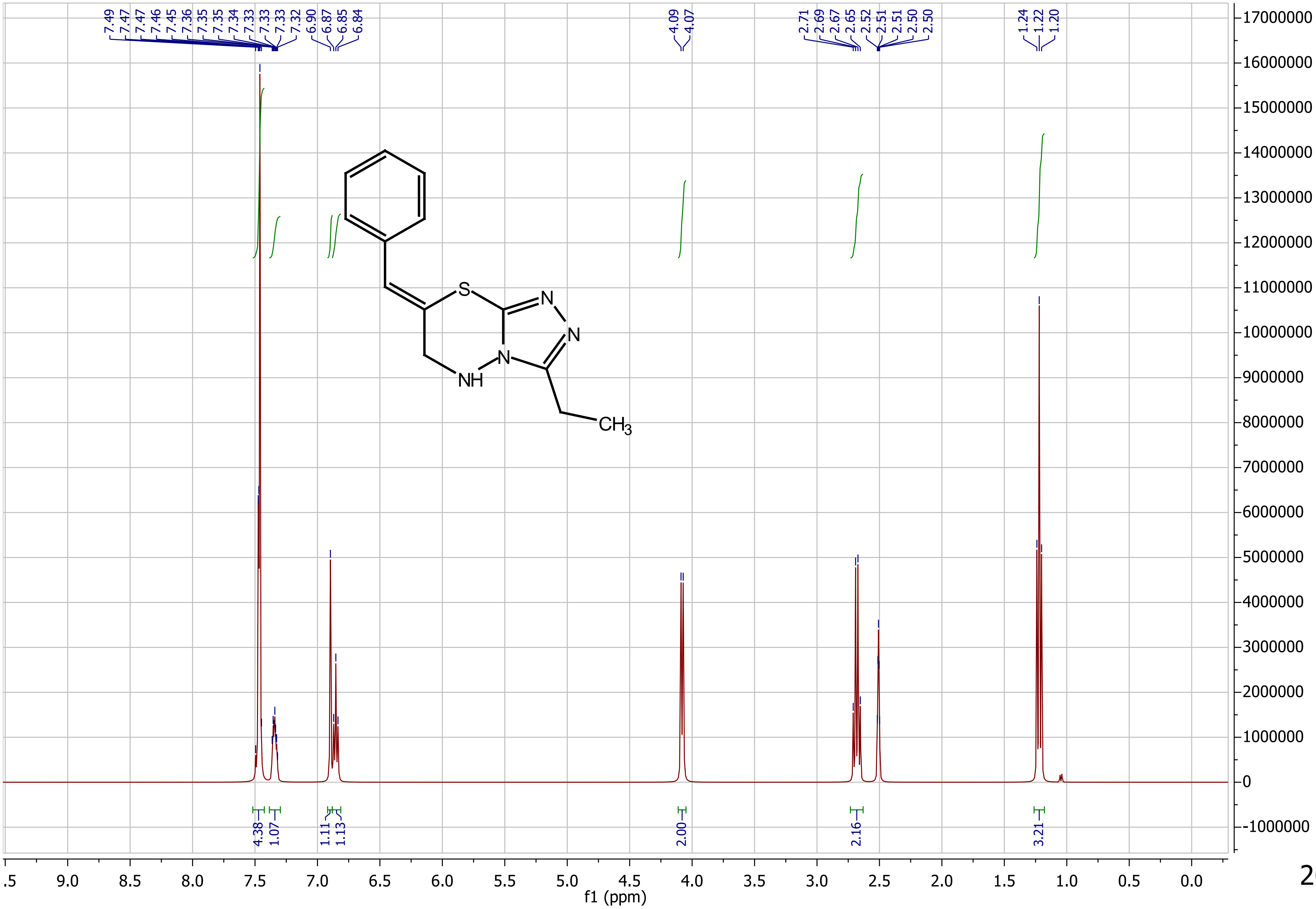

S19. ^{1}H NMR (400 MHz, DMSO-d_6) spectrum of the new compound **8d**

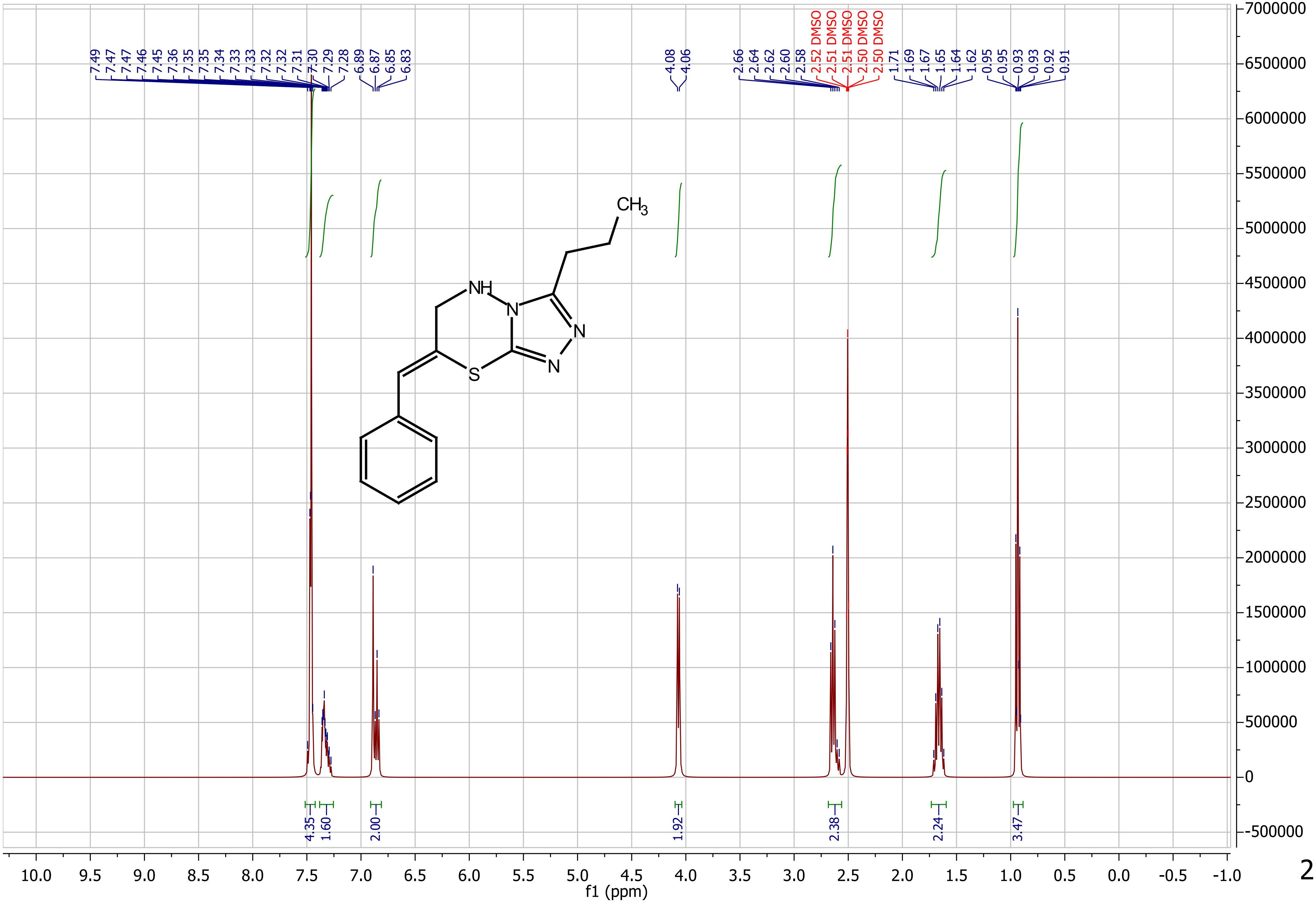

S20. ^{1}H NMR (400 MHz, DMSO-d_6) spectrum of the new compound **10a**

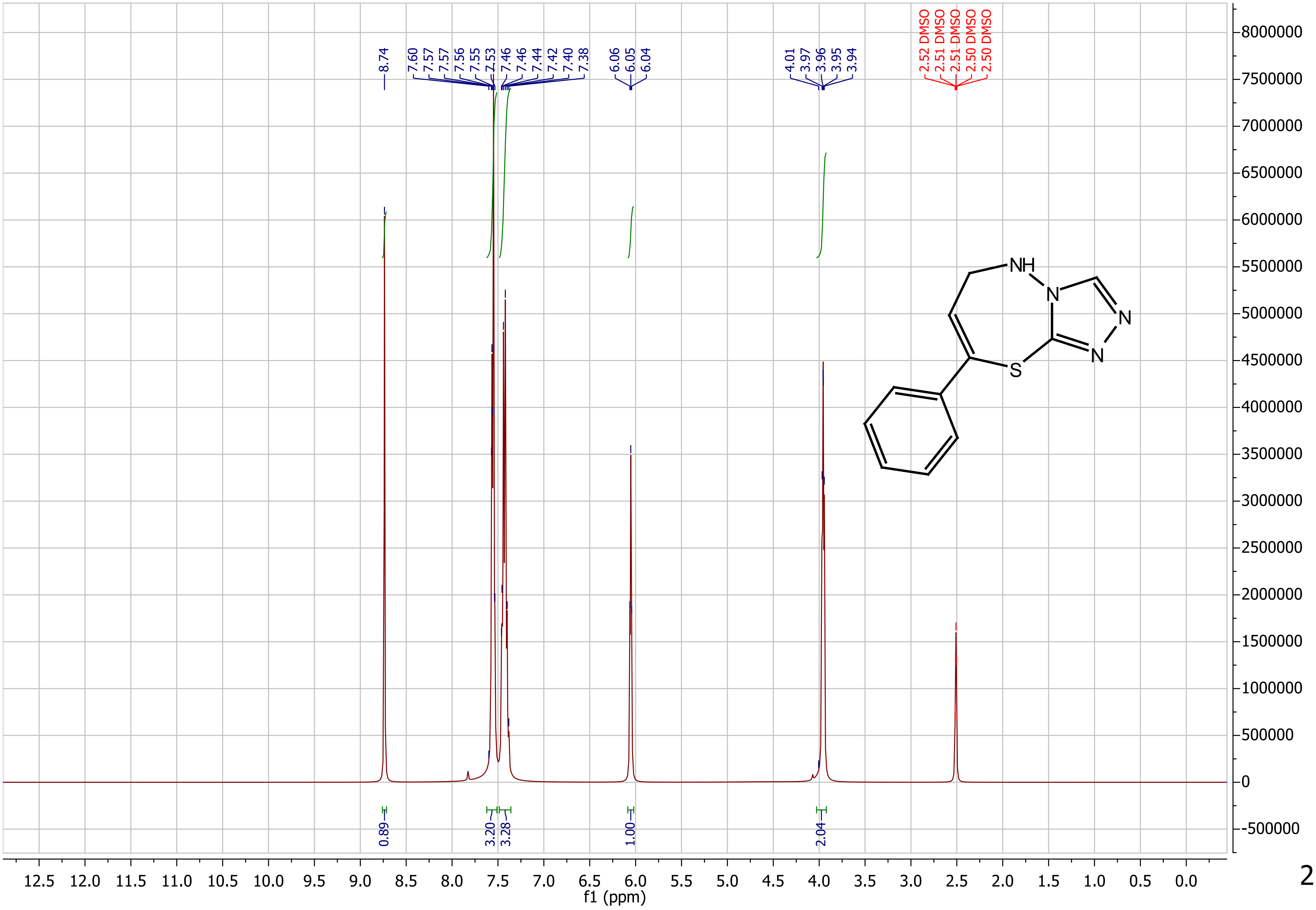

S21. ^{1}H NMR (400 MHz, DMSO-d_6) spectrum of the new compound **10b**

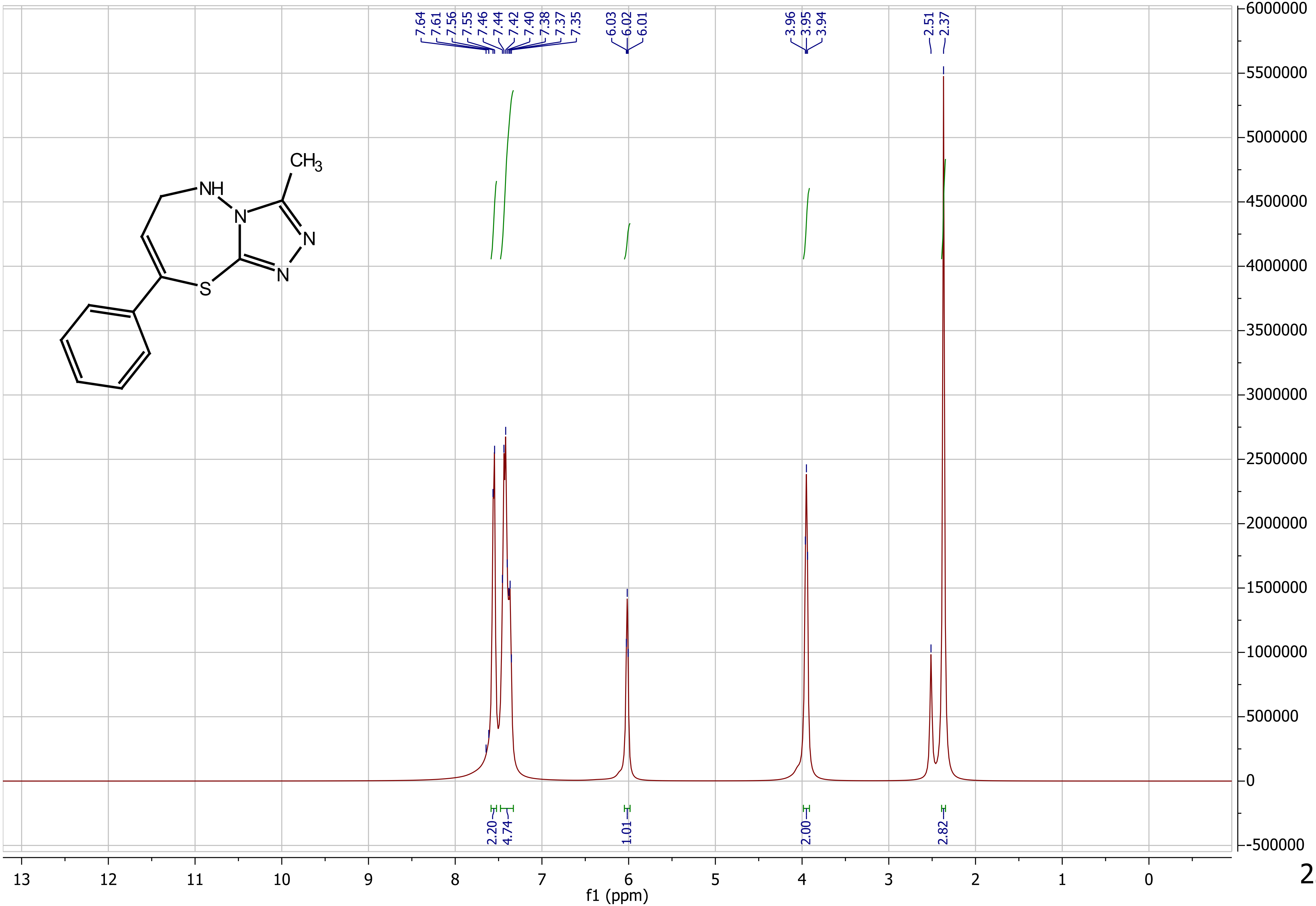

S22. ^{1}H NMR (400 MHz, DMSO-d$_6$) spectrum of the new compound **10c**

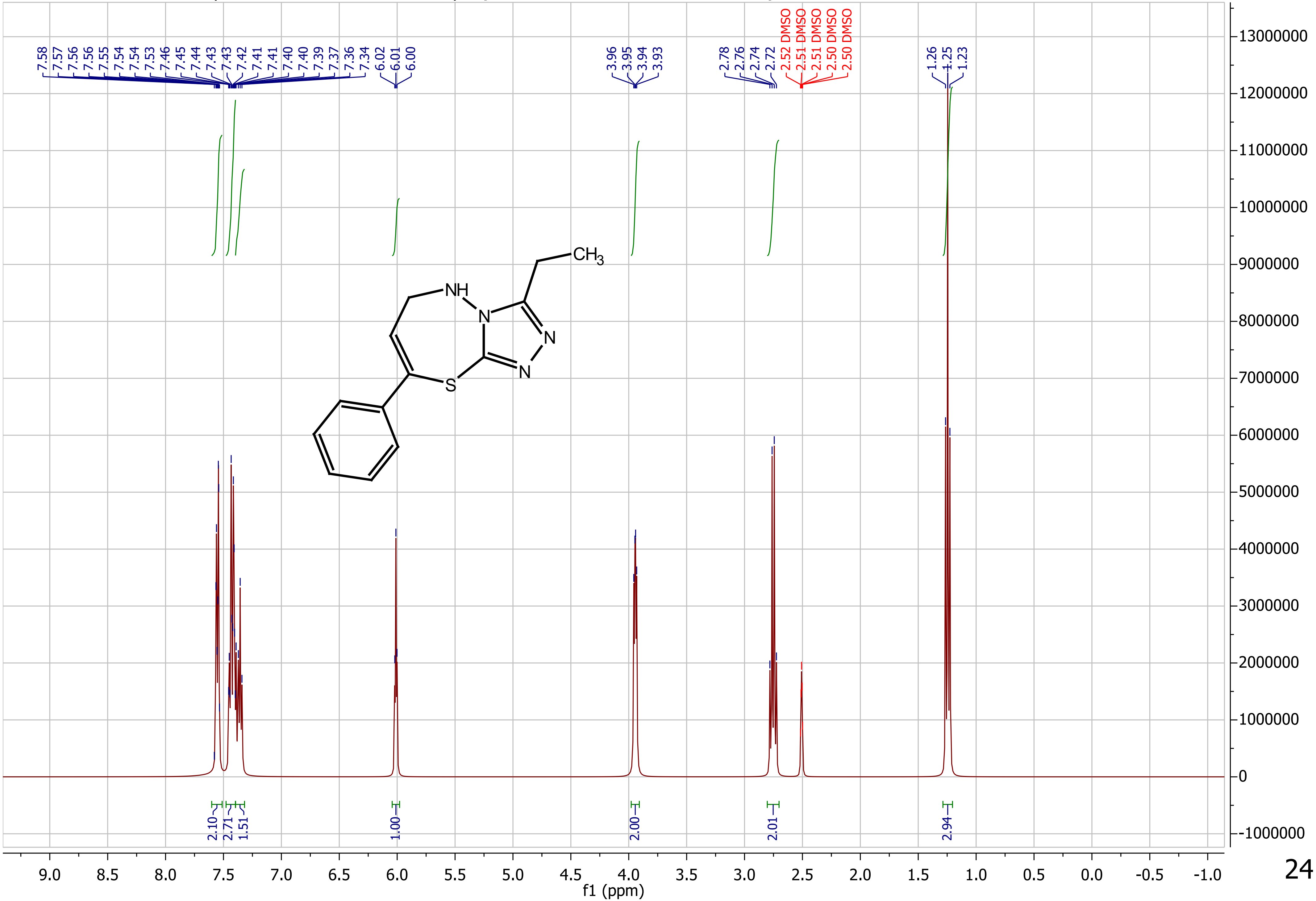

S23. ^1H NMR (400 MHz, DMSO-d_6) spectrum of the new compound **10d**

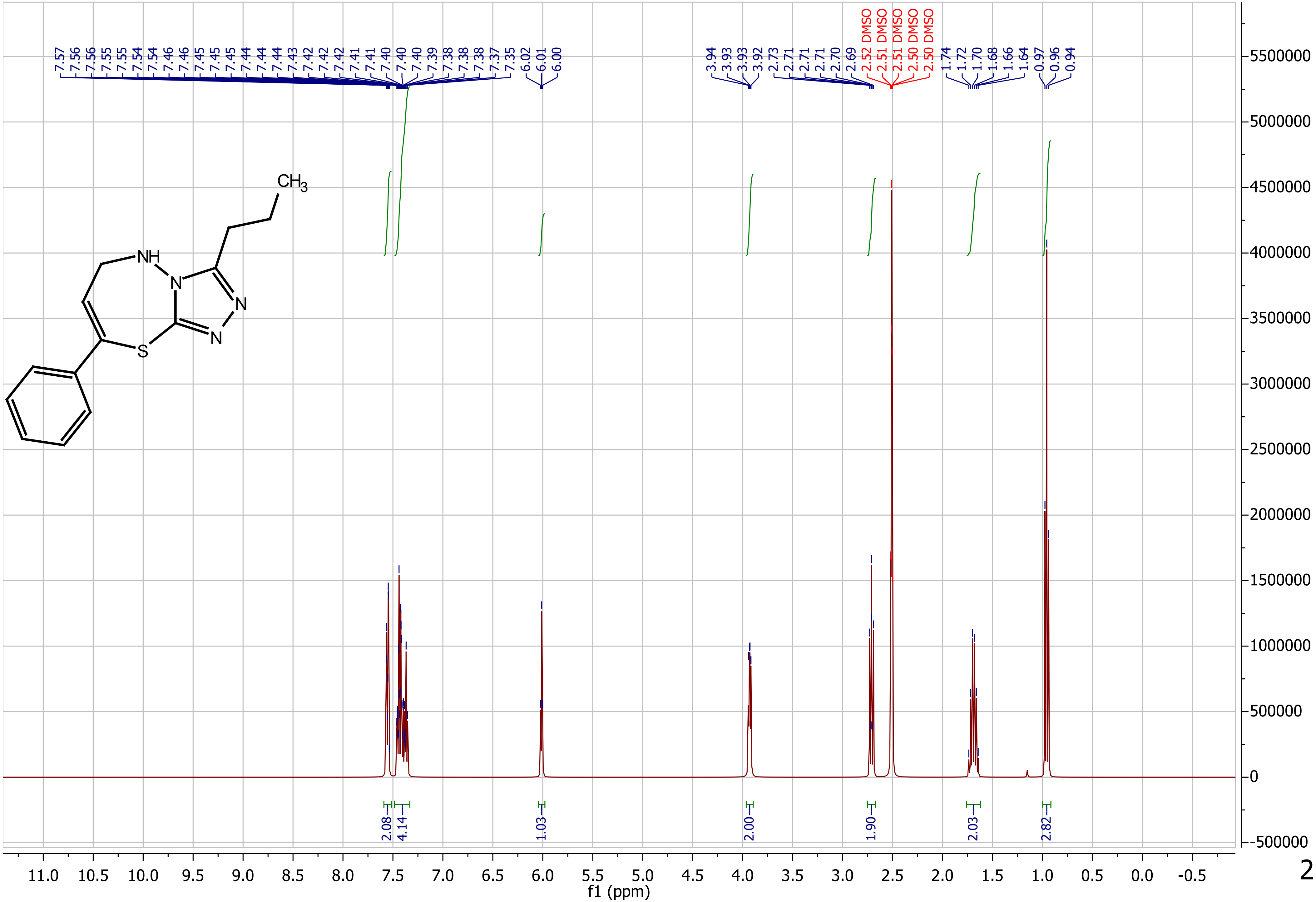

S24. ^{13}C NMR (101 MHz, DMSO-d_6) spectrum of the new compound **1a**

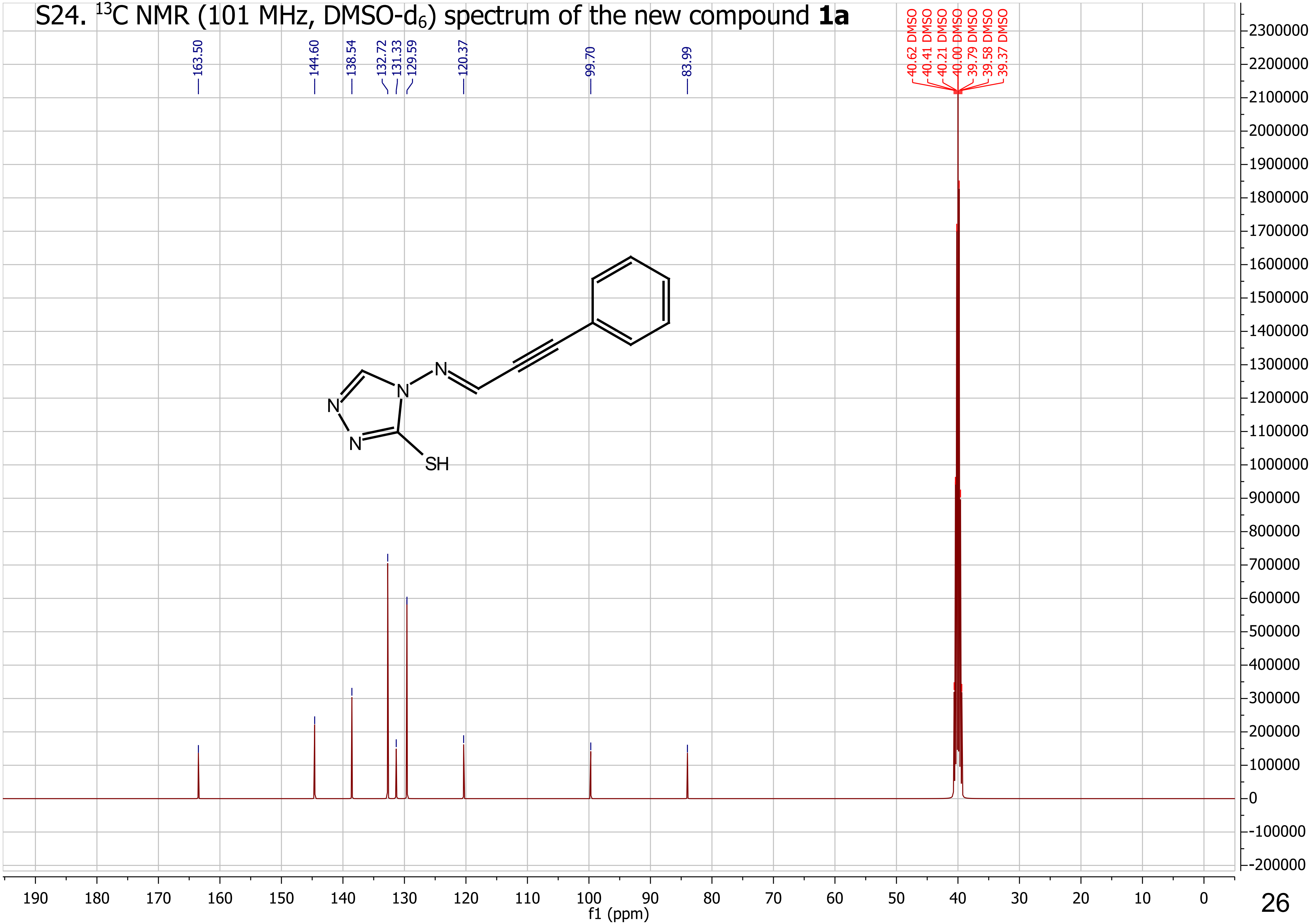

S25. ^{13}C NMR (101 MHz, DMSO-d_6) spectrum of the new compound **1b**

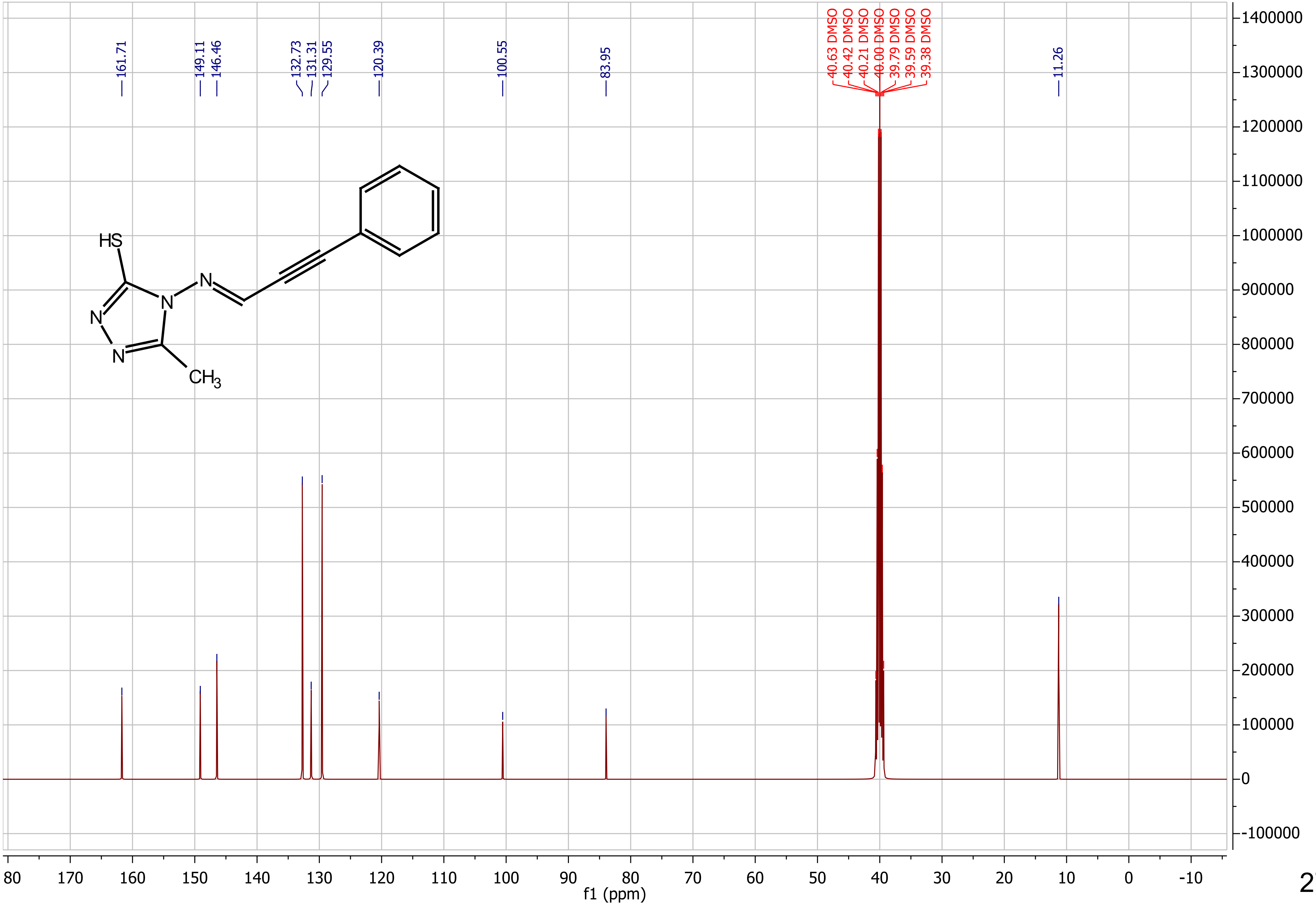

S26. ^{13}C NMR (101 MHz, DMSO-d_6) spectrum of the new compound **1c**

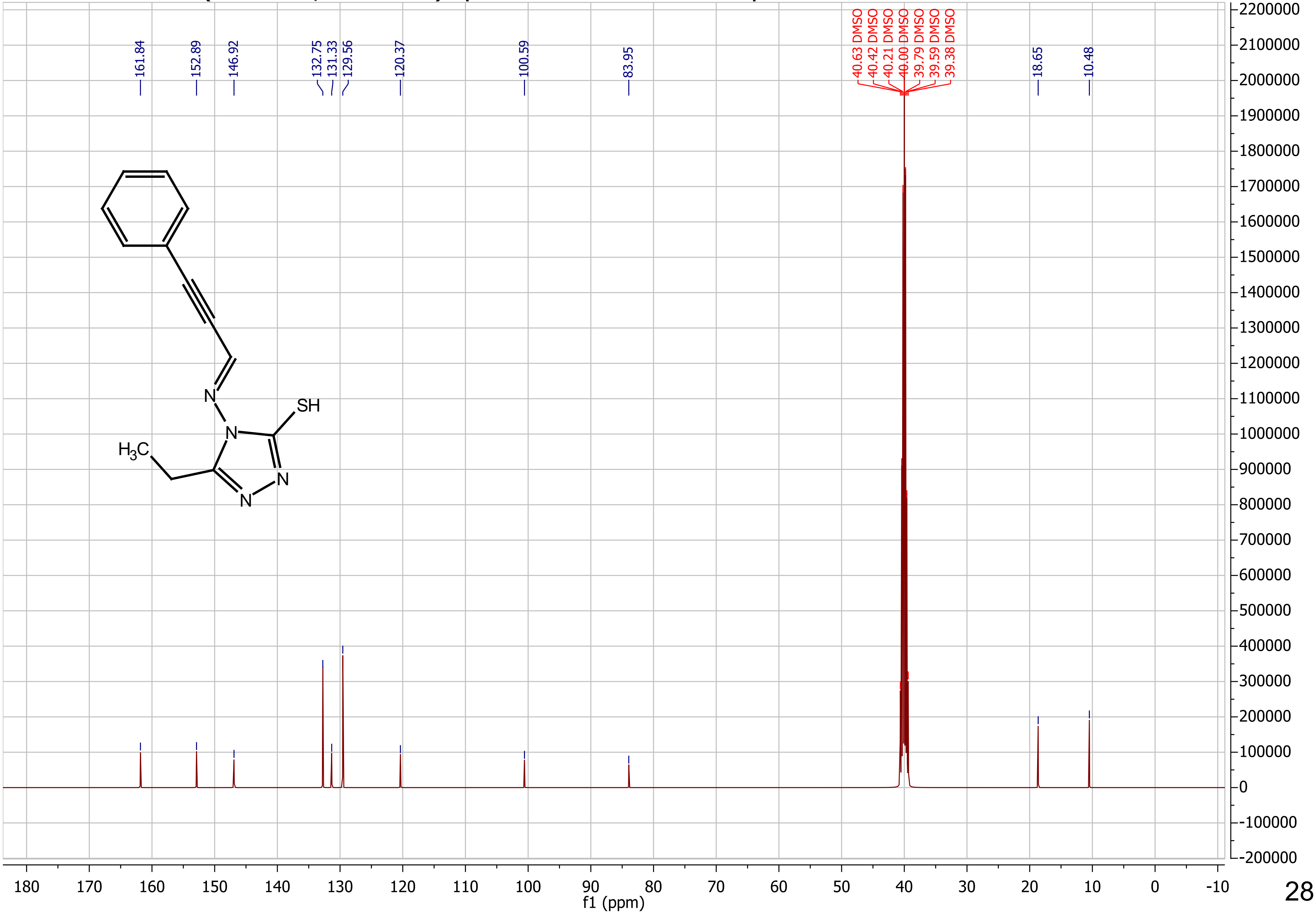

S27. ^{13}C NMR (101 MHz, DMSO-d_6) spectrum of the new compound **2a**

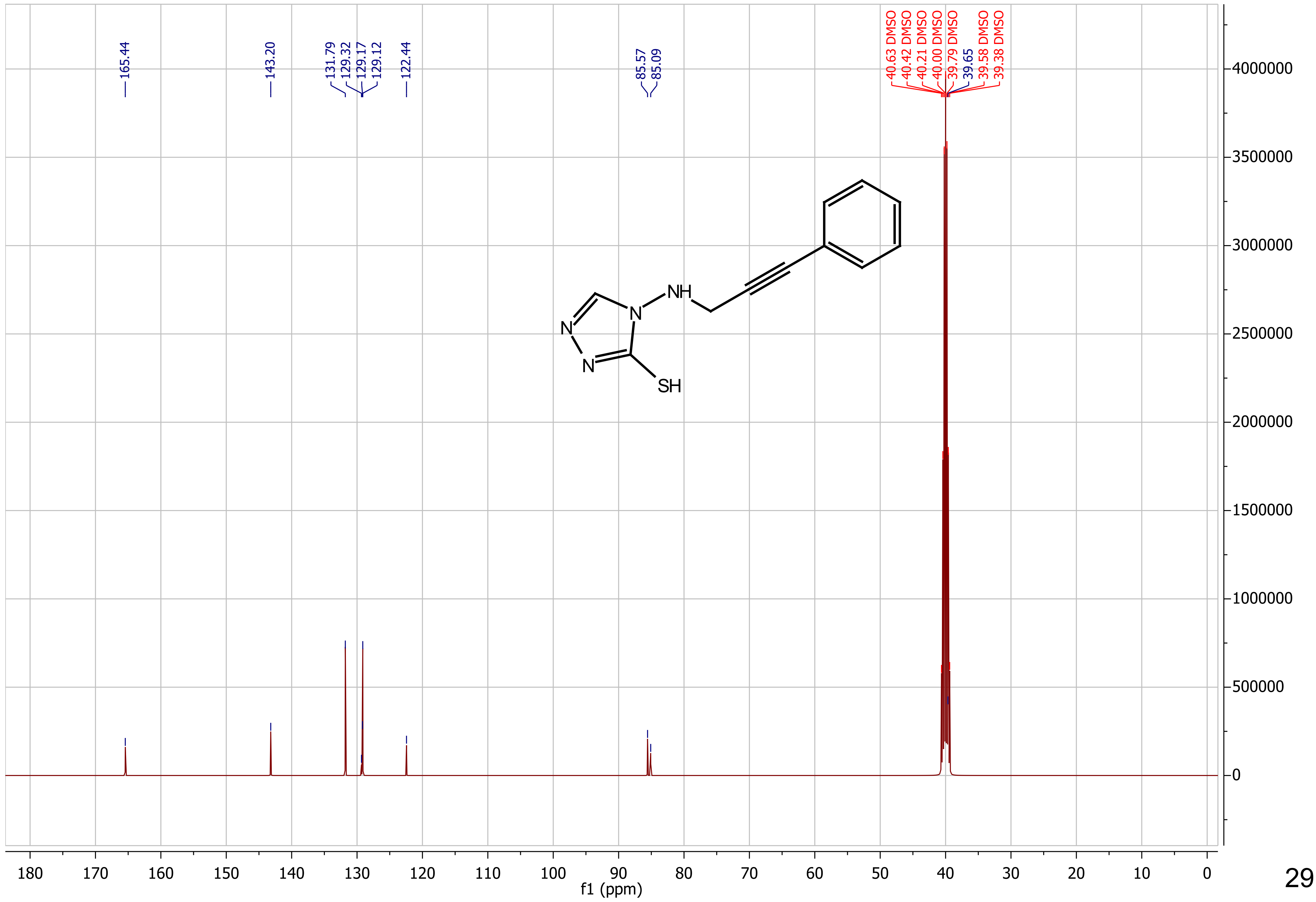

S28. ^{13}C NMR (101 MHz, DMSO-d_6) spectrum of the new compound **2b**

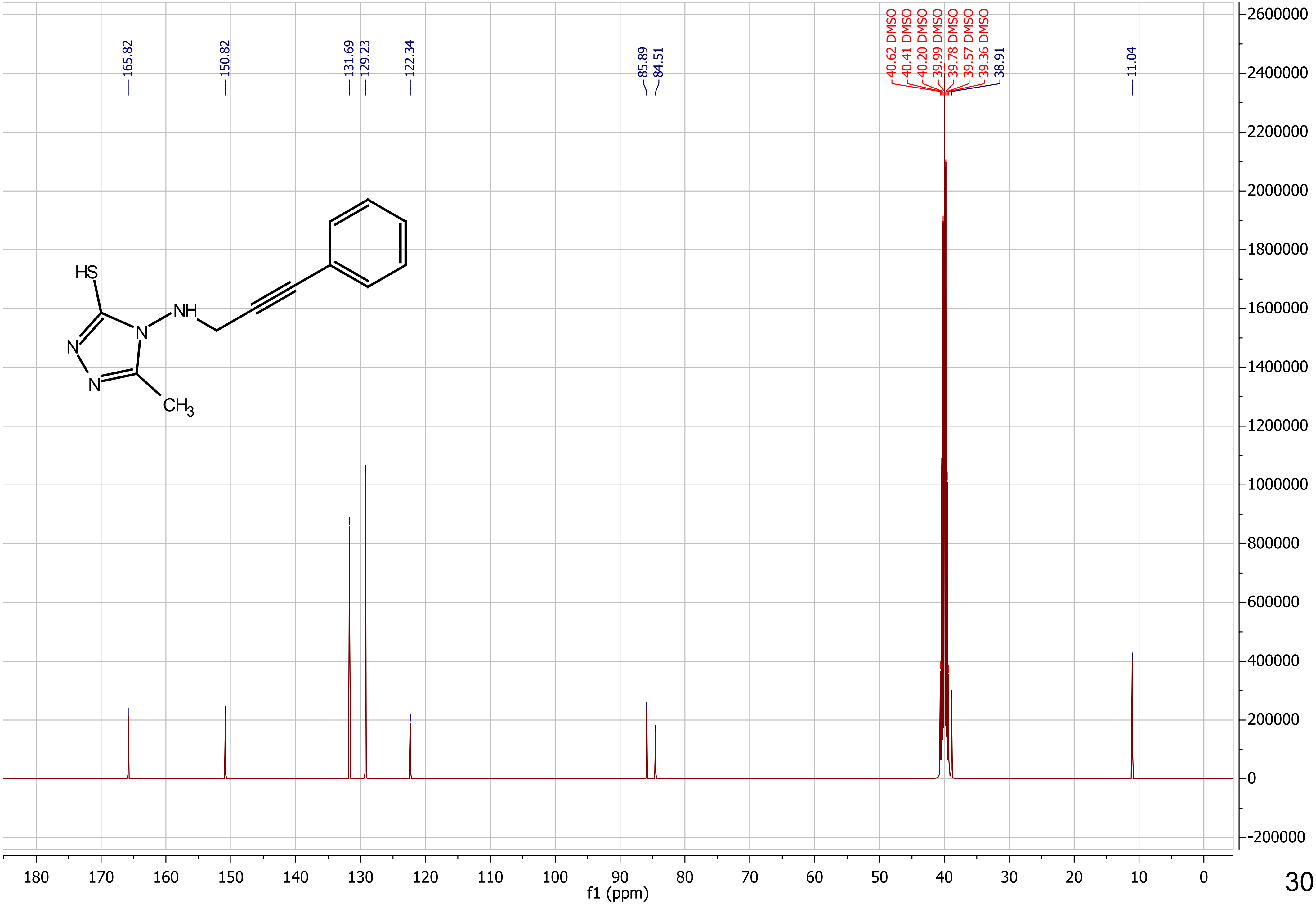

S29. ^{13}C NMR (101 MHz, DMSO-d_6) spectrum of the new compound **2c**

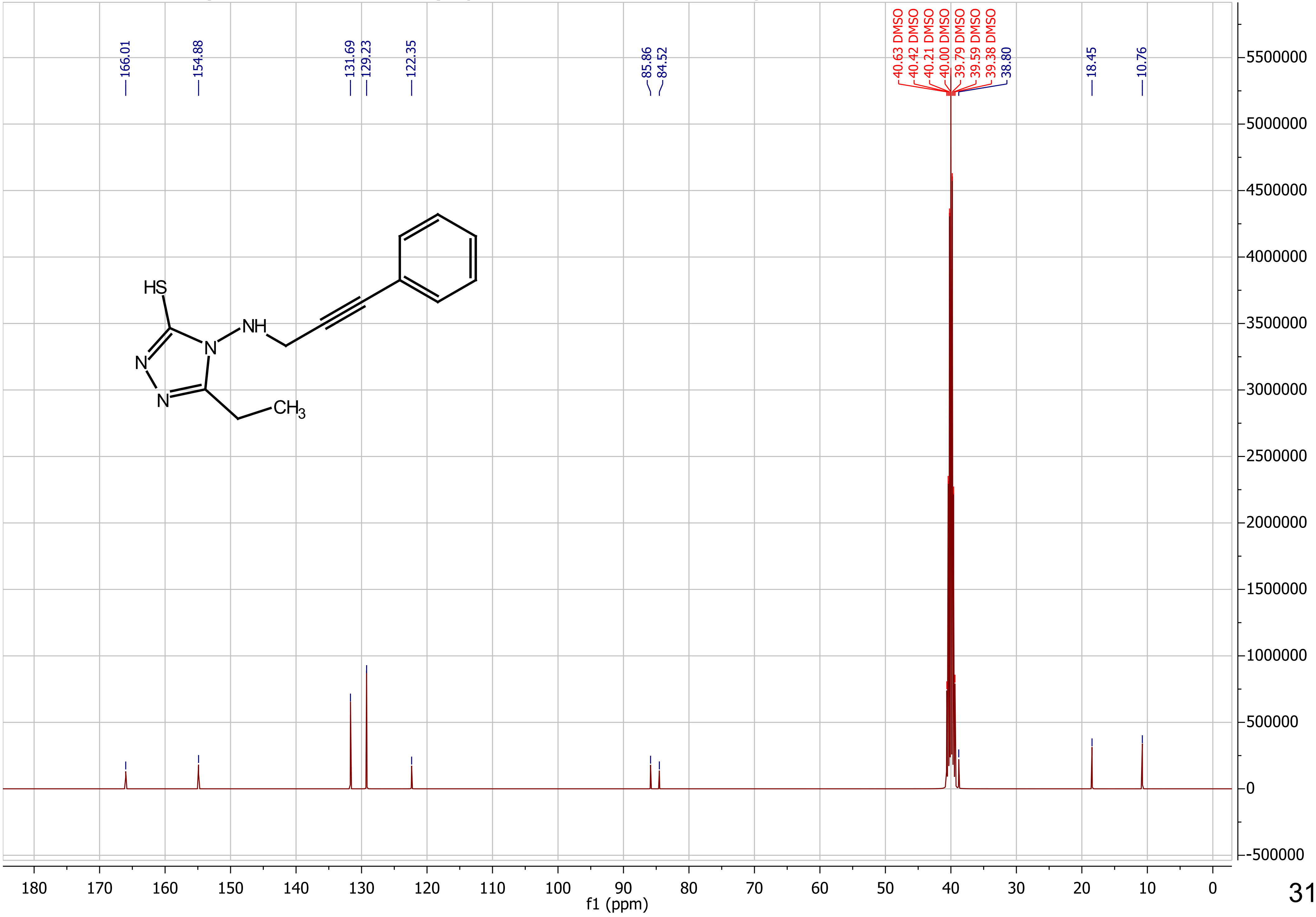

S30. ^{13}C NMR (101 MHz, DMSO-d_6) spectrum of the new compound **2d**

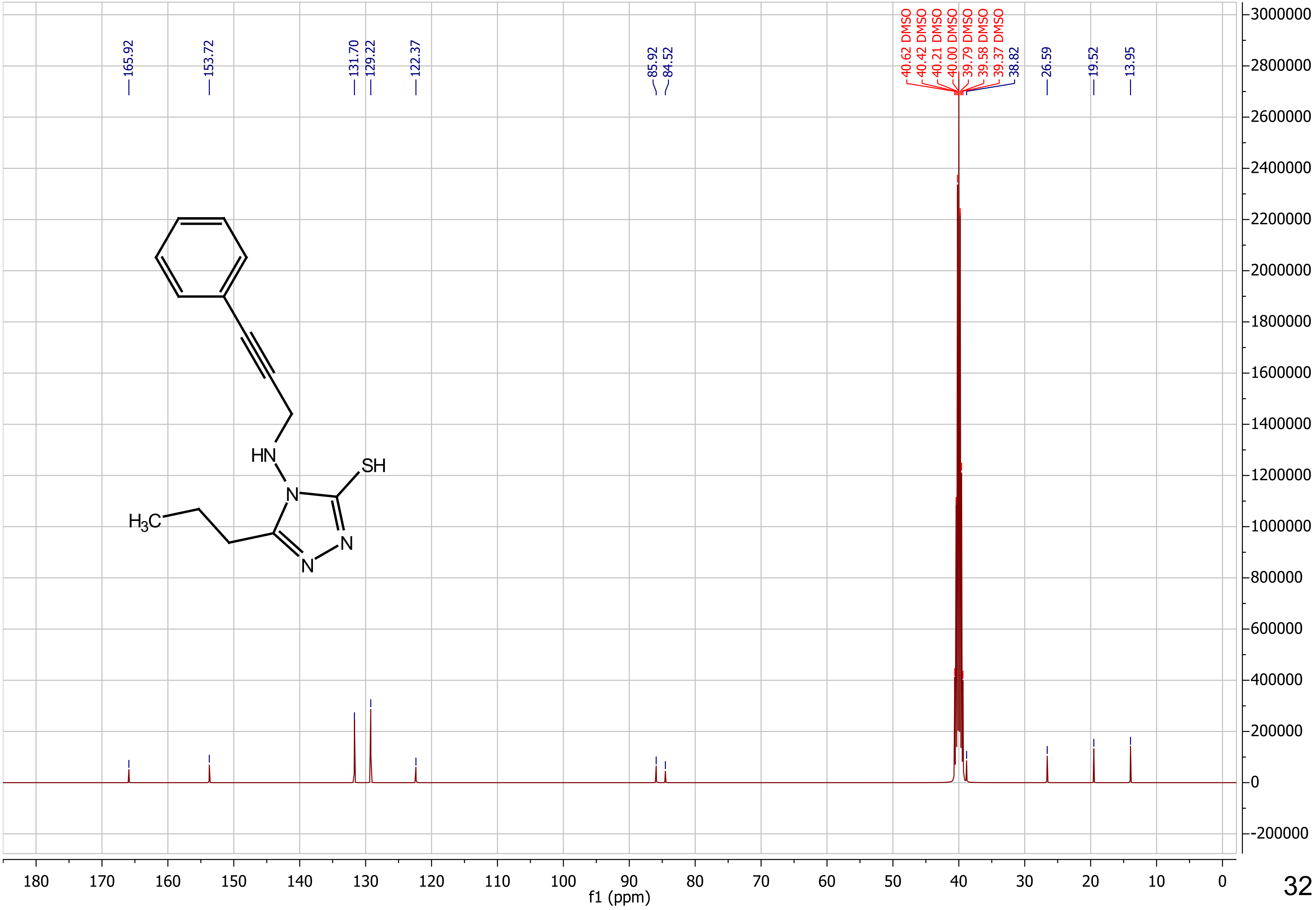

S31. ^{13}C NMR (101 MHz, DMSO-d_6) spectrum of the new compound **6a**

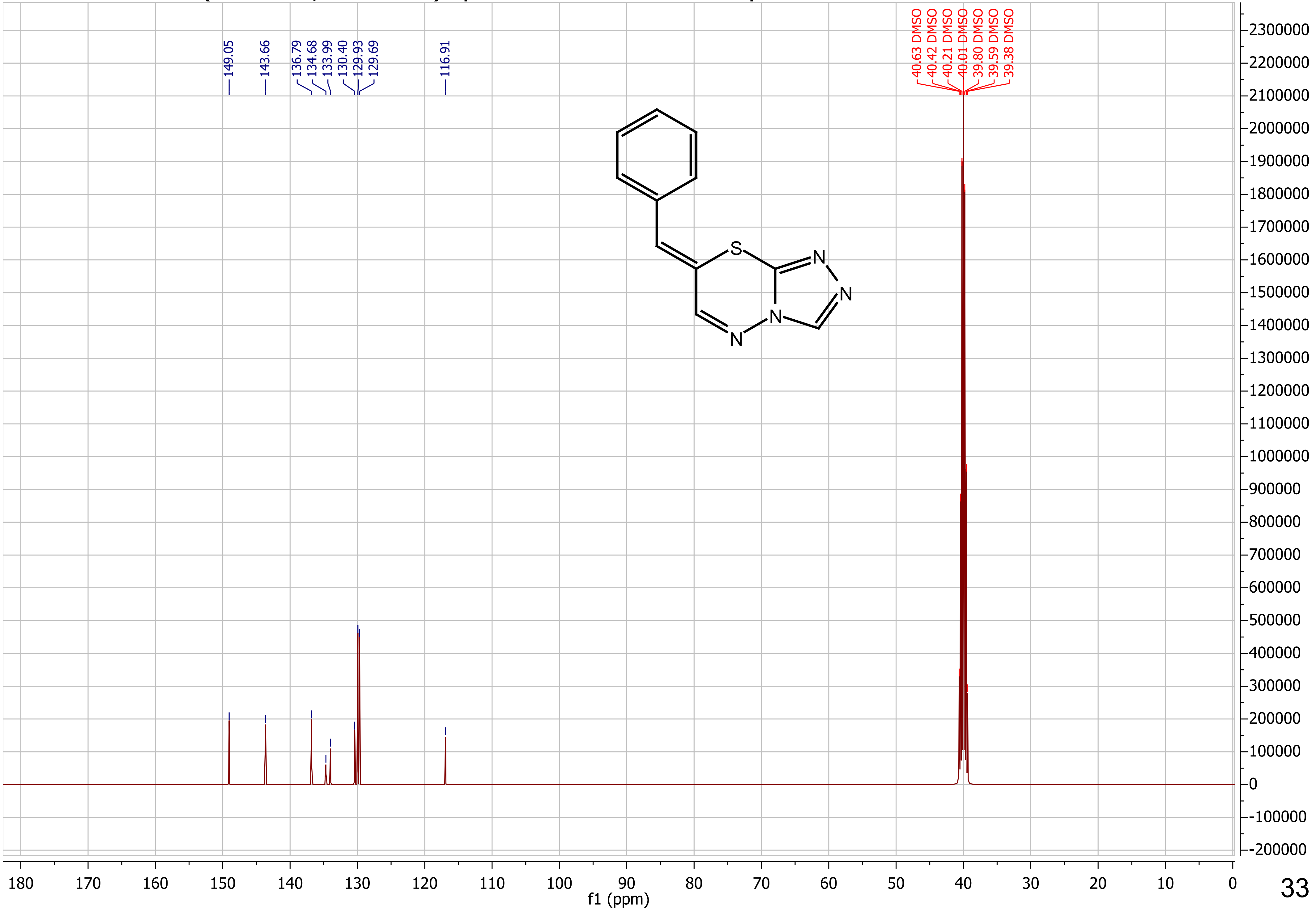

S32. ^{13}C NMR (101 MHz, DMSO-d_6) spectrum of the new compound **6b**

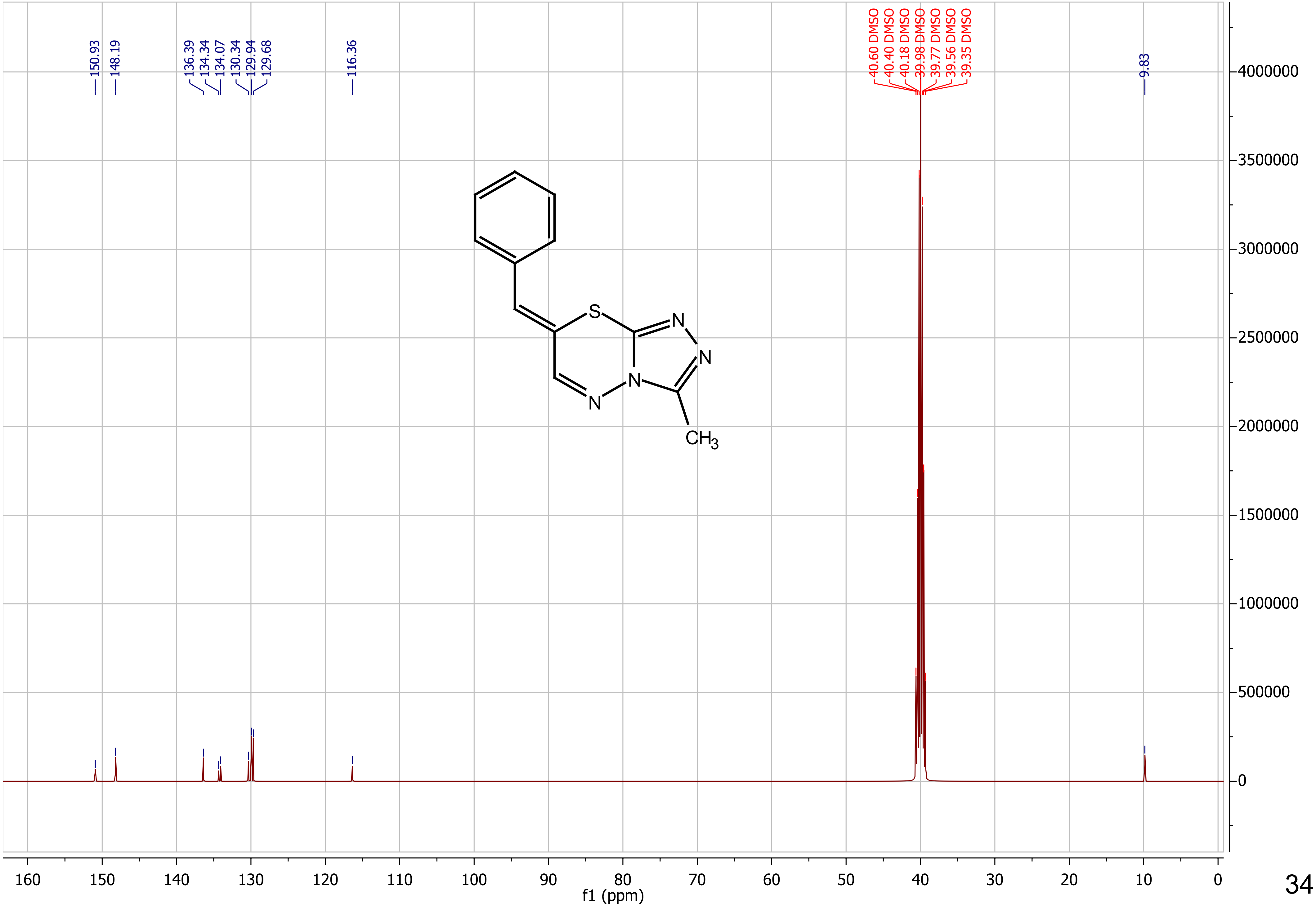

S33. ^{13}C NMR (101 MHz, DMSO-d_6) spectrum of the new compound **6c**

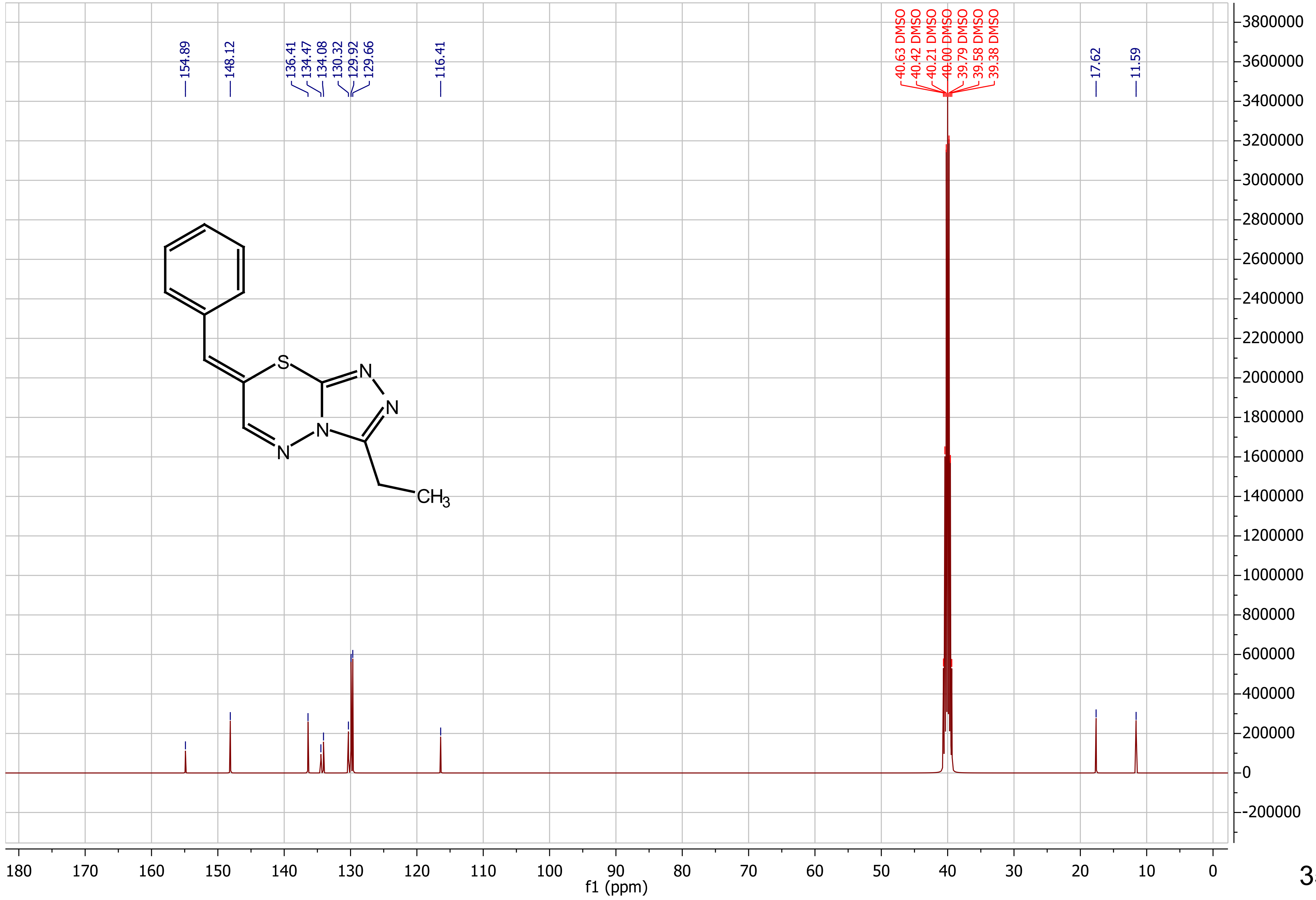

S34. ^{13}C NMR (101 MHz, DMSO-d_6) spectrum of the new compound **6d**

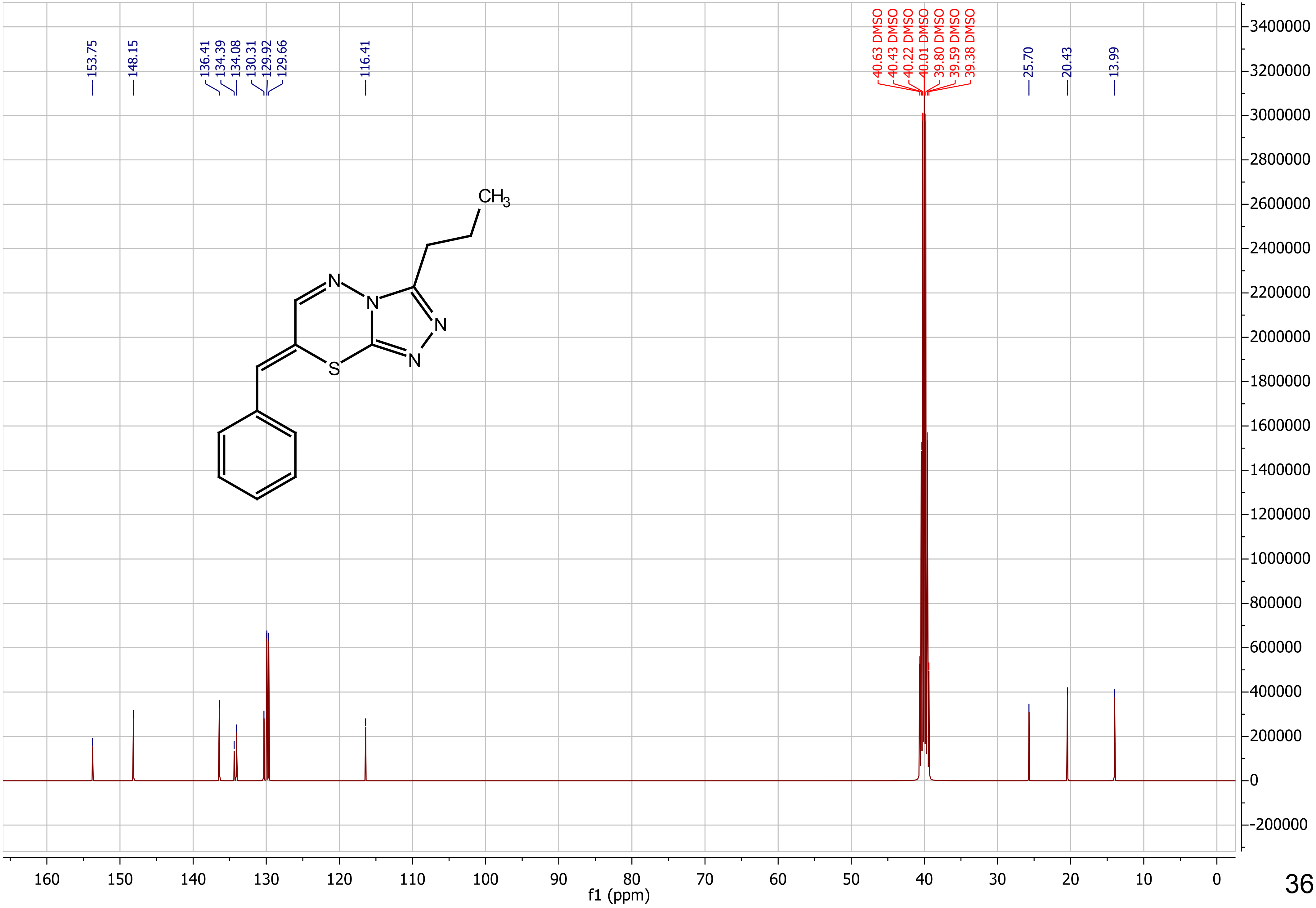

S35. ^{13}C NMR (101 MHz, DMSO-d_6) spectrum of the new compound **7a**

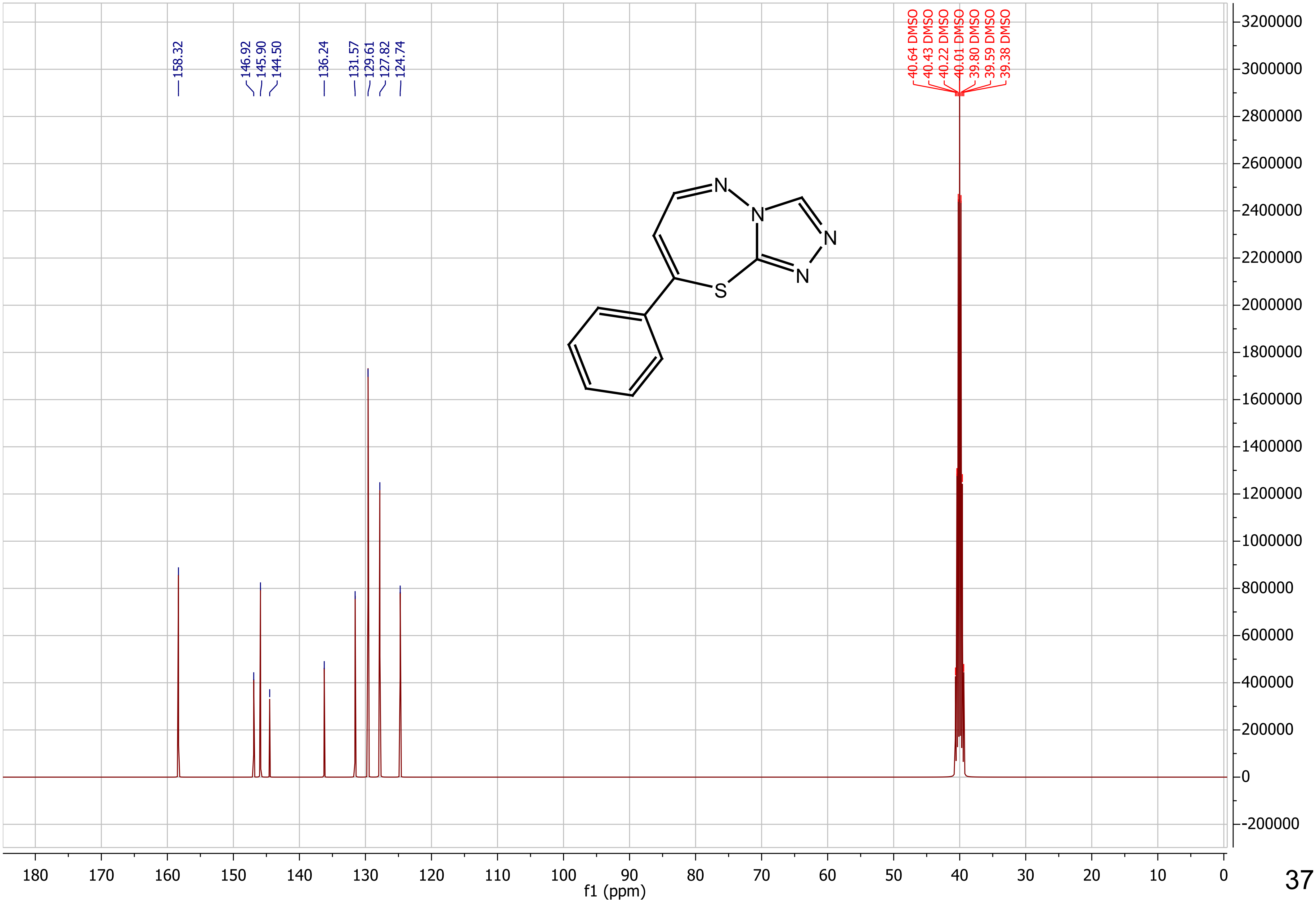

S36. ^{13}C NMR (101 MHz, DMSO-d_6) spectrum of the new compound **7b**

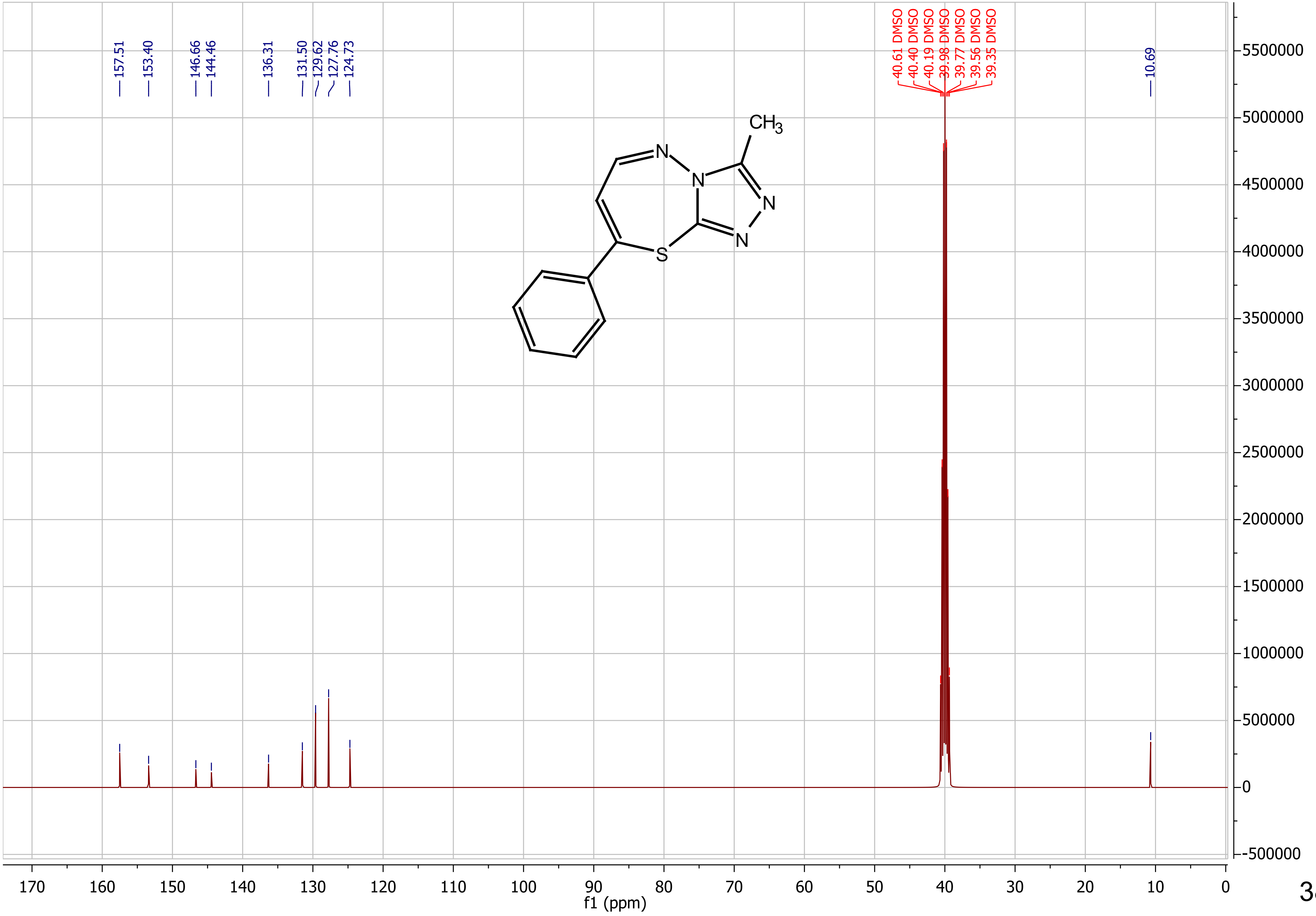

S37. ^{13}C NMR (101 MHz, DMSO-d_6) spectrum of the new compound **7c**

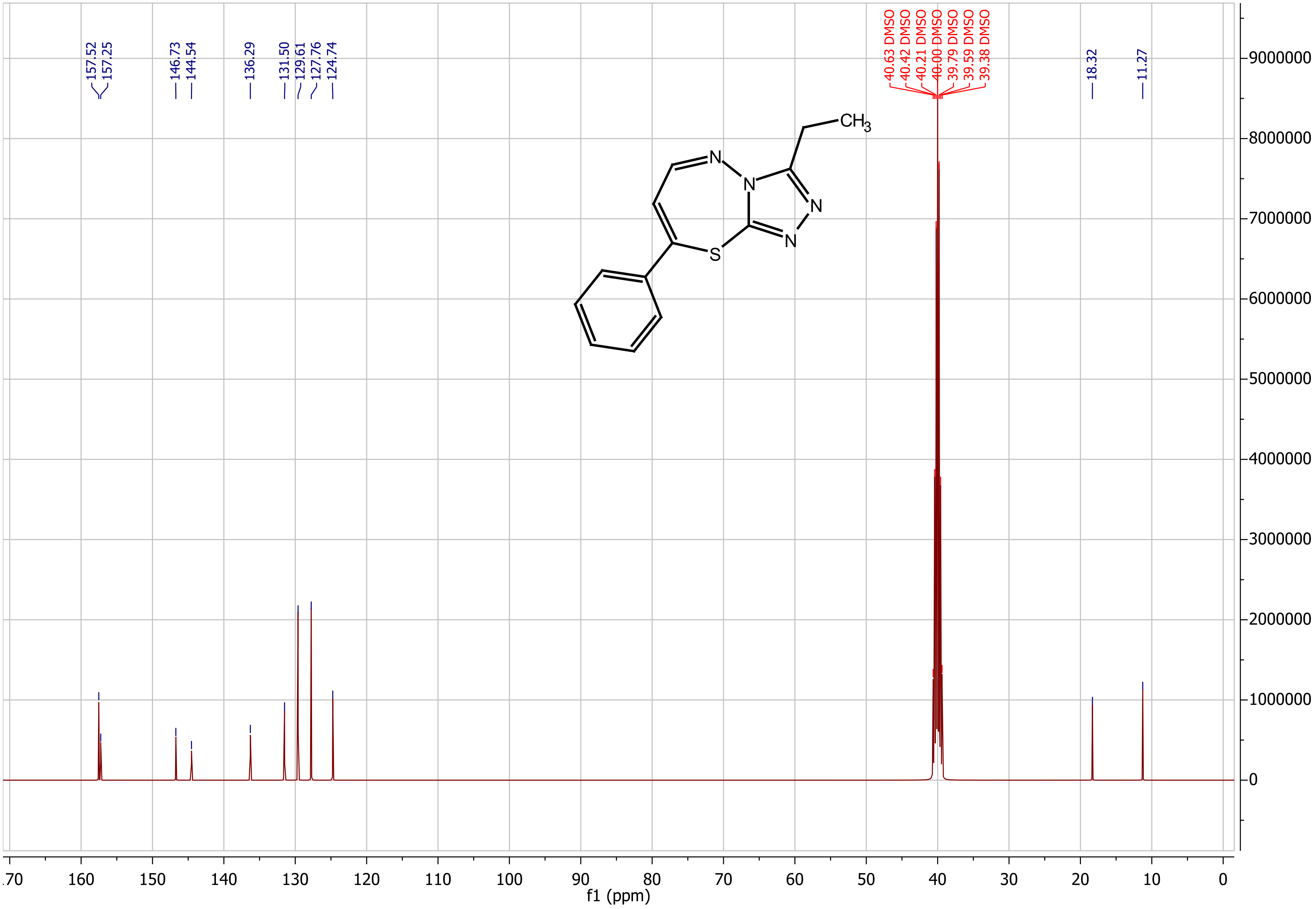

S38. ^{13}C NMR (101 MHz, DMSO-d_6) spectrum of the new compound **7d**

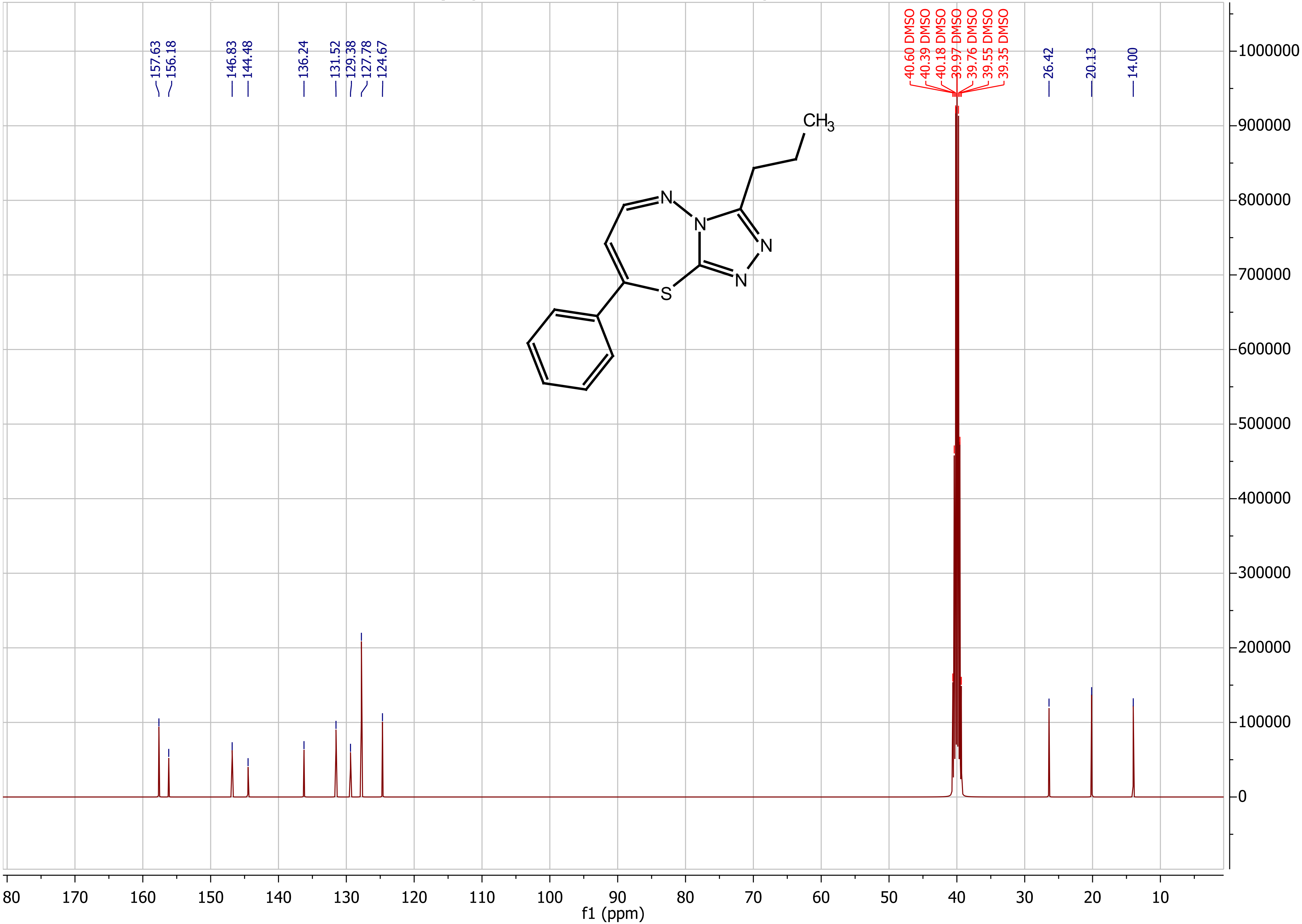

S39. ^{13}C NMR (101 MHz, DMSO-d_6) spectrum of the new compound **8a**

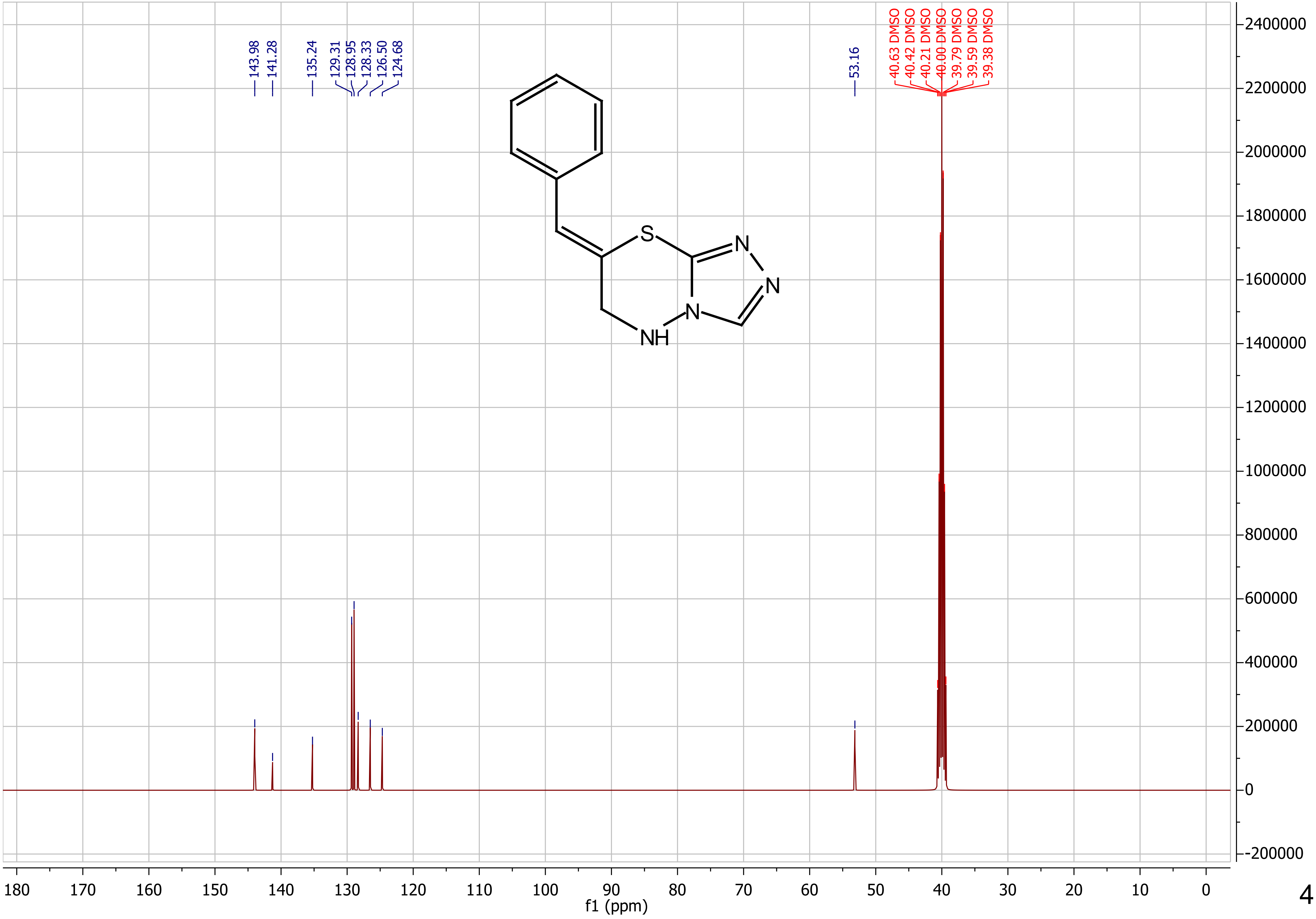

S40. ^{13}C NMR (101 MHz, DMSO-d_6) spectrum of the new compound **8b**

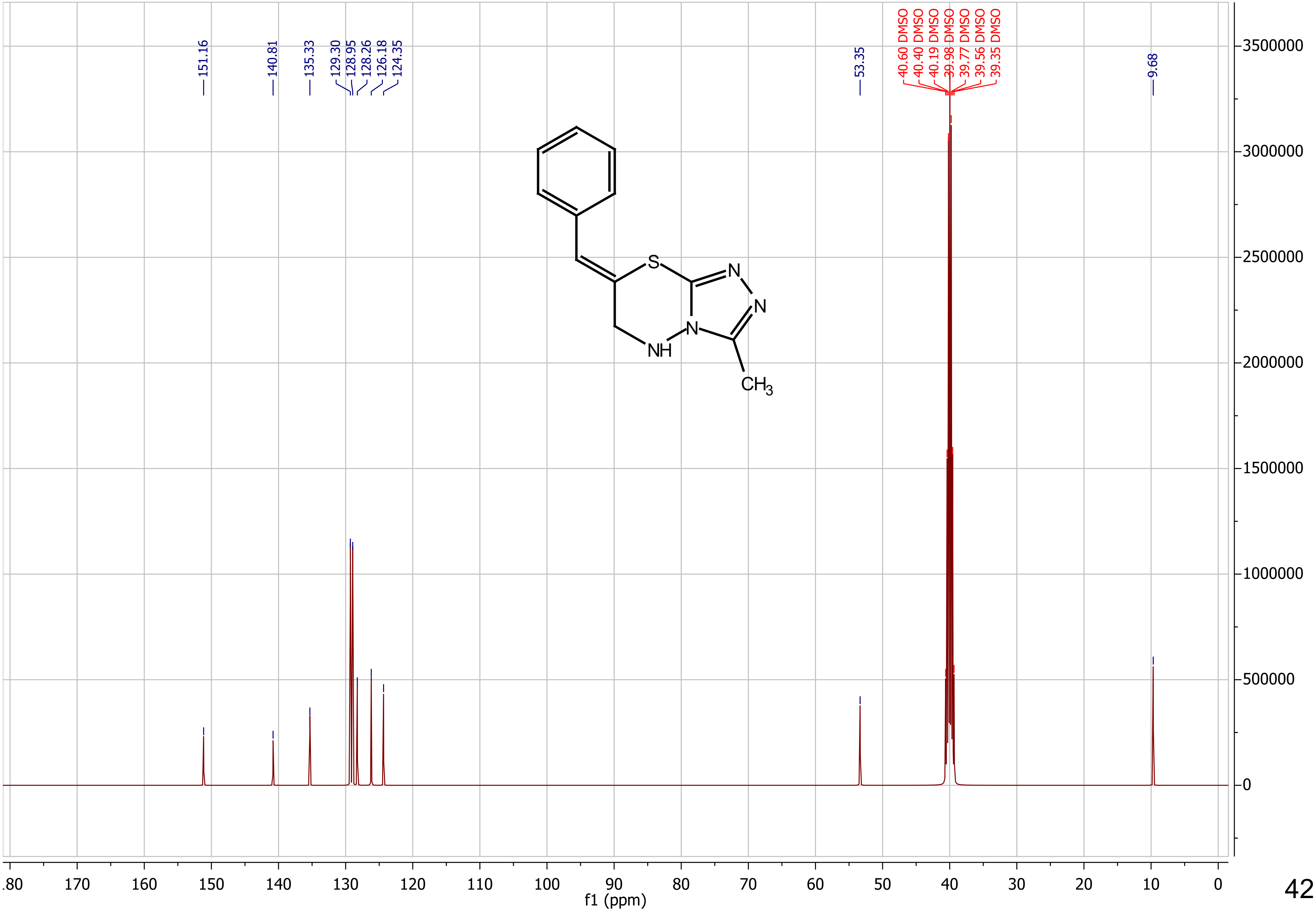

S41. ^{13}C NMR (101 MHz, DMSO-d_6) spectrum of the new compound **8c**

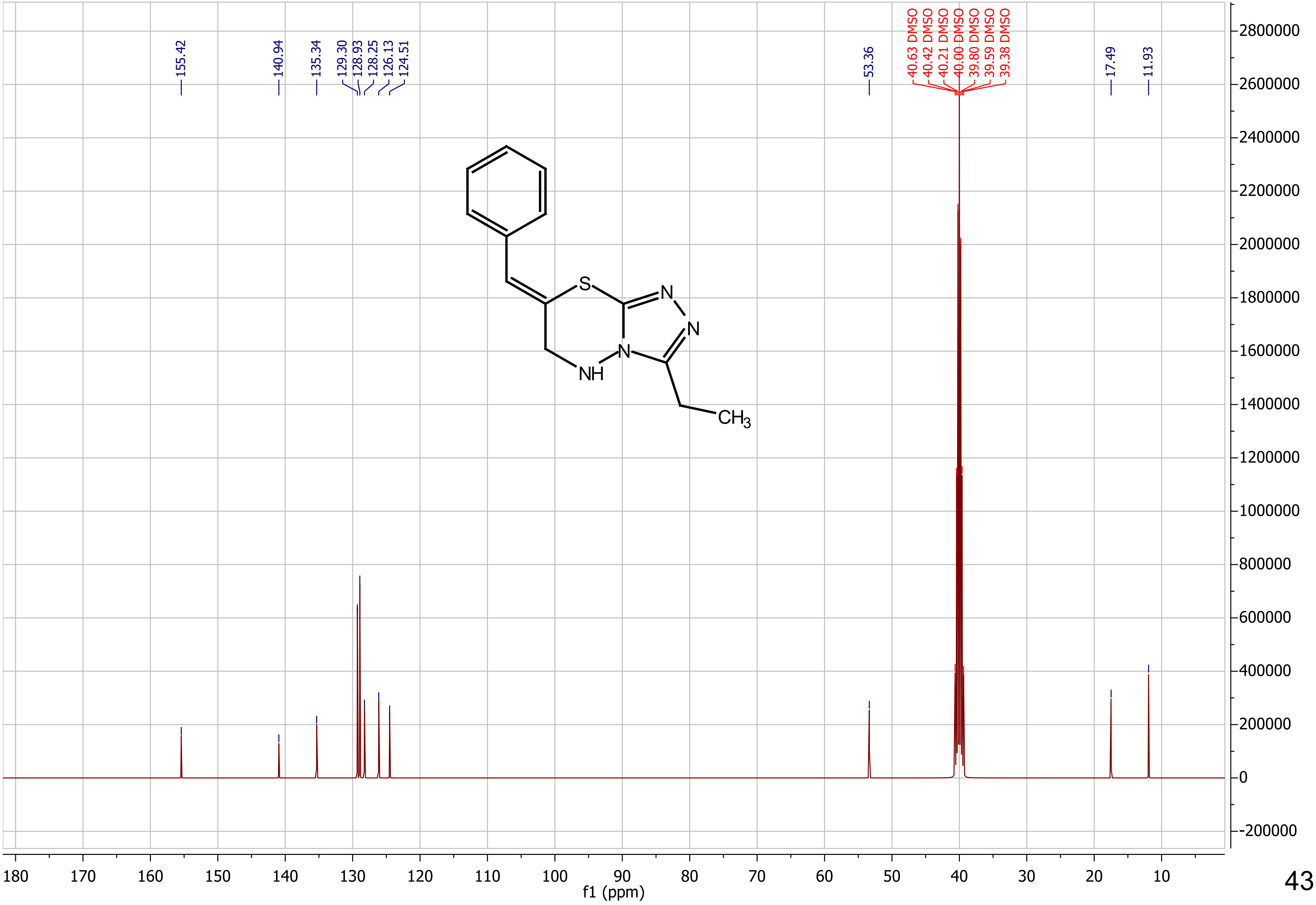

S42. ^{13}C NMR (101 MHz, DMSO-d_6) spectrum of the new compound **8d**

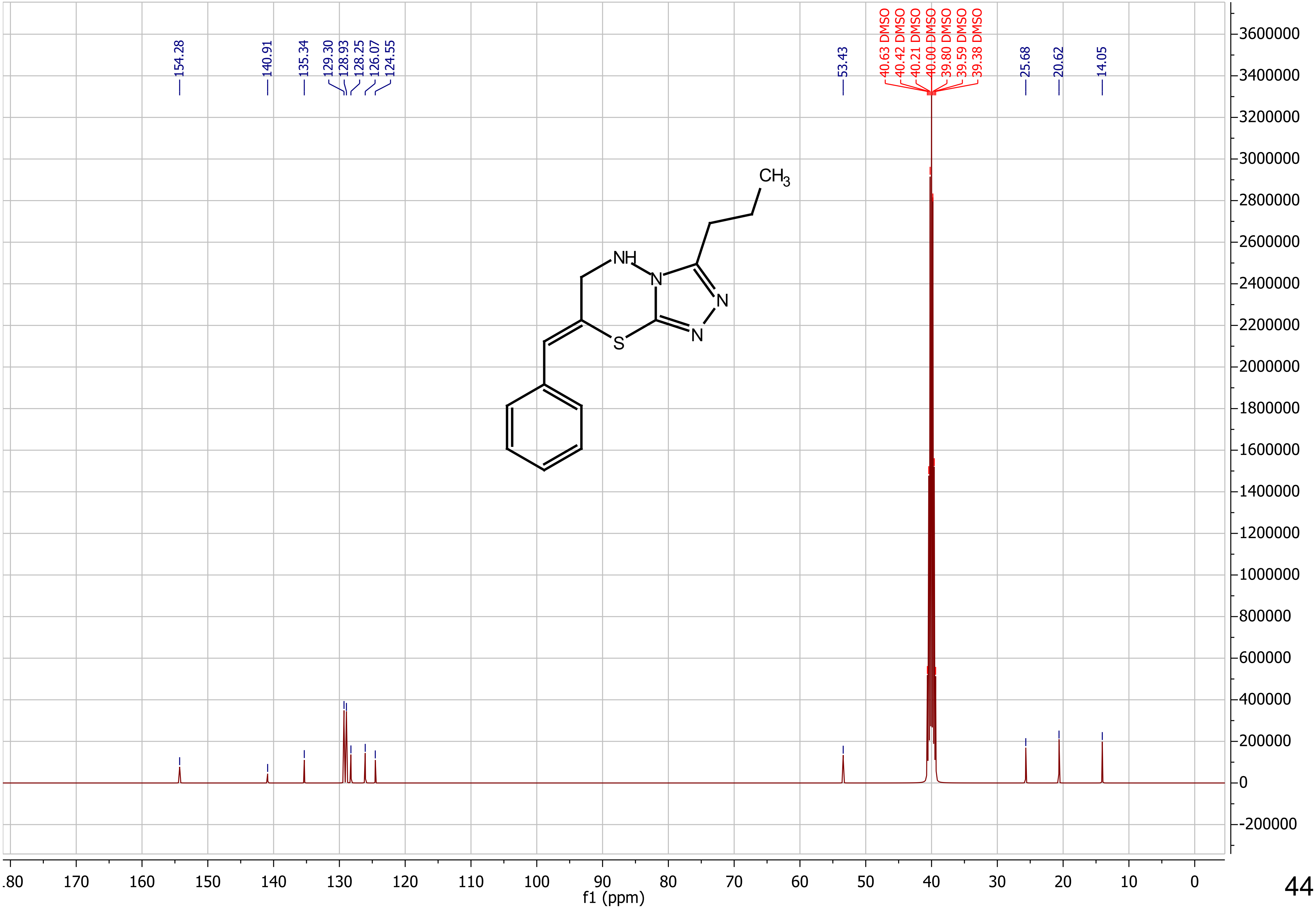

S43. ^{13}C NMR (101 MHz, DMSO-d_6) spectrum of the new compound **10a**

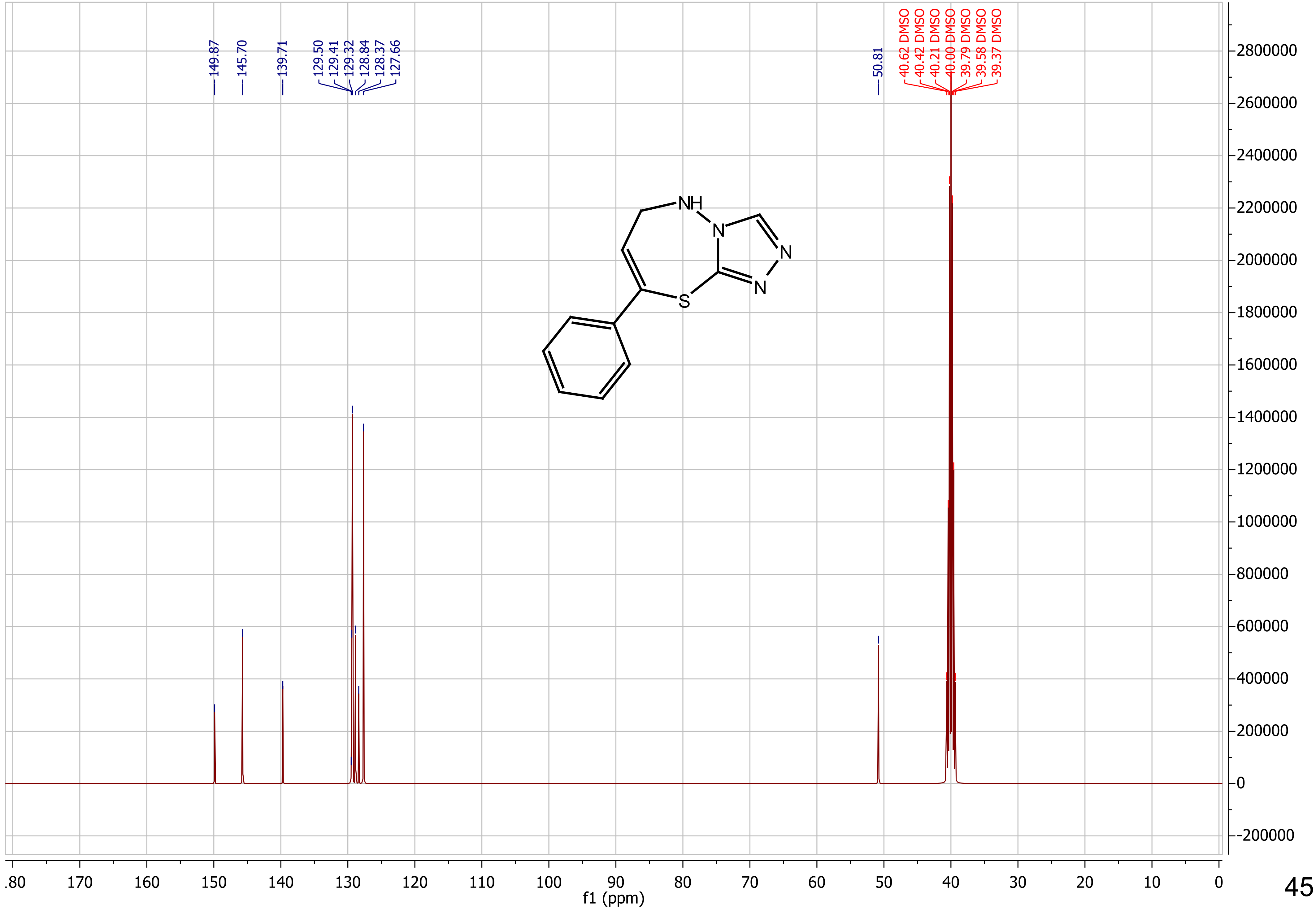

S44. ^{13}C NMR (101 MHz, DMSO-d_6) spectrum of the new compound **10b**

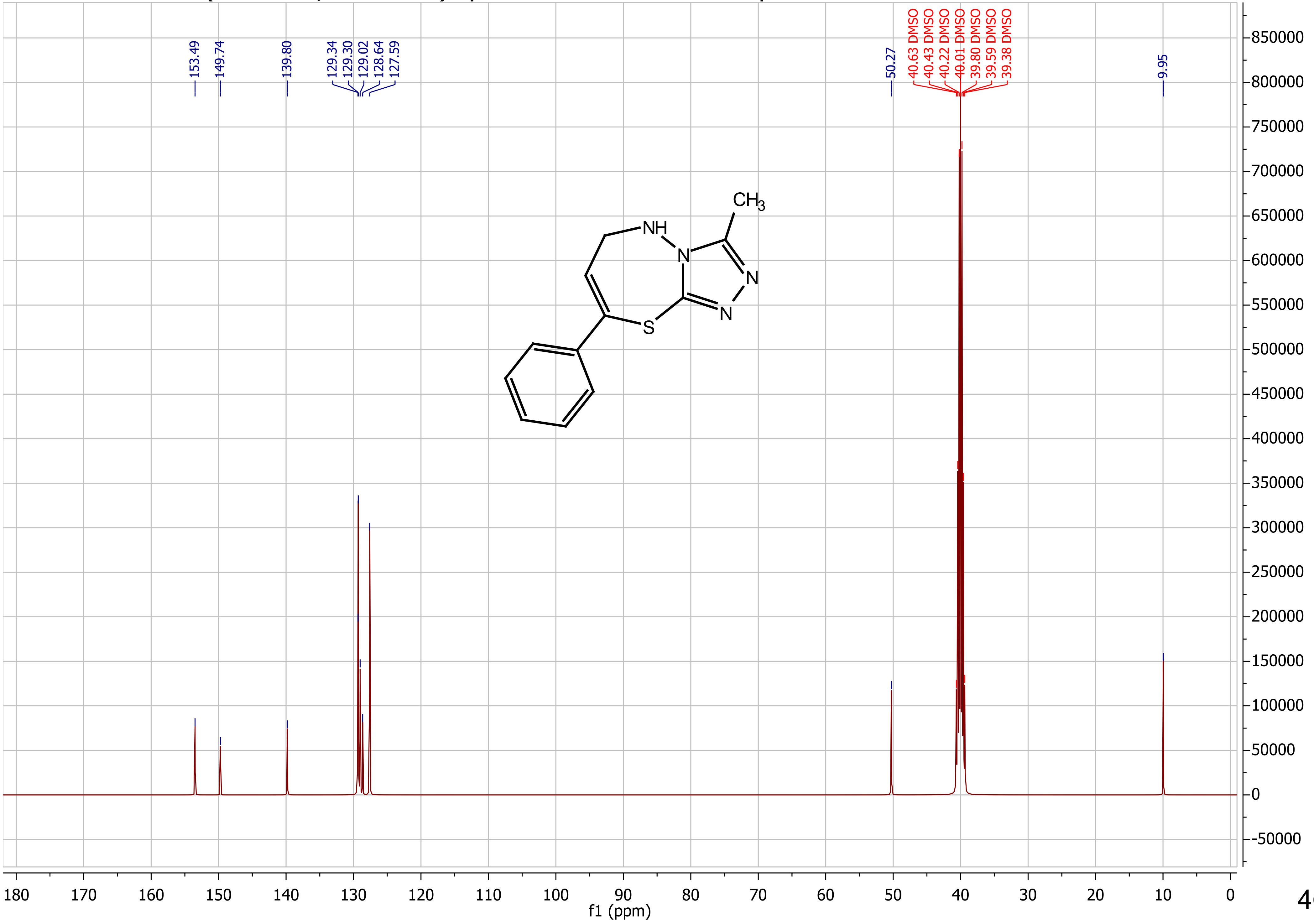

S45. ^{13}C NMR (101 MHz, DMSO-d_6) spectrum of the new compound **10c**

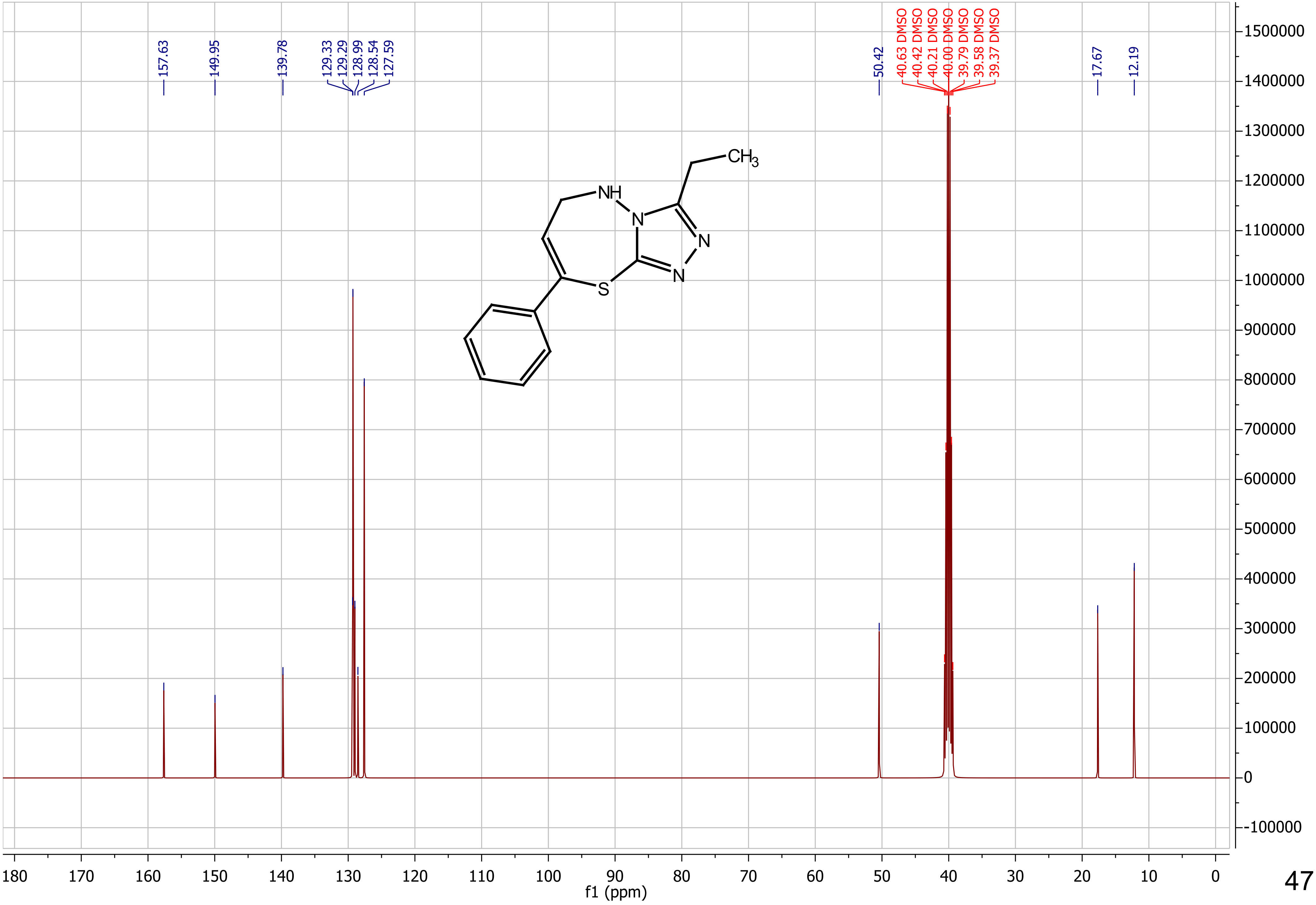

S46. ^{13}C NMR (101 MHz, DMSO-d_6) spectrum of the new compound **10d**

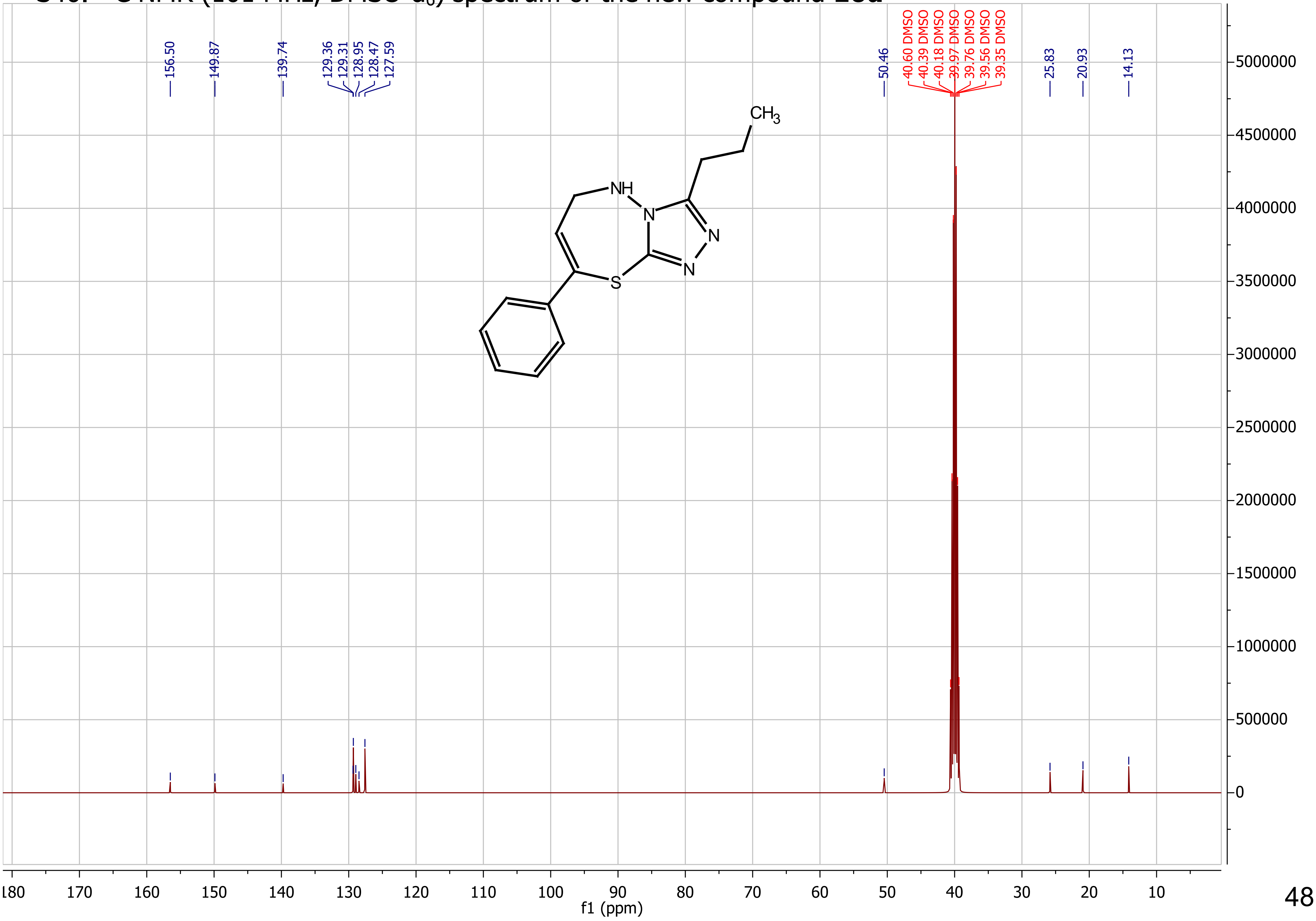

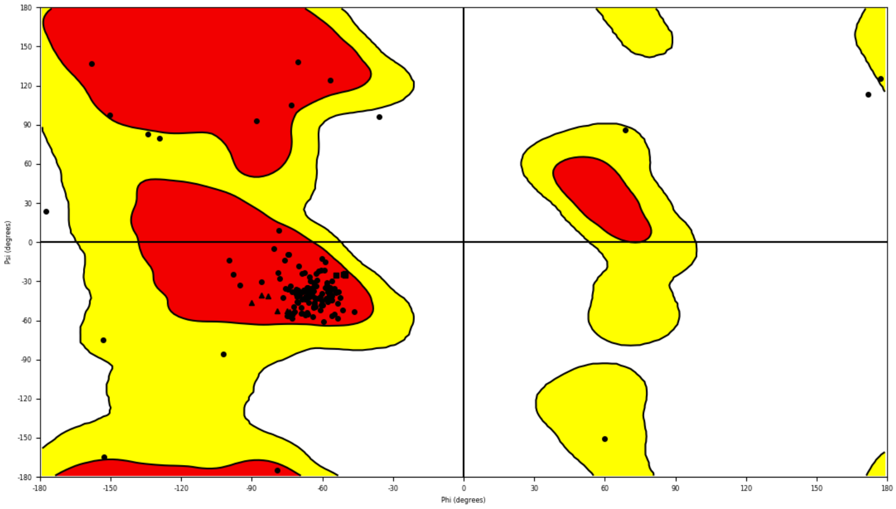

S47. Ramachandran plot for the engineered model of M2 channel (strain A/Puerto Rico/8/1934 H1N1)
